# Canonical and Non-Canonical Antipsychotics’ Dopamine-Related Mechanisms of Present and Next Generation Molecules: A Systematic Review on Translational Highlights for Treatment Response and Treatment-Resistant Schizophrenia

**DOI:** 10.3390/ijms24065945

**Published:** 2023-03-21

**Authors:** Andrea de Bartolomeis, Mariateresa Ciccarelli, Giuseppe De Simone, Benedetta Mazza, Annarita Barone, Licia Vellucci

**Affiliations:** Section of Psychiatry, Laboratory of Translational and Molecular Psychiatry and Unit of Treatment-Resistant Psychosis, Department of Neuroscience, Reproductive Sciences and Dentistry, University Medical School of Naples “Federico II”, 80131 Naples, Italy

**Keywords:** treatment-resistant schizophrenia, antipsychotics, postsynaptic density, dopamine, glutamate, synaptopathy, PSD-95, Homer

## Abstract

Schizophrenia is a severe psychiatric illness affecting almost 25 million people worldwide and is conceptualized as a disorder of synaptic plasticity and brain connectivity. Antipsychotics are the primary pharmacological treatment after more than sixty years after their introduction in therapy. Two findings hold true for all presently available antipsychotics. First, all antipsychotics occupy the dopamine D2 receptor (D2R) as an antagonist or partial agonist, even if with different affinity; second, D2R occupancy is the necessary and probably the sufficient mechanism for antipsychotic effect despite the complexity of antipsychotics’ receptor profile. D2R occupancy is followed by coincident or divergent intracellular mechanisms, implying the contribution of cAMP regulation, β-arrestin recruitment, and phospholipase A activation, to quote some of the mechanisms considered canonical. However, in recent years, novel mechanisms related to dopamine function beyond or together with D2R occupancy have emerged. Among these potentially non-canonical mechanisms, the role of Na^2+^ channels at the dopamine at the presynaptic site, dopamine transporter (DAT) involvement as the main regulator of dopamine concentration at synaptic clefts, and the putative role of antipsychotics as chaperones for intracellular D2R sequestration, should be included. These mechanisms expand the fundamental role of dopamine in schizophrenia therapy and may have relevance to considering putatively new strategies for treatment-resistant schizophrenia (TRS), an extremely severe condition epidemiologically relevant and affecting almost 30% of schizophrenia patients. Here, we performed a critical evaluation of the role of antipsychotics in synaptic plasticity, focusing on their canonical and non-canonical mechanisms of action relevant to the treatment of schizophrenia and their subsequent implication for the pathophysiology and potential therapy of TRS.

## 1. Introduction

Dopamine cortical–subcortical dysregulation epitomizes the main, albeit not the only, neurotransmitter landmark of schizophrenia pathophysiology and antipsychotics remain, more than sixty years after their introduction in therapy, the cornerstone of schizophrenia treatment.

Emerging findings from novel methodologies of receptor–ligand computational and Fluorescence Resonance Energy Transfer (FRET) microscopy modeling at the dopamine D2 receptor (D2R) [1], analysis of antipsychotics effects on quantal dopamine release at the presynaptic site [2], and in vivo studies of dopamine metabolism by Positron Emission Tomography (PET) imaging have unveiled previously unexplored landscape of antipsychotics—dopamine dynamics [3]—calling for a re-evaluation of the canonical dopamine-based mechanism of this class of drugs [4].

Antipsychotics are the mainstay of the pharmacological treatment of schizophrenia and are also prescribed for mania in bipolar disorder as well as, to a lesser extent, augmentation therapy in treatment-resistant depression. Preclinical, clinical, and human functional studies strongly support the occupancy of D2R as a common, necessary, and perhaps sufficient mechanism of action (MOA) for all molecules available at present in clinics, even if they are characterized by different receptor profiles. This mechanism is coherent with a “dopaminocentric hypothesis” of psychosis that was conceptualized almost 50 years ago and is still considered fundamental [5,6,7], even if relevant updates and revisitation of findings from imaging [8,9,10], post-mortem [11,12], and modeling studies [13] have incorporated other neurotransmitter mechanisms, primarily the glutamatergic ones. However, the same findings call for a more stringent re-evaluation of antipsychotics’ MOA within the dopamine system, starting from the main target: the D2R. A critical appraisal of the non-canonical dopamine effects of antipsychotics is not only of significative heuristic value but even more relevant for the translational possibility of exploring novel therapeutic strategies.

Novel strategies are even more needed with reference to those patients who do not respond to treatment with antipsychotics using at least two antipsychotics (one should be an atypical or new generation drug) at a dose of 600 mg/equivalent of chlorpromazine for at least 6 weeks of treatment, these patients are defined as treatment-resistant [14]. The only available drug for these patients is, after more than fifty years, clozapine.

Considering that (i) at present, the dopaminergic strategy to tackle psychosis has been demonstrated to be effective, at least for positive symptoms, (ii) D2R occupancy could be limited by the potential onset of motor and other adverse events, and (iii) multiple domains of the disorder are indirectly related to dopamine through its modulation by other neurotransmitters systems, we addressed the following questions:

(1) Is there space for more dopaminergic strategy beyond dopamine D2R occupancy in psychosis treatment?
(2) If antipsychotics tune the dopaminergic system by mechanisms other than the non-direct D2R-mediated one, what are the other non-canonical dopaminergic targets?
(3) How do antipsychotics acting at D2R impact trans-synaptically other neurotransmitter systems, as well as synaptic and meta-synaptic plasticity?
(4) Finally, how the next generation of antipsychotics now appearing on the landscape of schizophrenia treatment may regulate the dopaminergic system?

With these questions in mind, we have addressed the background and the potential relevance for the therapy of non-canonical molecular effects of antipsychotics considering the complexity of antipsychotics’ multiple receptors profile in the framework of a dopaminergic MOA within and beyond dopamine D2R occupancy.

## 2. Materials and Methods

The bibliographic search and selection process was conducted according to the Preferred Reporting Items for Systematic Reviews and Meta-Analyses (PRISMA) guidelines [15]. The search was carried out on three different databases (EMBASE, Scopus, and PubMed) on 9 December 2022 and was updated on 18 January 2023, before the final writing of the manuscript. It was conducted using the following search string: (((((((((((((Dopamine[Title/Abstract]) OR (dopamine transporter[Title/Abstract])) OR (tryosine 3-monooxygenase[Title/Abstract])) OR (dopamine receptor*[Title/Abstract])) OR (dopa decarboxylase[Title/Abstract])) AND (mechanism of action[Title/Abstract])))) OR (antipsychotic* agent*[Title/Abstract])) AND (postsynaptic density[Title/Abstract])) OR (postsynaptic density protein*[Title/Abstract])) OR (presynaptic terminal*[Title/Abstract])) AND (schizophrenia[Title/Abstract])) OR (treatment-resistant schizophrenia[Title/Abstract]), and retrieved records and full texts were managed by using Endnote X9 according to the Preferred Reporting Items for Systematic Reviews and Meta-Analyses (PRISMA) guidelines [15]. Additional documents were obtained by hand-searching the reference lists of enclosed items with a focus on antipsychotics’ unconventional MOA and possible druggable targets as a therapeutic strategy in schizophrenia and treatment-resistant schizophrenia (TRS). In addition, after reading the entire article, other cross-references have been included. Although this second typology of the search may introduce some differences in the methodology of PRISMA, we believe that the overall strategy used in this translation review linking preclinical and clinical data is the best fit possible to further investigate the canonical and non-canonical antipsychotics MOA in schizophrenia treatment that could be overlooked with a strict systematic search. We considered eligible for the study: English-written in vitro or in vivo, both animal models or human studies, published in peer-reviewed journals that focused on the canonical and non-canonical effects of antipsychotics and putative MOA relevant for schizophrenia treatment and treatment resistance. Furthermore, no time restrictions were enforced, and original clinical and preclinical studies and reviews were included. Conference abstracts and commentaries were excluded. The review protocol is now available on the International Platform of Registered Systematic Review and Meta-analysis Protocols (INPLASY202310079).

## 3. Results

**Figure 1:**
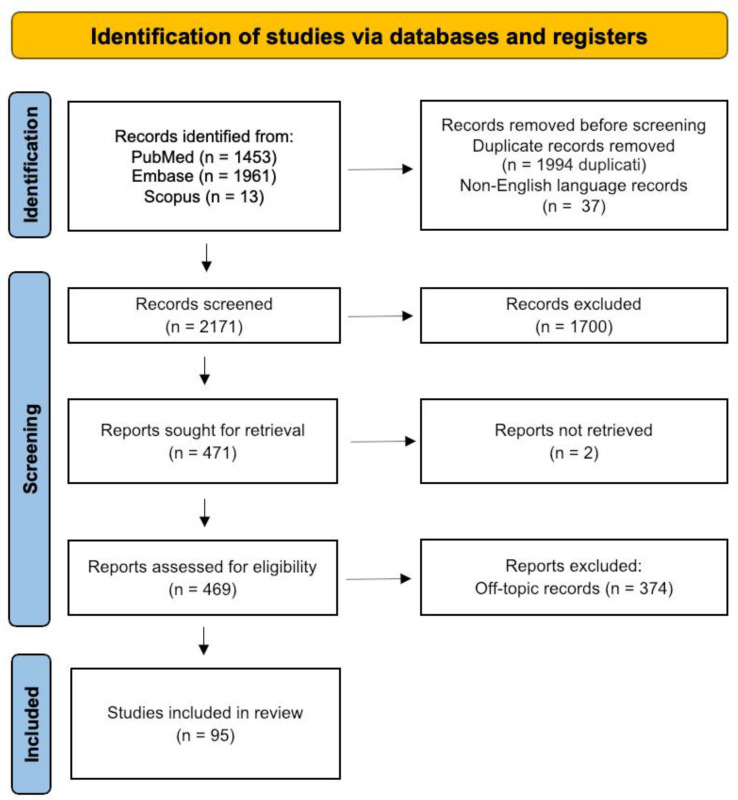
Preferred Reporting Items for Systematic Reviews and Meta-Analyses (PRISMA) flow chart [15].

The PRISMA flow chart has been reported in Figure 1. The search returned a total of 3427. The papers were first evaluated by title/abstract by two independent researchers (LV and MC), and then relevant articles were selected for full-text evaluation based on the eligibility criteria. Inconsistencies were resolved by consensus in a meeting with another researcher (GDS), and the final critical assessment of relevant articles was performed by a second consensus meeting with a board review including all of the authors. Finally, 95 articles were included in the qualitative synthesis. In the following sections, we performed a critical appraisal of the results by analyzing the potentially significant value as a non-canonical (i.e., non-conventional) MOA of antipsychotics in the framework of the dopaminergic system.

### 3.1. Dopamine Receptors and Transporters: Canonical and Non-Canonical Mechanism of Action Relevant for Schizophrenia and Treatment-Resistant Schizophrenia

#### 3.1.1. Dopamine D2 Receptors: Relevance for Treatment-Resistant Schizophrenia and Putative Link with Dopamine Supersensitivity Psychosis

Dopamine dichotomic cortical–subcortical involvement in schizophrenia pathophysiology, may suggest the coexistence of cortical “hypodopaminergia” and subcortical “hyperdopaminergia” associated with the dysfunction of other neurotransmitter systems, mainly serotonergic and glutamatergic ones [16,17]. Treatment with antipsychotics occupying mainly, if not exclusively, the D2R, may counterbalance the hyperdopaminergic state, especially at the striatal level, which is considered the rationale behind antipsychotic action. However, the blockade of D2Rs in the mesocortical, nigrostriatal, and tuberoinfundibular pathways has also been linked to several side effects, such as worsening of cognitive and negative symptoms, extrapyramidal symptoms (EPS), and hyperprolactinemia [18]. Furthermore, it has been proposed that the sustained occupancy of D2Rs could potentially be linked to antipsychotic tolerance, dopamine supersensitivity psychosis (DSP), and tardive dyskinesia (TD) [19]. The mechanisms underlying DSP and TD have been associated with long-term blockade of D2R, leading to upregulation of the receptors, an increase in D2R density, and/or a shift from a “low affinity” to a “high affinity” state [20,21], potentially resulting in “dopamine supersensitivity”.

Iyo and coworkers hypothesized that there is an optimal range in the number of D2Rs available for dopamine binding to achieve adequate treatment in patients with schizophrenia [20]. The authors estimated the optimal D2R occupancy by assuming that the plasma range of antipsychotics was constant while the D2R density varied. The results showed that the plasma level of antipsychotics increased and decreased along with D2R density, estimating that the optimal D2R occupancy range was 65% to 78%, rising to a range of 82% to 89% with increased receptor density, and 42% to 63% with decreased receptor density [20]. The results also indicated that the reduction in the plasma level of antipsychotics was greater in patients with higher D2R density, as they needed increased doses of antipsychotics to achieve optimal receptor occupancy. On the other hand, in patients with lower D2R density, a reduced dose of antipsychotic could achieve optimal receptor occupancy, resulting in liability for extrapyramidal symptoms (EPS) [20]. Kruyer and coauthors have recently demonstrated that behavioral supersensitivity could be related to long-lasting synaptic plasticity processes at pre-, peri-, and postsynaptic sites, which includes the insertion of Ca^2+^-permeable α-ammino-3-idrossi-5-metil-4-isossazol-propionic acid receptors (AMPARs) and the loss of inhibitory postsynaptic currents (IPSCs) dependent on D2R in the middle spiny neurons of the *nucleus accumbens* (NAc) [22]. These findings underlined hyperexcitability causing locomotor sensitization associated with behavioral endophenotypes of resistance to antipsychotic treatment. In this context, the chemogenetic restoration of inhibitory postsynaptic currents in D2R could prevent antipsychotic-induced supersensitivity [22]. Furthermore, an inflammatory-based mechanism implicated in DSP development could be related to oxidative stress resulting from free radicals released by dopamine metabolism. Indeed, dopaminergic neurons in the *substantia nigra* are sensitive to reactive oxygen species (ROS) and to the dysregulation of cellular redox homeostasis generated by catecholamine metabolism [23], which is possibly relevant for TD. There is evidence of increased oxidative stress caused by lipid peroxidation associated with neuronal damage in patients with TRS compared to those responsive ones, potentially significant for DSP and TD [24]. Preclinical studies in rats have shown that neuroleptic-induced TD is also associated with changes in the expression of serotonin 5-HT_2_ receptors (5-HT_2_Rs) [25]. Antipsychotic treatment may reduce the density of 5-HT_2A_Rs in the prelimbic cortex and NAc while increasing their density in the caudate-putamen. Therefore, 5-HT_2A_R activation is necessary for the full expression of DSP induced by antipsychotics, modulating dopamine-dependent behavior. These effects are potentially related to changes in 5-HT_2A_R density in the prefrontal cortex (PFC) and striatum, suggesting the role of the 5-HT_2A_R blockade in the regulation of antipsychotic-induced DSP. Moreover, a mechanistic link has been proposed between the development of TD and serotonin 5-HT_6_R in patients with Parkinson’s disease and transplanted with dopaminergic neurons. The 5-HT_6_R could be implicated in the dopamine release responsible for the “transplant-induced dyskinesia” side effect induced by the inhibition of autoreceptor feedback [26]. For selective high-affinity D2R antagonism, both clinical and preclinical studies have found an association between poor cognitive performance mediated by the disruption of D2R-mediated signaling [27], particularly in the PFC [28]. D2R activation has also been shown to be relevant to dopamine uptake by vesicular monoamine transporter-2 (VMAT-2) [29], suggesting that its pharmacological modulation may be a non-canonical therapeutic target in the treatment of schizophrenia [30]. Despite the elusive neurobiology of DSP, few lines of evidence may suggest a link with TRS [31]. Pharmacogenetics studies have demonstrated a significant relationship between the *DRD2* gene polymorphism -141 C Ins/Del and poor response to antipsychotics [32,33], suggesting the contribution of D2R in the development of DSP and TRS [31,34].

At the molecular level, the mechanisms responsible for D2R trafficking could contribute to DSP [35]. β-arrestin 2 (ARRB2) is directly responsible for internalizing D2R phosphorylated by G protein-coupled receptor kinase 6 (GRK6) and may activate the cellular signaling mediated by the AKT/PP2A/ARRB2 complex, resulting in delayed signaling compared with early G-protein-mediated signaling [36,37]. In addition, the absence of ARRB2 promoted the early G protein pathway by disrupting delayed G-protein-independent signaling [36,37]. Furthermore, D2R partial agonist antipsychotics are responsible for counterbalancing phencyclidine (PCP)-induced hyperlocomotion in wild-type mice, but this effect is lacking in *ARRB2*-knock out (KO) mice [38], indicating a crucial role of ARRB2 signaling in D2R-mediated antipsychotic effects [38]. PCP is involved in developing psychotic and negative symptoms of schizophrenia in healthy individuals [39,40,41,42] and in the exacerbation of these symptoms in schizophrenic patients [43,44]. It has been suggested that GRK6 and ARRB2 might regulate D2R in a low-affinity state for dopamine [45], whereas the GRK6/ARRB2 system was found altered and unable to internalize D2R in the rat DSP model, suggesting that alteration of the GRK6/ARRB2 system has an impact on the pathogenesis of DSP and treatment resistance [34,35].

There is growing evidence that antipsychotics could act as chaperones in the trafficking of dopamine receptors from the intracellular site to the membrane surface; however, not all antipsychotics are equal regarding this feature. Aripiprazole and clozapine have less propensity to act as chaperones of D2R, preventing their translocation and possible upregulation to the cell surface, therefore, this differential mechanism may represent a putative non-canonical MOA to reduce the onset of DSP [46,47].

#### 3.1.2. The “Other” Dopamine D2-like Receptors, Dopamine Non-D2 Receptors and Their Non-Canonical Position in Antipsychotics’ Mechanism of Action

##### The Dopamine D3R

Even though all antipsychotics occupy both D2R and D3R, the role of the latter one has received less attention in the past, probably for the reported low density and more restricted distribution in a few brain regions. However, more recently, a growing interest at both clinical and preclinical levels has considerably changed the appraisal of the D3R in the framework of antipsychotic therapy. At the clinical level, the introduction of cariprazine has significantly marked the role of dopamine D3R as a relevant addition to the schizophrenia pharmacological armamentarium. Cariprazine is the first available antipsychotic that preferentially acts as a partial agonist at D3R, with K_i_ = 0.085 nM, higher than the one for the D2R (K_i_ = 0.49 nM) [48], demonstrating significantly better efficacy on prevalent negative symptoms in schizophrenia patients compared to a full D2R antagonist, such as risperidone [49,50]. At the preclinical level, the D3R occupancy could be implicated in antipsychotic and pro-cognitive effects [51,52,53] by increasing acetylcholine release in the PFC by disinhibiting the activity of dopaminergic neurons projecting to the NAc or PFC, or by activating cAMP response element-binding protein (CREB) signaling in the hippocampus [54]. Of interest, the genetic disruption of the dysbindin gene (*DTNBP1* gene), one of the top candidate genes associated with schizophrenia, affects the intracellular trafficking of D3R. Notably, in both schizophrenia and genetic animal model of the disorder, the concomitant reduction in D3R and *DTNBP1* gene expression was associated with pro-cognitive effects. The D3R/dysbindin interaction has been shown to promote D2R/D3R imbalance by favoring an increase in D2R signaling in the PFC but not in the striatum [55], underlining the potential role of regional D2R/D3R reciprocal ratio of occupancy and D3R antagonism in improving cognitive symptoms in schizophrenia. D3R signaling is emerging as a possible regulatory mechanism of parvalbumin neuron-dependent gamma oscillations (γ-oscillations are believed to be a major organizer of brain functional networking [56] and have been demonstrated to be abnormal in schizophrenia patients [57]). γ-oscillations are 30-90 Hz bursts mainly generated in the cortical–subcortical brain regions, mainly by parvalbumin-positive γ-aminobutyric acid (GABA) interneurons, on the other side, have been demonstrated to be abnormal in post-mortem studies on schizophrenia patients, and animal modeling γ-oscillations have been shown to be deranged by N-methyl-D-aspartate receptor (NMDAR) blockade [58].

##### The Dopamine D4R

D4Rs are predominately located in the prefrontal and temporal areas, sparing the basal ganglia, whereby compounds with a higher affinity for D4Rs than D2Rs could selectively reduce dopaminergic tone in the mesolimbic and mesocortical systems without affecting the nigrostriatal pathway and not producing motor side effects [59]. Furthermore, as D4Rs localized on both pyramidal and GABAergic neurons in the cortex, hippocampus, thalamus, *globus pallidus*, and *substantia nigra* [60], it has been hypothesized that selective D4R antagonists could, directly and indirectly, modulate glutamatergic transmission [60]. D4R seems to also be involved in the restoration of γ-oscillation in the hippocampal slices of aged mice brains, probably with a therapeutic effect on cognitive impairment related to ageing [61]. This effect is reported in hippocampal parvalbumin-positive GABA interneurons and parvalbumin-positive basket cells, which are critical for γ-oscillations, by double *in situ* hybridization and immunofluorescence histochemistry, confirming the crucial role of D4R in cognitive deficits [62].

A potential difference between atypical and typical antipsychotics could concern binding to the D4R, considering that many atypical antipsychotics show a slightly higher affinity for D4R than D2R, whereas the majority of typical antipsychotics have a higher affinity for D2R than D4R [63]. Despite this observation, the affinity for D4R used as a single measure cannot distinguish between typical and atypical antipsychotic drugs [63]. It is possible that antagonism at D4R and 5-HT_2A_ produces discriminative stimulus effects of “atypicality” similar to clozapine, but the additional antagonism at D2R makes it similar to typical antipsychotics [64]. It is noteworthy that the antipsychotic clozapine may interact with D4R concerning its tolerability profile and adverse effects. Indeed, the 120 bp duplication in the *DRD4* gene is significantly associated with clozapine-induced sialorrhea, suggesting that this duplication may increase the risk of sialorrhea in TRS patients treated with clozapine [65].

##### The Dopamine D1R

Dopamine D1R and related intracellular signaling have been longly considered a complex puzzle in the dynamic of dopamine under antipsychotic treatment [66]. One major issue is that the increased release of dopamine in schizophrenia is supposed to hit both classes of dopamine receptors, D1- and D2-like, and on the other side, all antipsychotics, with few exceptions (asenapine and clozapine), have relatively low affinity for D1R compared to D2R affinity and occupancy/antagonism. Therefore, D1R remains, except for asenapine and clozapine, a “black spot” in the dopaminergic-related effects of antipsychotics in terms of efficacy. Despite the low D1R affinity of antipsychotics, recently, some non-canonical mechanisms have emerged supporting an indirect role of D1R in antipsychotics action among, for example, chlorpromazine, a D1R-like inverse agonist in the mouse brain, which reduces Ca_v2.2_ currents by occluding D1R-like constitutive activity [67,68]. Moreover, the evidence that D1R- NMDAR reciprocal interaction, especially in the NAc, is involved in behavioral preclinically relevant for schizophrenia modeling prepulse inhibition (PPI) [69], suggests the possibility that D1R may be manipulated by acting on NMDAR. Lynch and co-workers proposed that dopamine hypoactivity in the NAc could be behind the negative symptoms of schizophrenia and that treatments with low-dose dopamine agonists could mimic the behavioral profile of negative symptoms via direct stimulation of D1Rs in the PFC [70]. Accordingly, clozapine’s efficacy for negative symptoms could be partially ascribed to the blockade of D1Rs in the PFC, resulting in enhanced dopaminergic activity in the NAc favored by glutamate [70]. Indeed, binding assays showed that clozapine has a higher affinity for D1Rs than D2Rs [71], acting preferentially on D1Rs localized in the frontal cortex instead of in the striatum, hypothesizing a regional selectivity based on its receptor peculiarity [72].

Preclinical studies reported that clozapine suppressed the acute increase in medial PFC glutamate levels by enhancing NMDAR-mediated neurotransmission that was not induced by D1R-dependent dopaminergic neurotransmission, partially explaining the clozapine-induced attenuation of hyperlocomotion in PCP-treated rats [73].

**Figure 2:**
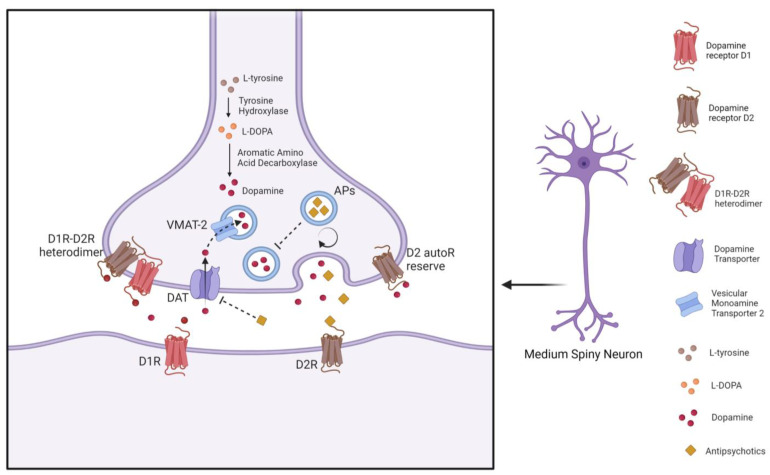
Presynaptic dopaminergic terminals and dopamine transporter in schizophrenia. The co-expression of D1R-D2R heterodimers at the presynaptic terminals in medium spiny neurons regulating calcium/calmodulin kinase II signaling directly in the *nucleus accumbens* [76]. The antipsychotic mechanism of action involving the presynaptic dopaminergic terminal. In this regard, preclinical studies showed that antipsychotics could accumulate in the synaptic vesicles resulting in a significant release of extracellular drug concentrations that inhibit presynaptic function [78,79]. Treatment with antipsychotics could generate an intracellular drug store co-released by vesicles together with endogenous dopamine, resulting in the inhibition of presynaptic voltage-gated sodium channels, which regulate dopamine release with an autoinhibitory effect. The formation of a ‘reserve’ of presynaptic D2 autoreceptors that interact mainly with endogenous dopamine, whereas postsynaptic D2Rs could be mostly occupied by antipsychotics. The blockade of DAT has been responsible for the restoration of synaptic dopamine levels in chronic treatment with antipsychotics. Dopamine uptake by VMAT-2 showed to be relevant to D2R activation, suggesting its pharmacological modulation as a therapeutic target in schizophrenia. L-DOPA, Levodopa; D1R, Dopamine receptor D1; D2R, Dopamine receptor D2; DAT, Dopamine transporter; VMAT-2, Vesicular monoamine transporter 2; APs, Antipsychotics; D2 autoR, Dopamine D2 autoreceptor. Created with BioRender.com on 7 March 2023.

However, the results of clinical trials with selective D1R antagonists do not show antipsychotic responses in patients [74]. A different look at D1R in terms of antipsychotic response comes from the co-expression of D1R with D2R, which has been shown to increase the affinity of clozapine for D1R [75]. A variable distribution of medium spiny neurons co-expressing D1R and D2R in the basal ganglia has been reported, with the highest incidence in the NAc and *globus pallidus* and the lowest incidence in the caudate putamen [76]. It has been hypothesized that D1R-D2R heterodimers located in cell bodies and presynaptic terminals promote grooming behavior and attenuate α-Amino-3-Idrossi-5-Metil-4-isoxazolone propionate receptor (AMPAR) GluR1 phosphorylation by regulating calcium/calmodulin kinase II (CAMKII) signaling directly in the NAc [76]. D1R-D2R heterodimer formation and functional activation in the *globus pallidus* are increased in schizophrenia [76,77] and are at least in part supported by the increase in the affinity of clozapine for D1R in the heterodimers [75] (Figure 2). Further research on the putative role of D1R/D2R heterodimers and potential exploitation for bypassing the poor response to conventional antipsychotics in TRS is warranted.

#### 3.1.3. The Role of Presynaptic Dopaminergic Terminals and Dopamine Transporter

Clinical studies have estimated that the time course of D2R occupancy in the living human brain achieves adequate central D2R blockade within hours of antipsychotic administration, whereas the appreciable antipsychotic effect appears after days or weeks of treatment [80]. However, the delayed clinical antipsychotic effect can be explained both by the drug’s action at postsynaptic D2R and its MOA at the presynaptic dopaminergic terminal. In this regard, preclinical studies showed that weak-base antipsychotics (e.g., haloperidol, chlorpromazine, clozapine, and risperidone) could accumulate in the synaptic vesicles of presynaptic dopaminergic terminals, resulting in a significant release of extracellular drug concentrations that inhibit presynaptic function in an activity-dependent manner [78,81] (Figure 2). In this context, it has been proposed that released levels of antipsychotics may recycle with synaptic vesicles by exerting an autoinhibitory effect on vesicular exocytosis, promoted by inhibition of voltage-gated sodium channels (VGSCs) and dependent on the intensity of stimulation, regulating synaptic transmission [78]. Therefore, treatment with antipsychotics could generate an intracellular drug store co-released by vesicles together with endogenous dopamine, resulting in the inhibition of presynaptic VGSCs, which in turn regulate dopamine release with an autoinhibitory effect [78]. Another antipsychotic MOA hypothesized the formation of a “reserve” of presynaptic D2R autoreceptors while postsynaptic D2Rs would be instead occupied by antipsychotics. Endogenous dopamine would thereby mainly interact with D2 autoreceptors, reducing the presynaptic synthesis and release of dopamine and leading to an indirect antipsychotic effect [5]. However, it has been shown that the initial increase in synaptic dopamine availability after exposure to antipsychotics may decrease over time by reducing dopamine levels during chronic treatment resulting in a loss of antipsychotic efficacy [5]. Therefore, the reasons for drug tolerance or treatment failure could lie in the reduced dopamine levels at dopaminergic synapses and the consequent lack of stimulation of the presynaptic D2R reserve. Furthermore, it has been proposed that restoration of initial synaptic dopamine levels may improve antipsychotic efficacy in chronic treatment, suggesting the blockade of dopamine transporter (DAT) as a therapeutic non-conventional option [5]. This evidence extends the canonical view of antipsychotics acting at postsynaptic receptors to the non-canonical action at the presynaptic dopaminergic terminal, either through inhibition of VGSCs or through the indirect stimulation of the D2 autoreceptor reserve. This insight may be relevant to elucidate the complex changes induced by antipsychotics within synapses but also shed light on DAT blockade as an additional treatment to revert antipsychotic treatment failure, such as in the TRS condition (Figure 2).

### 3.2. Dopaminergic Mechanisms and Correlations with Antipsychotics Receptor Profile: Insights from Present and Next Molecules

Antipsychotics have, with some exceptions (i.e., amisulpride and other benzamides), a complex receptor profile even if dopamine D2R occupancy or blockade is considered to be the necessary and possible sufficient mechanism of antipsychotic action. Exploring which and to what extent receptors that are part of an antipsychotic’s profile are different from dopamine receptors could cooperate with the latter ones to help to unveil potential new antipsychotic mechanisms of non-canonical type.

#### 3.2.1. Dopamine and Serotonin Receptors

The possibility that non-canonical mechanisms in dopaminergic signaling could intercept serotoninergic signaling has been recently challenged by the discovery of receptor dimers belonging to the different classes of neurotransmitters [82,83,84].

A trait that has been considered possibly critical for the atypicality of almost all new-generation antipsychotics is 5-HT_2A_ antagonism; specifically, a better affinity for 5-HT_2A_ over D2R, and thus a high 5-HT_2A_/D2R ratio is considered a possible molecular predictor of atypicality, whose clinically and original definition is the low liability to elicit EPS [82]. In the framework of dopamine–serotonin receptor “cooperation” and with respect to the possible non-canonical dopaminergic MOA of antipsychotic drugs, an emergent role has been attributed to the 5-HT_2A_/D2R dimers. Several preclinical in vitro and in vivo studies have identified dimerization of 5-HT_2_ with D2R in rat striatum [82,83,84]. Specifically, it showed that stimulation of 5-HT_2A_/D2R dimers with D2R agonists is antagonized by co-administration of 5-HT_2A_-agonists, suggesting a 5-HT_2A_-mediated trans-inhibition of D2Rs, resulting in increased G_q_ compared with G_i_ signaling [85]. Furthermore, in vitro and in vivo studies have shown that 5-HT_2A_ can form dimers with metabotropic glutamate receptor 2 (mGluR2) [86,87]. In this context, 5-HT_2A_/mGluR2 heterodimerization could potentiate mGluR2-G_i_ signaling and inhibit 5-HT_2A_-G_q_ one [88]. In this context, serotonergic and glutamatergic drugs showed to bind the 5-HT_2A_/mGluR2 heterocomplex, balancing G_i_- and G_q_-dependent signaling and eliciting antipsychotic action [88]. In addition, post-mortem studies in the cortex of untreated schizophrenia patients found higher expression of 5-HT_2A_ and lower expression of mGluR2, which might reflect a predisposing pattern to psychosis, indicating that the 5-HT_2A_/mGluR2 complex might be involved in the altered cortical processes of schizophrenia resulting in a promising unconventional target for the treatment of psychosis [86]. Several lines of evidence indicated that 5-HT_3_ is involved in the modulation of dopaminergic activity in mesolimbic and nigrostriatal pathways [89,90,91], suggesting the role of 5-HT_3_ antagonists in mimicking the effects of antipsychotic drugs. Clinical evidence on single-marker and haplotype analyses among different mutations of serotonin receptor subtypes (*HTR2A*, *HTR3A*, and *HTR4*) in patients with TRS found that the daily dosage of neuroleptics received during maintenance therapy was significantly higher in patients with the T/T genotype of the *HTR3A* rs1062613 polymorphism, supporting the relationship between the *HTR3A* polymorphism and the potential development of TRS [92]. 5-HT_3_ antagonism is yet to be clarified [93]; also 5-HT_6_ antagonism was shown to attenuate the pro-psychotic effects of both MK-801 (also known as dizocilpine hydrogen maleate, a non-competitive antagonist at NMDAR) [94] and PCP in animal models of schizophrenia [95,96]. Furthermore, the selective 5-HT_7_ receptor antagonist SB-269970 improves ketamine-induced attention and cognitive inflexibility, although its role has not yet been elucidated [97]. Partial 5-HT_1A_ agonists are known to have effects shared in part by 5-HT_2A_ antagonists in different biological systems [98] with anxiolytic and antidepressant efficacy [99,100]. Moreover, it has been suggested that 5-HT_1A_ agonism contributes to the atypical profile of antipsychotics [101], reducing movement disorders liability [102,103] and improving cognitive and affective symptoms [104,105]. In addition, association studies between genetic variants of the *HTR1A*, as well as solute carrier family 6, member 4 (*SLC6A4*) genes and clinical outcomes in schizophrenia, have shown that rs6295 and 5-HTTLPR polymorphisms significantly influence clinical symptoms in schizophrenia [106]. Finally, the involvement of 5HT_1A_ in schizophrenia pathophysiology is also confirmed by an in vitro preclinical study in PC12 cells treated with clozapine: it is demonstrated a decrease in tyrosine hydroxylase levels, with subsequent reduction in dopamine levels, probably mediated by D2R and 5HT_1A_ stimulation [107].

#### 3.2.2. GABA Receptors

The main inhibitory neurotransmitter in the human central nervous system (CNS) is GABA. In postnatal development, GABAergic neurons are instrumental to the formation of brain circuits and are also involved in the pathophysiology of schizophrenia [108]. Animal models in which rat were treated with MK-801 exhibited a reduction in the density of parvalbumin-immunoreactive GABAergic neurons in the mPFC [109], partially resembling some findings of schizophrenia post-mortem brains [110]. Several abnormalities in GABA neurotransmission have been reported in patients with schizophrenia and TRS, such as morphological alterations in cortical and hippocampal GABA interneurons [108], reductions in cerebrospinal fluid (CSF) GABA levels in first-episode psychosis (FEP) [111], decreased glutamic acid decarboxylase (GAD67) levels in the dorsolateral PFC (DLPFC) [112,113], reduction in GABA_B_ receptors expression in the post-mortem PFC and hippocampus [114,115], and genetic polymorphisms of *GAD67*, *GABBR1*, and *GABBR2* genes, which encode for the GABA_B_ receptor [116]. An impaired ability to filter external auditory sensory information mediated by altered GABA_B_ receptor firing has been associated with schizophrenia [117,118]. Specifically, an increase in the cortical silent period directly correlated with GABA function was observed in clozapine-treated patients compared to other antipsychotics [119,120], suggesting clozapine’s ability to improve signal-to-noise discrimination [121], potentially through the potentiation of GABA_B_-mediated inhibitory transmission [119,122]. Through X-ray crystal structure analysis, a recent study demonstrated that clozapine binds directly to the GABA_B_ similarly to baclofen, a GABA_B_ receptor agonist proposed as a non-canonical antipsychotic [122]. Furthermore, a proton magnetic resonance imaging (^1^H-MRS) study measured GABA levels in the midcingulate cortex (MCC) of TRS patients, stratifying them into clozapine-resistant schizophrenia (CRS) and TRS patients. The results showed higher GABA levels in CRS compared to TRS patients suggesting the potential implication of GABA to clozapine resistance [123]. Conversely, a recent meta-analysis reported overall decreased GABA levels in the MCC of FEP patients compared to healthy control (HC) and elevated levels of glutamate and glutamate + glutamine (Glx, glutamate neurotransmitter and its precursor) in the same region of TRS patients suggesting a disruption of the excitatory/inhibitory balance in schizophrenia spectrum disorders [124]. Abnormalities in Glx levels have been associated with schizophrenia, possibly contributing to the dysfunction of glutamatergic neurotransmission [125,126]. Glutamate is released into the synaptic cleft and binds glutamate receptors at the PSD. Specifically, glutamate is subsequently removed by astrocytes and converted to glutamine by glutamine synthetase [127]. Then, glutamine can be internalized into the presynaptic terminal and converted to glutamate by phosphate-activated glutaminase [128,129]. Intriguingly, Madeira and collaborators suggested that circulating Glx levels exhibit a biphasic pattern in schizophrenia, with an increased glutamine/glutamate ratio at the onset and decreased levels with the progression of the disease [130]. α5-GABAARs have been measured in the hippocampus by means of PET [11C]Ro15-4513, showing lower levels in first-episode schizophrenia patients antipsychotics naïve compared to controls, while no differences are found in patients on antipsychotic treatment compared with controls [131]. Beyond some indirect effects of antipsychotics on GABA neurotransmission, there was a long-standing acknowledgement that antipsychotics do not act directly at GABAergic receptors. This view has been challenged recently by the observation that applying in vivo fiber photometry and chemogenetics antipsychotics (i.e., olanzapine and clozapine) may directly bind and work as antagonist GABA_A_ receptors in ventral tegmental area (VTA). As a consequence, GABA neurons are activated, and the GABAergic projection from VTA to the NAc is involved in such antipsychotic effects [132].

#### 3.2.3. Noradrenergic Receptors

Alpha-adrenoceptor 1 (α_1_) and alpha-adrenoceptor 2 (α_2_) antagonism are both considered to be involved in modulating dopamine release in the PFC through firing dopaminergic neurons in the VTA [133], as demonstrated by the selective α_1_ antagonist action of prazosin, which is responsible for enhancing the cognitive performance of MK-801 pretreated rats [134]. The available evidence indicates that blocking α_1_ by antipsychotics may help to suppress positive symptoms, particularly in acute schizophrenia, whereas blocking α_2_, a prominent effect of clozapine and, to a lesser extent, risperidone, may instead help to partially relieve negative and cognitive symptoms. While α_1_ blockade may inhibit striatal hyperdopaminergia at the presynaptic level, α_2_ blockade may enhance and improve dopaminergic function in PFC [133]. In fact, non-selective α_2_ blockage has been linked to the effects of atypical antipsychotics such as clozapine and antidepressants such as mirtazapine. It has been suggested that α_2C_ and α_2A_ heteroreceptors can regulate non-noradrenergic transmission, such as serotoninergic and dopaminergic, by sharing autoreceptor functions to inhibit noradrenaline release via negative feedback. In addition, while α_2A_ seems to be highly distributed across the CNS, α_2C_ expression is less abundant, suggesting the differential modulation of regional neurotransmission. α_2C_ antagonism could be helpful during states of low endogenous noradrenergic activity, whereas α_2A_ antagonism could be significant during states of high noradrenergic tone. Specifically, preclinical studies have shown that the antagonism at the α_2C_ represents a non-canonical MOA for the regulation of GABA release in the striatum, inducing inhibition of GABA neurons projecting to cortical pyramidal neurons correlating with antidepressant/antipsychotic and procognitive-like effects [135,136]. In this context, α_2C_ antagonists could unconventionally mitigate schizophrenia-associated symptoms [136], ameliorating cognitive deficits and PCP-induced social interaction impairment [137,138,139]. Furthermore, it has been proposed that clozapine MOA at the α_1_ and α_2_ receptors could globally stabilize dopaminergic transmission [46]. In fact, its α_1_ antagonism could affect positive symptoms by attenuating limbic hyperdopaminergia, whereas α_2_ blockade could act on negative symptoms by increasing prefrontal dopaminergic activity, both contributing to improving secondary negative symptoms [140].

**Figure 3:**
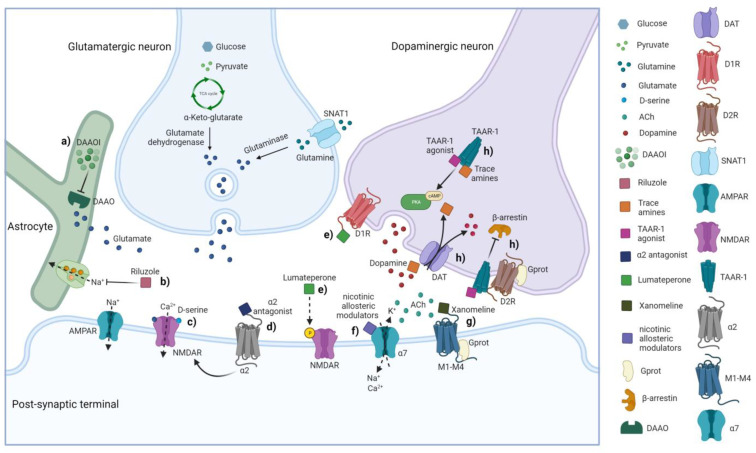
Mechanisms of action of molecules with non-canonical antipsychotic action: (**a**) DAAO inhibitors are capable of inhibiting the DAAO enzyme expressed by astrocytes; (**b**) riluzole reduces the synaptic release of glutamate by inhibiting voltage-gated sodium channels and calcium currents and increases astrocytic reuptake of glutamate enhancing cortical glutamate metabolism [145,146]; (**c**) D-serine acts as a co-agonist at synaptic NMDARs enhancing glutamatergic tone and its levels are reduced by DAAO activity, responsible for D-serine catabolism; (**d**) α2 antagonist enhances both cortical dopamine release and NMDAR-mediated responses; (**e**) lumateperone acts on dopaminergic D1R and D2R stimulating the phosphorylation of NMDAR subunits [147]; (**f**) α7 nicotinic positive allosteric modulators mitigate cognitive symptoms by eliciting acetylcholine release; (**g**) xanomeline is a central selective muscarinic agonist of M1 and M4 demonstrated to have antipsychotic properties despite being devoid of a direct effect on D2R [148]; (**h**) TAAR-1 agonists act on the intracellular TAAR1 receptors expressed by dopaminergic neurons and functionally related to receptors for biogenic amines. DAAO, D-amino acid oxidase; DAAOI, D-amino acid oxidase inhibitor; SNAT1, Sodium coupled neutral amino acid transporter 1; AMPAR, α-amino-3-hydroxy-5-methyl-4-isoxazolepropionic acid receptor; NMDAR, N-methyl-D-aspartate receptor; D1R, Dopamine receptor D1; D2R, Dopamine receptor D2; cAMP, Cyclic adenosine monophosphate; PKA, Protein kinase A; TAAR-1, Trace amine-associated receptors 1; DAT, Dopamine transporter; P, phosphorylation; Ach, Acetylcholine; M1, Muscarinic receptor 1; M4, Muscarinic receptor 4; α2, α2 adrenoceptor; α7, α7 nicotinic receptor; Gprot, G protein; Na^+^, Sodium; Ca^2+^, Calcium; K^+^, Potassium. Created with BioRender.com on 7 March 2023.

Preclinical studies have shown that the antipsychotic effect of risperidone can be potentiated by additional treatment with an α_2_ antagonist, idazoxan, resulting in the attenuation of EPS and the enhancement of cortical dopamine release and NMDAR-mediated responses [141] (Figure 3). Consistent with these findings, the α_2_ antagonism action of clozapine together with low D2R affinity could be considered a combined non-canonical MOA intercepting dopamine function and possibly relevant for its efficacy in TRS [141]. Moreover, the combination of idazoxan and raclopride, a selective D2R/D3R antagonist, was proposed to produce an atypical antipsychotic profile partially overlapping the clozapine MOA [142]. Nevertheless, the co-administration of raclopride and idazoxan was not able to reverse PPI deficit in rat animal model of schizophrenia, challenging the hypothesis that simple α_2_/D2R blockade may elicit atypical antipsychotic activity [142]. Another attempt to combine a D2R antagonism with adrenoreceptor α_2_ antagonism was the combination/augmentation of idazoxan with haloperidol [143] or fluphenazine [144], yielding mixed results. Overall, the combined dopamine D2R and adrenoreceptor α_2_ antagonism could be a non-canonical dopaminergic strategy worth further exploration.

#### 3.2.4. Muscarinic Receptors

The role of acetylcholine is crucial for hippocampal function, including learning and memory processes impaired in schizophrenia, supporting disturbances of cholinergic systems in such disease [149,150]. The excitatory postsynaptic muscarinic receptors 1 (M1) and muscarinic receptors 4 (M4) are the main cholinoceptive targets in the PFC and thus may be involved in both the pathology and pharmacotherapy of schizophrenia [151]. Clinical evidence has shown that subjects with schizophrenia show a selective reduction in the expression of muscarinic receptors (especially M1) in the CNS [149,150,151,152] associated with cognitive impairment. Since M1 are critical for PFC acetylcholine functions [151], it has been suggested that the reversal of M1 expression could be a potential therapeutic target for antipsychotics, as demonstrated in a preclinical study using clozapine metabolite, N- desmethylclozapine, that exhibit an antagonism at M1 [153,154,155]. Preclinical studies hypothesized that positive allosteric modulation of M1 could enhance glutamatergic transmission mediated by the NMDAR in the hippocampus [156]. Moreover, the administration of selective M1 agonists showed to improve long-term depression (LTD), cognitive functions, and social skills in schizophrenia mouse models [157], providing new insights into synaptic alterations that may contribute to behavioral deficits and supporting M1’s role as an unconventional target for the treatment of schizophrenia. In contrast, the function of muscarinic receptor 3 (M3) has not been elucidated in the pathophysiology of schizophrenia other than probably related to the adverse effects of antipsychotics, such as type 2 diabetes [158], considering that M3 mediates insulin release from pancreatic β cells directly through G_q_ protein signaling [159] and indirectly through β-arrestin and polycystin 1 (PKD1) signaling [158]. Of interest, De Luca and collaborators have demonstrated that single-nucleotide polymorphisms (SNPs) of the muscarinic receptors 5 (*M5*) gene are associated with susceptibility to schizophrenia [160]. In addition, preclinical studies in mice with constitutive deletion of the *M5* gene have reported affected sensorimotor gating and PPI mechanisms [161]. Preclinical evidence has reported the involvement of M4 in cognitive functioning [162] and the prevention of hyperexcitability of midbrain dopaminergic neurons, presenting M4 agonists as an unconventional strategy for the treatment of disorders associated with hyperdopaminergia [163]. *Post-mortem* studies in schizophrenia patients have reported low levels of M1, M2, and M4 in the striatum affecting motivation and motor control [152]. Indeed, M1–M4 agonism seems to be an interesting pharmacological mechanism for antipsychotics, as the dopamine-acetylcholine balance is relevant to decrease behavioral disturbances [164,165,166]. In this context, Shannon and coworkers demonstrated, in conditioned avoidance response, that muscarinic receptor agonists decreased the avoidance response in a manner similar to antipsychotics, suggesting that muscarinic receptor agonists may provide a non-canonical approach to the treatment of psychosis [164]. Thus, the muscarinic receptor agonist xanomeline demonstrated antipsychotic properties despite being devoid of a direct effect on D2R [148] but acting as a selective agonist of central muscarinic receptors M4 and M1 [167], proving significant efficacy in reducing positive and negative syndrome scale (PANSS) total score with associated peripheral side effects [152,168,169]. Radioligand binding studies on cloned human receptors confirmed that xanomeline has a substantial affinity for muscarinic receptors [167], as well as for 5-HT_1_ and 5-HT_2_ receptor subtypes [148]. Watson and coworkers have shown xanomeline to be a potent agonist of 5-HT_1A_ and 5-HT_1B_ receptors in native tissues and/or cloned cell lines and an antagonist of 5-HT_2_ receptor subtypes [167], speculating potential antipsychotic activity related to action on these receptors. Despite the effect on M1 and M4 receptors is also shared by the atypical antipsychotic clozapine, Thomsen and coworkers inferred that the effects on PPI through both M1 and M4 in mice do not support the role of these receptors in mediating antipsychotic-like effects of clozapine [166]. The central and peripheral antimuscarinic affinity of antipsychotics is responsible for side effects, such as dizziness, drowsiness, confusion, blurred vision, and others [170,171]. The expedient for limiting peripheral cholinergic effects has been to co-administer xanomeline with trospium chloride, a peripheral muscarinic antagonist unable to cross the blood–brain barrier (BBB). Several muscarinic agonists have been shown to exert a functional antidopaminergic effect, particularly in the VTA, despite their lack of affinity for dopamine receptors [172]. A growing body of preclinical evidence has shown that xanomeline reverses sensory-motor gating deficits but may also improve cognitive dysfunction [173]. Clinical studies have tested the safety and efficacy of xanomeline and trospium as monotherapy in non-TRS patients [168], while the putative role in TRS may be inferred from the use of clozapine, whose metabolite norclozapine acts as an M1 and M4 agonist [46]; thus xanomeline’s non-canonical MOA could be a valuable augmentation strategy to conventional antipsychotics in poorly responsive schizophrenia patients [169,174] (Figure 3).

Of interest, α7 nicotinic receptors reduced levels associated with impaired auditory gating have been reported in the hippocampus of subjects with schizophrenia [175]. In particular, clozapine showed to dose-dependently normalize auditory gating in mice through α7 nicotinic receptors, an effect not shared with haloperidol [176]. While subchronic administration of MK-801 reduced protein and gene expression of α7 nicotinic receptors in the hippocampus, clozapine treatment restored α7 expression and reversed cognitive deficits in male rats [177]. Although typical antipsychotics are associated with cigarette smoking in patients with schizophrenia, clozapine appears to reduce nicotine use [178,179,180], probably due to its peculiar action on nicotinic receptors.

Taking this evidence together, positive allosteric modulators of α7 nicotinic receptors have been proposed as an additional non-canonical strategy to mitigate cognitive symptoms in schizophrenia [177,181,182] (Figure 3). In phase II double-blind Randomized Clinical Trial (RCT), the agonist of the α7 receptor encenicline was tested on cognitive domains as an augmentation strategy and showed clinically significant improvements in schizophrenia patients [183]. Nicotinic agonists are thought to primarily affect cognitive symptoms [184], while muscarinic agonists have been shown to improve positive symptoms [185].

In summary, α7 nicotinic and M1/M4 agonists, as well as positive allosteric modulators in add-on/augmentation to dopamine D2R occupancy, could be considered among promising non-canonical strategies for schizophrenia treatment [168,169,183]. It remains to be understood which antipsychotic with which receptor profile should be considered the best fit for a such combination therapy.

#### 3.2.5. Histamine Receptors

Histamine has a remarkable role as a regulator of several neurotransmitters and critical brain functions [186]. Approximately 64,000 histaminergic neurons in the human brain have their soma in the tuberomamillary nucleus in the posterior hypothalamus and send their axons to several brain areas, including the thalamus, hippocampus, striatum, amygdala, and cortex [186]. Histamine acts through at least four G-protein-coupled receptors (H1–H4), of which H1, H2, and H3 receptors are expressed in the CNS [187]. However, clinical experimental data on the role of histamine in psychosis are scarce, whereas preclinical evidence is challenging. Animal studies have shed on the potential role of histamine system and traditional models of psychosis, as well as interactions between histaminergic drugs and antipsychotics. Indeed, following the stimulation of the D2R, methamphetamine has been shown to increase histamine levels in rat brains, suggesting that blockade of the H3 attenuates the effect of amphetamines on locomotion [188], an effect replicated in *HRH3* (*H3 receptor gene*)-KO mice that exhibited decreased spontaneous locomotion and poorly respond to methamphetamine [189]. In this frame, Pillot and colleagues have suggested that endogenous histamine and dopamine cooperate to modulate the activity of striatal projection neurons fueling interest in the H3 antagonism as an unconventional antipsychotic strategy, possibly when combined with D2R occupancy [190]. While the antagonism of clozapine at the H3 might contribute to its overall clinical efficacy [191,192,193,194], H4 agonism seems to be related to severe adverse events, such as agranulocytosis [195]. In addition, both H3 and H4 may mediate multiple interactions between neurotransmitter systems involved in modulating appetite, satiety, and food intake relevant to clozapine’s cardiometabolic side effects [196,197]. In vivo functional PET neuroimaging studies showed that schizophrenia patients had a lower binding affinity for the H1 in the frontal cortex, PFC, and cingulate gyrus compared with HC [198]. Furthermore, in post-mortem studies, H1 were reduced in the frontal cortex of chronic schizophrenia patients [199]. However, the binding of the antipsychotic clozapine, a potent H1 antagonist [71,200], is believed to be responsible for several side effects, including weight gain, sedation, and orthostatic hypotension [201,202,203,204,205]. In addition, it was found that the H1 antagonism of several antipsychotics could expose schizophrenia patients to the risk of ischemia [206]. H1 blockade could be implicated in the enhancement of sensorimotor plasticity and memory functions, suggesting that H1 antagonism may result in antipsychotic action [207]. A comparative in vitro and in vivo re-evaluation study showed that clozapine is a complete H2 inverse agonist and that its repeated administration increases H2 regulation in rat brain, suggesting that clozapine’s histaminergic affinity might partially account for its atypical profile, as well as with that related to central and peripheral side effects [197].

Furthermore, a double-blind, parallel-group RCT study evaluated the potential beneficial effect of the H2 antagonist famotidine on negative symptoms in TRS patients, suggesting the novel pharmacological potential of H2 antagonism as a non-canonical strategy to treat negative symptoms in TRS [208].

#### 3.2.6. TAAR1

Among non-canonical mechanisms intercepting dopaminergic signaling in treating schizophrenia, trace amines and their receptor have emerged strongly. Endogenous trace amines, which structurally resemble monoamines, such as serotonin and dopamine, activate the trace amine-associated receptor 1 (TAAR1) [209]. Since trace amines have been shown to alter the release and/or response to dopamine, norepinephrine, acetylcholine, and GABA [210], they are thought to be potential neuromodulators. When TAAR1 and D2R interact, a reduction in β-arrestin 2 recruitment is detected, silencing the GSK3 cascade via Akt [211,212]. Preclinical research suggests that TAAR1 agonists may improve not only the behaviors proxy of positive symptoms but the one mimicking negative and cognitive symptoms too, without causing motor disorders or weight gain [213]. In order to be considered a multimodal therapeutic target for neuropsychiatric diseases, the role of TAAR1 as a critical node in the regulation of dopaminergic signaling has been established through a combination of experimental preclinical and translational studies [212] (Figure 3). More attention will be paid to dopamine, glutamate, and serotonin regulation by TAAR1 as a non-canonical mechanism of antipsychotic efficacy [212]. SEP-363856, a novel therapy approved by the Food and Drug Administration for the treatment of schizophrenia, is a TAAR1 agonist in phase III clinical development. Since this compound modulates dopamine release without directly interacting with presynaptic and/or postsynaptic D2R, it may be able to treat TRS when other antipsychotics have failed and/or DSP has developed [174]. Specifically, SEP-363856 reduced the PANSS total score significantly more than the placebo and standard treatments in patients with acute schizophrenia [214], also improving the safety profile [215].

Furthermore, it should be noted that this molecule acts primarily at the presynaptic level, which is believed to be the pivotal site of dopaminergic dysregulation [174].

Consistent with these results and TAAR1 agonists’ unique MOA, the beneficial effect of these compounds as non-canonical therapy for TRS can be assumed [174].

#### 3.2.7. Dopamine-Antagonism and Glutamate-Based Augmentation of Antipsychotics

There is an emerging interest in dissecting non-canonical mechanisms that point to glutamate involvement as an antipsychotic augmentation strategy. The NMDAR hypofunction hypothesis predicts a reduced function of parvalbumin-positive fast-spiking GABA interneurons considered a major determinant of schizophrenia molecular pathophysiology with a possible role in poor response to antipsychotics acting at D2R [216]. NMDAR function can be improved by increasing D-amino acids by D-amino acid oxidase inhibitor (DAAOI), sodium benzoate, which is used as a therapy to target residual positive symptoms and TRS [210].

Encouraging clinical studies have shown that the strategy of clozapine augmentation with sodium benzoate, which is expected to promote the activation of NMDAR, exhibits beneficial effects overall in patients with CRS [217] and improves cognitive functions in patients with chronic schizophrenia [218]. Further research is needed to understand whether sodium benzoate, as an add-on to clozapine, can support efficacy, tolerability, and potential unconventional use for TRS and CRS [217]. Some evidence suggests low bioavailability and poor capability across the BBB, limiting the use of DAAOIs [219]. The efficacy results are promising, and sodium benzoate may be an unconventional option for difficult-to-treat patients, but pharmacokinetic issues need to be better addressed.

Modulators of glutamatergic neurotransmission have been tested in several studies in combination with clozapine. However, no cumulative evidence of significant clinically beneficial differences was shown, except for improvement in negative symptoms with memantine and minocycline [220]. It is possible that the use of additional glutamatergic agents in patients receiving clozapine may have little effect, possibly due to a putative ceiling effect on the NMDAR transmission enhancement already exerted by clozapine [221]. Strategies based on the potentiation of glutamatergic signaling by glycinergic agents (e.g., glycine and D-serine), glycine transporter 1 (GlyT1) inhibitors (e.g., bitopertin and BI 425809), and other glutamate allosteric NMDAR modulators (e.g., CNS4) have been tested. D-serine has been assessed in several RCTs in augmentation to clozapine or other antipsychotics, showing poor beneficial effects among patients treated with clozapine, whereas a meta-analytic study indicated that adjunctive treatment with D-serine and glycine was effective with other antipsychotics in improving the negative symptoms of schizophrenia [222] (Figure 3). A preclinical in vivo electrophysiological study reported the changes in endogenous concentration of brain kynurenic acid (KYNA) used to analyze the interaction between clozapine and the glycine site of NMDAR. KYNA is an endogenous blocker of α7 nicotinic receptors and a glutamate-receptor antagonist, preferentially blocking NMDAR. The results showed that the endogenous levels of brain KYNA were crucial for the response to clozapine on the VTA dopamine neurons, suggesting the ability of clozapine to interact with glutamatergic mechanisms via NMDA/glycine receptors [223].

An increase in glycine availability may also be obtained by administering GlyT1 inhibitors, which have proven to modulate both glutamatergic and dopaminergic neurotransmission in an animal model of schizophrenia [224]. Bitopertin showed no significant effects on overall symptoms in schizophrenia patients [225]. Encouraging evidence indicates the putative efficacy of another non-sarcosine GlyT-1 inhibitor, BI 425809, recently evaluated in a 12-week, double-blind, phase II RCT study associated with cognitive improvement in schizophrenia patients [226].

Experimental strategies to add D-amino acids to antipsychotic drugs to enhance response to treatment in schizophrenia have been investigated for several years, but despite a sufficiently strong rationale and translational background [221], it is still missing to take advantage of the availability of D-amino acids used in the clinical setting.

Fluctuations in synaptic glutamate homeostasis led to aberrant activity of NMDARs, which are involved in the pathogenesis of neuropsychiatric disorders, suggesting the clinical relevance of allosteric modulators at this site. A recent preclinical study characterized an allosteric modulator, CNS4, which is responsible for enhancing the currents of NMDARs dependent on the concentration of endogenous glutamate [227]. CNS4 showed to alter the potentiation of glutamate in rat cortical, striatal, and cerebellar neurons by enhancing ions influx through native NMDAR activity, representing an unconventional candidate for TRS [227].

A further non-canonical treatment approach for schizophrenia is represented by riluzole, a drug used for amyotrophic lateral sclerosis that reduces the synaptic release of glutamate by inhibiting VGSCs and calcium currents [145,146]. Preclinical studies have also shown other mechanisms of riluzole that potentially impact glutamatergic dysfunction in schizophrenia: (i) increase in the astrocytic reuptake of glutamate [228]; (ii) neuroprotective effect via the upregulation of glutamate transporter 1 (GLT-1) in a voltage-sensitive ion channels blockade independent manner [229]; (iii) improved cortical glutamate cycling [230]; and (iv) reducing the size of the releasable presynaptic glutamate, inhibiting protein kinase C (PKC)-dependent Munc18-1 phosphorylation [231] (Figure 3). A recent functional magnetic resonance imaging (fMRI) study measuring resting anterior cingulate cortex (ACC)-functional connectivity examined the ability of riluzole to modulate glutamate metabolite levels and functional cortical connectivity, proposing its role in normalizing Glx levels and increasing cortical connectivity in TRS patients [232]. These results also indicated that glutamatergic function and cortical connectivity were linked to the cognitive symptoms in TRS and thus pharmacologically modulated [232]. An RCT study with 50 chronic schizophrenia patients showed that riluzole significantly reduced the severity of negative symptoms compared to the HC group [233]. In this context, riluzole might have a beneficial effect on glutamatergic excitotoxicity based on the spatiotemporal boundaries of NMDAR hypofunction [234]. It has been hypothesized that NMDAR hypofunction occurs initially during the maturation of GABAergic neurons, causing reduction in intrinsic excitability and GABA release and disinhibition of pyramidal neurons. Cortical disinhibition, in turn, could lead to increased glutamate spillover and subsequent homeostatic dysregulation of NMDAR function in pyramidal neurons. These two temporally distinct and complementary hypotheses of NMDAR hypofunction could together explain the complexity of the trajectory of schizophrenia’s pathophysiology [234].

Corbett and co-workers proposed that the antipsychotics clozapine and olanzapine may antagonize dopamine-induced and non-competitive NMDAR-associated behaviors [235]. Preclinical in vivo studies have shown that antipsychotics antagonize the mouse climb test and the locomotion and fall test in a dose-dependent fashion [235]. Non-competitive NMDAR antagonists, such as PCP and MK-801, can induce social withdrawal, locomotion and falling behavior in rodents that are selectively reversed by clozapine and olanzapine, albeit not as a direct result of blocking D1Rs or D2Rs. These results suggested that the mechanisms underlying non-competitive NMDAR antagonism may be explored to select novel non-canonical drugs for schizophrenia-negative symptoms and TRS [235].

### 3.3. Dopamine Antagonism and Trans-Synaptic Effects at the Postsynaptic Density

The postsynaptic density (PSD) is a macromolecular complex located at the postsynaptic terminals of glutamatergic synapses, detectable with electron microscopy as a disc with a surface area of approximately 0.07 μm^2^ and a thickness of 30–40 nm [236,237,238,239]. The PSD is composed of over 2100 proteins [240] organized in multiple orders of stratified molecules, including the following: receptors, i.e., NMDAR, AMPAR, mGluR type I, Kainate receptor; adaptors/scaffolds, i.e., Postsynaptic density protein 95 (PSD-95), Disrupted in schizophrenia 1 (DISC1), Stargazing, Homer, SH3 and multiple ankyrin repeat domains (Shank); cytoskeleton proteins (i.e., Tubulin, Actin, α-internexin); and enzymes [241,242]. NMDAR is the crucial structure of the PSD, representing the core of the PSD molecular machinery [243]. Pathological conditions not only modulate subunit composition, as demonstrated in a preclinical study in a PCP mice model that showed an increase in NR2A and NR2B NMDAR subunits [244] but also the complete composition of the PSD, resulting in morphological and functional modifications (e.g., spine loss in the auditory cortex in schizophrenia patients) [245,246]. Alterations in glutamate neurotransmission are also attributable to presynaptic terminals modifications in both vesicular glutamate transporters (VGLUT), whose transcript is increased through two common SNPs detected in schizophrenia patients [247], and several proteins related to glutamate release, which are reduced in the ACC of schizophrenia patients [248]. These findings are in opposition when compared with observations in TRS patients that exhibit an increase in glutamate and glutamate metabolites levels in dorsalACC evaluated by 3T-^1^H-MRS or fMRI [232,249].

This complex molecular arrangement may act as a spatio-temporal organizer with multiple converging signaling possibly exiting in the structural changes of synaptic plasticity processes [250,251]. Considering the crucial role of these events in the pathophysiology of psychiatric disorders, including schizophrenia, it is not surprising that alterations in the expression or structure of these constitutive proteins result in aberrant synaptic processes as reported by multiple evidence of several studies such as genome-wide association study (GWAS), post-mortem studies, proteomic analysis, and preclinical modeling that emphasize the relevant role of PSD structure in the intricate scenario of the genetic architecture and pathophysiology of schizophrenia and TRS [251,252,253,254,255,256,257,258,259,260,261,262,263,264,265]. Growing evidence points not only to functional alterations associated with genetic and structural changes in PSD proteins but also to the possibility of aberrant spatio-temporal and region- and cell-dependent modulations of specific domains in their structure, resulting in dysfunction that could underlie the psychopathology of the disease [266,267,268,269].

### 3.4. Antipsychotics and PSD Modulation: A Relevant Non-Canonical Antipsychotic-Induced Effect?

Consistent with the position of PSD in the mechanisms involved in synaptic plasticity, several studies have demonstrated the centrality of this structure in the effect of antipsychotic drugs and the role of dopamine occupancy [270,271,272,273,274]. Here, we report the effects of different antipsychotics on the most abundant PSD proteins.

#### 3.4.1. Homer Proteins and Splicing Variants

Homer is a family of proteins located at the PSD associated with schizophrenia, as demonstrated by genetic clinical and preclinical studies investigating the *Homer1* gene [254,275,276]. This gene originates from two different types of molecules: the constitutive isoforms (Homer1b/c) and the inducible transcripts (Homer1a—Ania3) [277,278]. *Homer1a* is an immediate early gene (IEG), and it can be induced by dopaminergic and glutamatergic manipulations [279,280]. It acts as a “dominant negative” for the constitutive form, reducing the formation of the tetrameric structures formed by Homer1b, thus triggering direct changes in PSD architecture and signaling [278,281,282].

**Figure 4:**
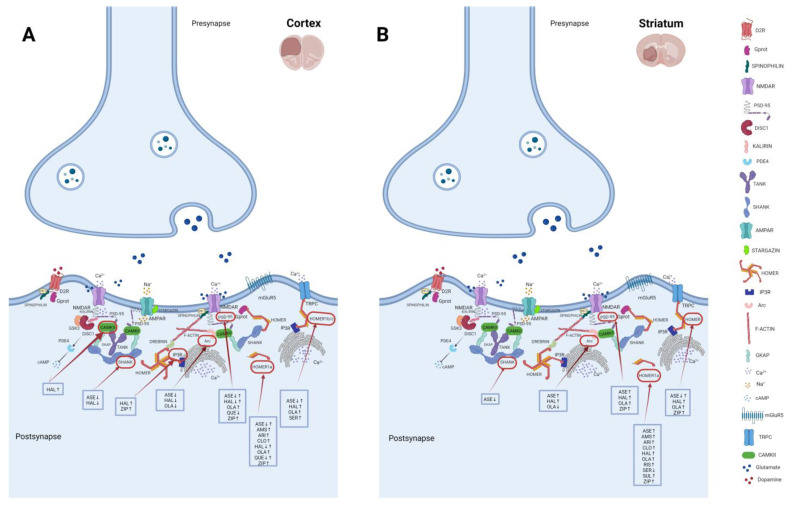
Antipsychotics and PSD modulation as relevant unconventional antipsychotic action. Panel (**A**). Modulation of PSD proteins by antipsychotics in rat cortical regions. Panel (**B**). Modulation of PSD proteins by antipsychotics in rat striatal regions. AMPAR = α-amino-3-hydroxy-5-methyl-4-isoxazolepropionic acid receptor; CAMKII = Calcium–calmodulin (CaM)-dependent protein kinase II; F-ACTIN = Filamentous actin; GKAP = guanylate kinase-associated protein; GSK3 = Glycogen Synthase Kinase 3 Beta; IP3R = IP3 (inositol 1,4,5-trisphosphate) receptor; mGluR5 = metabotropic glutamate receptor 5; NMDAR = N-Methyl-D-aspartic acid receptor; SHANK = SH3 and multiple ankyrin repeat domains; TANK = TRAF Family Member Associated NFKB Activator; PP-1 = protein phosphatase 1; D2R = dopamine receptor D2; Gprot = G protein; cAMP = cyclic adenosine monophosphate; AKT = protein kinase B; PKC = protein-chinasi C; GSK3 = glycogen synthase kinase-3; Ca^2+^ = calcium ion; DISC1 = disrupted-in-schizophrenia 1; PDE4 = phosphodiesterase 4A; Na^+^ = sodium ion; TRPC = transient receptor potential cation channel; PSD-95 = Postsynaptic density protein-95 kDa; HAL = haloperidol; CLO = clozapine; RIS = risperidone; OLA = olanzapine; SER = sertindole; ZIP = ziprasidone; APZ = aripiprazole; AMS = amisulpride; SUL = sulpride; ASE = asenapine. Created with BioRender.com on 7 March 2023.

Several preclinical studies have investigated the effects of antipsychotics and other drugs in the modulation of Homer1a and Homer1b/c by evaluating the involvement of different receptor profiles, particularly based on D2R occupancy and the timing of administration in the variable induction of these molecules, and thus in the different modes of synapse restructuring (Figure 4 and Table 1). Considering the effect of haloperidol and ziprasidone at D2R different from clozapine, it was found that acute haloperidol administrations, at low (0.25 mg/kg), medium (0.5 mg/kg), and high (0.8 mg/kg) doses are responsible for an increase in Homer1a transcript levels, as well as for Ania-3 splice variant for haloperidol 0.8 mg/kg [283], in the striatum as well as ziprasidone at low (4 mg/kg) and high (10 mg/kg) doses [283,284,285,286,287,288,289,290,291,292,293,294,295,296,297,298,299] (Table 1). Chronic administration and sacrifice after 90′ from the last injection of haloperidol at 0.8 mg/kg and 0.25 mg/kg, but not at 0.5 mg/kg, as well as ziprasidone at 10 mg/kg, are responsible for an increase in Homer1a levels in the striatum [283,284,286,287,300]. These findings suggest that Homer1a retains its qualitative and quantitative properties even after prolonged treatments, leading to the hypothesis that it does not encounter tolerance or desensitization phenomena as observed for other IEGs, such as *c-Fos* [301,302]. On the other hand, clozapine at 15 mg/kg and ziprasidone at 10 mg/kg are responsible for increased Homer1a levels in the cortex compared to haloperidol, which causes a decrease in the same brain region after both acute and chronic administration with a reduction of neuronal activity [286,289,292,296,300] (Table 1), probably not mediated by D2R transmission but due to their remarkable serotoninergic action [284]. Chronic asenapine administration is responsible for reducing Homer1a levels in the insular cortex [300], whereas acute administration at low (0.05 mg/kg) and medium (0.1 mg/kg) doses are responsible for an increase in Homer1a levels in both cortical and subcortical regions [288]. This effect is probably related to the progressive stimulation of dopamine, noradrenaline, and serotonin efflux in PFC and NAc, strictly dependent on both 5HT_2A_ and α_2_ blockade caused by asenapine [303] (Table 1). Asenapine and haloperidol chronic administration significantly shifted the Homer1a/Homer1b/c ratio toward Homer1b expression in cortical regions and toward Homer1a in subcortical regions with different values depending on the doses of antipsychotics. These findings suggest that the variable spatial expression pattern of both Homer’s constitutive and inducible forms may exert a differential impact on postsynaptic plasticity based on their opposite actions [288,300,304]. An increase in the striatum Homer1a levels is also detected after risperidone (3 mg/kg) and olanzapine (2.5 mg/kg) acute administration compared to sulpride, although the increase in the same levels after the administration of haloperidol at 0.8 mg/kg is greater probably related to the different affinity for D2R [305] (Table 1). Intriguingly, at the topographical evaluation, the signal of Homer1a after haloperidol, risperidone and olanzapine follows a dorsolateral–ventromedial gradient in the striatum [290].

The same distribution is followed by medium-sized spiny neurons, which are the striatal cells prominently expressing Homer1a induced by antipsychotics [306,307] that could be related to the graded organization of the caudate–putamen [290] and with a different dorsal-to-ventral distribution of D1R and D2R [308,309,310,311].

In conclusion, antipsychotics may progressively recruit IEGs expression in cortical and subcortical regions based on their different timing and doses of administration, reflecting that a fine-tuned dose-dependent modulation of PSD proteins also depends on receptor occupancy at different doses underlying clinical efficacy, response, and side effects relevant to schizophrenia treatment response and resistance.

**Table 1:** Modulation of PSD proteins’ transcripts in different rat brain regions by antipsychotics and other psychotropic molecules related to their receptor profiles based on Sykes and co-authors’ studies [312,313]. ↑ = increased levels; ↓ = decreased levels; M1 = primary motor cortex; M2 = supplementary motor cortex; IC = insular cortex; DMCP = dorsomedial caudate-putame; DLCP = dorsolateral caudate-putament; VMCP = ventromedial caudate-putamen; VLCP = ventrolateral caudate-putamen; ACC = anterior cingulate cortex; MC = motor cortex; SS = somatosensory cortex; AC = Auditory Cortex; FC = frontal cortex; DG = dentate gyrus; SNc = substantia nigra pars compacta, VTA = ventral tegmental area; MAC = Medial Agranular Cortex; Cab = Core of the nucleus accumbens; Sab = shell of the nucleus accumbens 5HT = serotonin; PSD = postsynaptic density; IP3R = Inositol 1,4,5-trisphosphate receptor; Arc = activity-regulated cytoskeleton-associated protein; mGluR5 = metabotropic glutamate receptor 5; αCaMKII = alpha-Ca^2+^/calmodulin-dependent protein kinase-II; Shank = multiple ankyrin repeat domains; DAT = Dopamine Transporter; ERK = Extracellular signal-regulated kinases.

Gene	Drug	D2/5HT_2A_ Affinity Ratio	Dose	Duration	Effects on Gene Expression/Rat Brain Region	Brain Region of Interest	Reference
Homer 1a	Haloperidol	0.00087	0.8 mg/kg	Acute treatment	↑ in striatum	DMCP, DLCP, VMCP, VLCP, CAb, SAb	[283,284,286,287,288,289,291,292,293,294,295,296,297,298,299]
					↓ in cortex	ACC, MAC, MC, SS, IC	[286,289,292,296]
				Chronic treatment (sacrifice after 90′ from last injection)	↑ in striatum and cortex	DMCP, DLCP, VMCP, VLCP, CAb SAb, ACC, MAC, MC, SS, IC	[283,284,286,287,300]
					↓ in cortex	MAC, MC, IC	[300]
				Chronic treatment (sacrifice after 24 h from last injection)	↑ in cortex	ACC, MAC, MC, SS, IC	[284]
			0.25 mg/kg	Acute treatment	↑ in striatum	DMCP, DLCP, VLCP	[288]
				Chronic treatment (sacrifice after 90′ from last injection)	↑ in striatum	DLCP, VLCP, DMCP, SAb	[300]
			0.5 mg/kg	Acute treatment	↑ in striatum	DMCP, DLCP, VMCP, VLCP	[288]
				Chronic treatment (sacrifice after 90′ from last injection)	↓ in cortex	MC	[300]
	Haloperidol + Valproate		0.8 mg/kg + 500 mg/kg	Acute treatment	↑ in striatum	DMCP, DLCP, VMCP, VLCP, CAb	[289]
	Ziprasidone	2.57	4 mg/kg	Acute treatment	↑ in striatum	DMCP, DLCP, VMCP, VLCP	[284]
			10 mg/kg	Acute treatment	↑ in cortex and striatum	DMCP, DLCP, VMCP, VLCP, CAb, SAb, ACC, MC, SS, IC	
				Chronic treatment (sacrifice after 90′ from last injection)	↑ in striatum	DLCP, VLCP, CAb SAb	
	Asenapine	3.39	0.05 mg/kg	Acute treatment	↑ in striatum and cortex	AC, M2, M1, SS, DMCP, DLCP, VMCP, VLCP, CAb	[288]
				Chronic treatment (sacrifice after 90′ from last injection)	↑ in striatum	DLCP	[300]
			0.1 mg/kg	Acute treatment	↑ in striatum and cortex	AC, M2, M1, SS, IC, DMCP, DLCP, VMCP, VLCP, CAb, SAb	[288]
				Chronic treatment (sacrifice after 90′ from last injection)	↓ in cortex	IC	[300]
			0.3 mg/kg	Acute treatment	↑ in striatum	DMCP, DLCP, VMCP, VLCP, CAb, SAb	[288]
	Olanzapine	1.00	2.5 mg/kg	Acute treatment	↑ in striatum and cortex	AC, M2, M1, I, DMCP, DLCP, VMCP, VLCP, CAb, SAb	[288,290]
				Chronic treatment (sacrifice after 90′ from last injection)	↑ in striatum	DLCP, SAb	[286,300]
	Sertindole	5.37	2 mg/kg	Acute treatment	↓ in striatum	SS, IC	
	GBR 12909	-	15 mg/kg	Acute treatment	↑ in cortex	SS	[283]
					↑ in striatum and cortex	FC outer, FC inner, Cingulate cortex, DLCP, VMCP, VLCP, CAb, Sab	[287]
				Chronic treatment (sacrifice after 90′ from last injection)	↑ in cortex	SS	[283]
	Aripiprazole	0.0093	12 mg/kg	Acute treatment	↑ in striatum	DMCP, DLCP, VMCP, VLCP	[287]
				Chronic treatment (sacrifice after 90′ from last injection)	↑ in striatum and cortex	FC inner, Cingulate cortex, DLCP,	
			30 mg/kg	Acute treatment	↑ in cortex	FC outer, FC inner, Cingulate cortex, SAb	[287]
	Clozapine	2.63	15 mg/kg	Acute treatment	↑ in cortex	ACC, MAC, SS, IC	[284]
					↑ in striatum	SAb	[287]
				Chronic treatment (sacrifice after 90′ from last injection)	↑ in cortex	FC inner, PC inner	
	Risperidone	1.62	3 mg/kg	Acute treatment	↑ in striatum	DLCP, VLCP	[290]
	Sulpiride	-	50 mg/kg	Acute treatment	↑ in striatum	VLCP, CAb	[290]
	Ketamine	-	25 mg/kg	Acute treatment	↑ in cortex	IC	[314]
	GBR-12909	-	30 mg/kg	Acute treatment	↑ in striatum	VMCP, SAb	[292]
	Caffeine	-	40 mg/kg	Acute treatment	↑ in striatum	DMCP, SAb	[292]
	Caffeine +. Haloperidol	-	40 mg/kg + 0.8 mg/kg	Acute treatment	↓ in striatum	DMCP, VMCP	[292]
	Nicotine + Haloperidol	-	1.5 mg/kg+ 0.8 mg/kg	Acute treatment	↑ in striatum	DMCP, CAb, SAb	[292]
	Escitalopram	-	12 mg/kg	Acute treatment	↑ in cortex	outer parietal cortex	[293]
	Citalopram	-	14 mg/kg	Acute treatment	↓ in cortex	M1, SS, IC	[289]
	Haloperidol + Escitalopram	-	0.8 mg/kg + 12 mg/kg	Acute treatment	↑ in striatum and cortex	outer parietal cortex, DLCP, VLCP, CAb	[293]
	Haloperidol + Citalopram	-	0.8 mg/kg + 14 mg/kg	Acute treatment	↑ in striatum and cortex	outer parietal cortex, DLCP, VLCP, DMCP, VMCP, CAb	[293]
	Amisulpride	0.00078	35 mg/kg	Chronic treatment (sacrifice after 90′ from last injection)	↑ in striatum and cortex	VMCP, DMCP, ACC, MAC, MC, SS, IC	[295]
				Acute treatment	↑ in striatum	VMCP, DMCP	[298]
	Ketamine	-	50 mg/kg	Acute treatment	↑ in striatum	VMCP, VLCP	[315]
	Ketamine	-	12 mg/kg	Acute treatment	↑ in striatum	CAb, SAb	[315]
	SCH-23390	-	0.5 mg/kg	Acute treatment	↑ in striatum and cortex	VLCP, CAb, SAb, MAC, MC	[296]
	L-741,626	-	2 mg/kg	Acute treatment	↑ in striatum and cortex	VMCP, DMCP, VLCP, DLCP, CAb, SAb, MAC, MC	[296]
	U-99194	-	5 mg/kg	Acute treatment	↑ in cortex	MAC, MC	[296]
	Terguride	-	0.5 mg/kg	Acute treatment	↑ in striatum	VLCP, DLCP	[296]
					↓ in cortex	MC	
	Quetiapine	0.35	30 mg/kg	Acute treatment	↑ in cortex	ACC, M2, M1	[289]
				Chronic treatment (sacrifice after 90′ from last injection)	↓ in cortex	M2, M1, SS	
			15 mg/kg	Chronic treatment (sacrifice after 90′ from last injection)	↓ in cortex	M2, M1, SS	
	Haloperidol + Mynocycline	-	0.8 mg/kg + 45 mg/kg	Acute treatment	↑ in striatum	VMCP, DMCP, DLCP	[299]
Homer 1b/c	Ziprasidone	2.57	10 mg/kg	Chronic treatment (sacrifice after 24 h from last injection)	↑ in striatum	DLCP	[284]
	Haloperidol	0.00087	0.25 mg/kg	Acute treatment	↑ in striatum	CAb	[288]
			0.5 mg/kg		↑ in striatum and cortex	M1, CAb	
			0.8 mg/kg		↑ in striatum and cortex	AC, M2, M1, CAb	
			0.8 mg/kg	Chronic treatment (sacrifice after 90′ from last injection)	↑ in striatum and cortex	DLCP, VMCP, VLCP, CAb, Sab, ACC, MAC, SS, IC	[286]
					↓ in striatum and cortex	VMCP, DMCP, MAC	[300]
	Asenapine	3.39	0.1 mg/kg	Acute treatment	↑ in striatum and cortex	AC, M2, M1, CAb	[288]
			0.3 mg/kg				
	Olanzapine	1.00	2.5 mg/kg	Acute treatment	↑ in striatum and cortex	AC, M2, M1, CAb	
	Sertindole	5.37	2 mg/kg	Chronic treatment (sacrifice after 90′ from last injection)	↑ in cortex	ACC, MAC, SS, IC	[286]
	Ketamine	-	25 mg/kg	Acute treatment	↓ in cortex	MC	
	Ketamine	-	50 mg/kg	Acute treatment	↓ in striatum and cortex	MAC, MC, DLCP	[314]
	GBR-12909	-	30 mg/kg	Acute treatment	↑ in striatum and cortex	ACC, MAC, MC, SS, IC, VMCP, DMCP, DLCP	[292]
	Nicotine	-	1.5 mg/kg	Acute treatment	↑ in striatum and cortex	MAC, SS, DMCP, VMCP, CAb, SAb	[292]
	Caffeine +. Haloperidol	-	40 mg/kg + 0.8 mg/kg	Acute treatment	↑ in striatum	MAC, SS	[292]
	Nicotine + Haloperidol	-	1.5 mg/kg + 0.8 mg/kg	Acute treatment	↑ in striatum	ACC, MAC, MC, SS, IC, VMCP, DMCP, DLCP, CAb, SAb	[292]
	SCH-23390	-	0.5 mg/kg	Acute treatment	↑ in striatum	CAb	[296]
					↓ in cortex	MC	
	L-741,626	-	2 mg/kg	Acute treatment	↓ in striatum and cortex	VLCP, SAb, MC, SS, IC	[296]
	L-745,870	-	3 mg/kg	Acute treatment	↓ in striatum and cortex	VMCP, VLCP, SAb, MC, SS	[296]
	Terguride	-	0.5 mg/kg	Acute treatment	↓ in striatum and cortex	VMCP, VLCP, CAb, SAb, MC, SS, IC	[296]
PSD-95	Ziprasidone	2.57	10 mg/kg	Chronic treatment (sacrifice after 90′ from last injection)	↑ in striatum	DMCP, DLCP, VMCP, VLCP, CAb, SAb	[284]
				Chronic treatment (sacrifice after 24 h from last injection)	↑ in striatum and cortex	VMCP, ACC, MAC, MC	
	Haloperidol	0.00087	0.25 mg/kg	Chronic treatment (sacrifice after 90′ from last injection)	↓ in cortex	ACC, MAC, MC, SS, IC	[300]
			0.5 mg/kg	Acute treatment	↑ in cortex	AC, M1	[288]
			0.8 mg/kg		↑ in striatum and cortex	ACC, M2, M1, I, DMCP, DLCP, VMCP, CAb, SAb	
				Chronic treatment (sacrifice after 90′ from last injection)	↑ in striatum and cortex	DMCP, DLCP, VMCP, VLCP, CAb, SAb, ACC, MC, SS	[284,286]
					↓ in cortex	ACC, MAC, MC, SS	[300]
				Chronic treatment (sacrifice after 24 h from last injection)	↑ in cortex	ACC, MAC, MC	[284]
	Asenapine	3.39	0.05 mg/kg	Acute treatment	↑ in striatum and cortex	AC, M2, M1, SS, I, DMCP, DLCP, VMCP, VLCP, CAb, SAb	[288]
			0.1 mg/kg	Acute treatment	↑ in striatum and cortex	AC, M2, M1, SS, I, DMCP, DLCP, VMCP, VLCP, CAb, SAb	[288]
				Chronic treatment (sacrifice after 90′ from last injection)	↓ in cortex	ACC, MAC, MC, SS, IC	[300]
			0.3 mg/kg	Acute treatment	↑ in striatum and cortex	ACC, M2, M1, SS, I, DMCP, DLCP, VMCP, VLCP, CAb, SAb	[288]
	Olanzapine	1.00	2.5 mg/kg	Acute treatment	↑ in striatum and cortex	ACC, M2, M1, SS, I, DMCP, DLCP, VMCP, VLCP, CAb, SAb	[288]
	Ketamine	-	25 mg/kg	Acute treatment	↓ in striatum	VMCP	[314]
	Ketamine	-	50 mg/kg	Acute treatment	↓ in striatum	VMCP	[314]
	MK-801	-	0.8 mg/kg	Acute treatment	↓ in striatum	VMCP, VLCP	[314]
	Haloperidol + Valproate	-	0.8 mg/kg + 500 mg/kg	Acute treatment	↑ in striatum and cortex	ACC, SS, DMCP	[289]
	Quetiapine	0.35	15 mg/kg	Acute treatment	↓ in cortex	M2, M1	[289]
	Citalopram	-	14 mg/kg	Acute treatment	↑ in striatum and cortex	ACC, M2, SS, IC, DMCP, DLCP, VMCP, VLCP, CAb, SAb	[289]
IP3R	Haloperidol	0.00087	0.8 mg/kg	Chronic treatment (sacrifice after 24 h from last injection)	↑ in cortex	MAC	[284]
	Ziprasidone	2.57	10 mg/kg				
Arc	Haloperidol	0.00087	0.25 mg/kg	Acute treatment	↓ in cortex	M2, M1, SS, I	[288]
				Chronic treatment (sacrifice after 90′ from last injection)	↓ in cortex	ACC, MAC, MC, SS, IC	[300]
			0.5 mg/kg	Acute treatment	↑ in striatum	DLCP, VLCP, CAb, SAb	[286]
				Chronic treatment (sacrifice after 90′ from last injection)	↓ in cortex	ACC; MAC, MC, SS	[300]
			0.8 mg/kg	Acute treatment	↑ in striatum	DMCP, VMCP, DLCP, VLCP, CAb, SAb	[286,298,299]
					↓ in cortex	AC, M2, SS	[286]
				Chronic treatment (sacrifice after 90′ from last injection)	↓ in cortex	ACC; MAC, MC, SS	[300]
	Asenapine	3.39	0.1 mg/kg	Acute treatment	↓ in cortex	AC, M2, M1	[288]
				Chronic treatment (sacrifice after 90′ from last injection)	↓ in cortex	MC, SS, IC	[300]
			0.3 mg/kg	Acute treatment	↑ in striatum	DLCP, VLCP, CAb	[288]
				Chronic treatment (sacrifice after 90′ from last injection)	↓ in cortex and stritum	MAC, MC, SS, IC, VMCP	[300]
			0.05 mg/kg	Chronic treatment (sacrifice after 90′ from last injection)	↑ in striatum	DLCP	[300]
	Ketamine	-	25 mg/kg	Acute treatment	↑ in cortex	MAC, MC, SS, IC	[314]
	Ketamine	-	50 mg/kg	Acute treatment	↑ in cortex	ACC, MAC, MC, SS, IC, CAb, SAb	[314]
	Memantine	-	5 mg/kg	Acute treatment	↑ in cortex	MAC	[314]
	MK-801	-	0.8 mg/kg	Acute treatment	↑ in cortex	ACC, SS, CAb, SAb	[314]
	Caffeine	-	40 mg/kg	Acute treatment	↓ in cortex and striatum	IC, DMCP, VMCP, VLCP, CAb	[292]
	Nicotine	-	1.5 mg/kg	Acute treatment	↓ in cortex and striatum	MC, IC, VMCP	[292]
	GBR-12909	-	30 mg/kg	Acute treatment	↑ in striatum	CAb	[292]
	Caffeine	-	40 mg/kg	Acute treatment	↓ in striatum	IC, DMCP, VMCP, VLCP, CAb	[292]
	Caffeine +. Haloperidol	-	40 mg/kg + 0.8 mg/kg	Acute treatment	↓ in cortex and striatum	MC, SS, IC, VMCP, CAb	[292]
	Nicotine + Haloperidol	-	1.5 mg/kg + 0.8 mg/kg	Acute treatment	↑ in striatum	DMCP, CAb	[292]
	Olanzapine	1.00	2.5 mg/kg	Chronic treatment (sacrifice after 90′ from last injection)	↓ in cortex and striatum	ACC, MAC, MC, SS, IC, DMCP, VMCP	[300]
	Mynocycline	-	45 mg/kg	Acute treatment	↑ in striatum	DMCP, DLCP, VMCP, VLCP, SAb	[300]
	Haloperidol + Mynocycline	-	0.8 mg/kg + 45 mg/kg	Acute treatment	↑ in striatum	ACC, MAC, MC, SS, IC, DMCP, DLCP, VLCP	[300]
c-Fos	Haloperidol	0.00087	0.25 mg/kg	Acute treatment	↑ in striatum	VMCP, DLCP, SAb	[288]
			0.5 mg/kg			VMCP, DLCP, VLCP, SAb	
			0.8 mg/kg			DMCP, DLCP, VMCP, VLCP, CAb, SAb	[283,288,297]
	Haloperidol + D-cycloserine	-	0.8 mg/kg + 20 mg/kg	Acute treatment	↑ in striatum	DLCP, CAb, SAb	[297]
	Clozapine+ D-cycloserine	-	15 mg/kg + 20 mg/kg	Acute treatment	↑ in striatum	DLCP, CAb, SAb	[297]
	Clozapine	2.63	15 mg/kg	Acute treatment	↑ Nucleus accumbens	CAb, SAb	[297]
	Asenapine	3.39	0.3 mg/kg	Acute treatment	↑ in striatum	DMCP, DLCP, VMCP, VLCP, CAb, SAb	[288]
	Olanzapine	1.00	2.5 mg/kg	Acute treatment	↑ in striatum	VMCP, VLCP	[288]
	MK-801	-	0.8 mg/kg	Acute treatment	↑ in cortex	ACC, SS, IC	[314]
	Ketamine	-	25 mg/kg	Acute treatment	↑ in cortex	IC	[314]
	Ketamine	-	50 mg/kg	Acute treatment	↑ in cortex	MAC, IC	[314]
	Memantine	-	5 mg/kg	Acute treatment	↑ in cortex	MAC, SS	[314]
	Amisulpride	0.00078	10 mg/kg	Acute treatment	↑ in striatum	DMCP, VMCP	[298]
			35 mg/kg	Acute treatment	↑ in striatum	DMCP, VMCP	[298]
Zif-268	Haloperidol	0.00087	0.25 mg/kg	Acute treatment	↑ in striatum	DMCP, DLCP, VMCP, VLCP, SAb	[288]
			0.5 mg/kg		↑ in striatum	DMCP, DLCP, VMCP, VLCP, CAb, SAb	[288]
			0.8 mg/kg		↑ in striatum and cortex	DMCP, DLCP, VMCP, VLCP, CAb, SAb, M1, SS, IC	[288,298]
	Asenapine	3.39	0.05 mg/kg	Acute treatment	↑ in striatum	DMCP, DLCP, VMCP, VLCP, SAb	[288]
			0.1 mg/kg				
			0.3 mg/kg				
	Olanzapine	1.00	2.5 mg/kg	Acute treatment	↑ in striatum	DLCP, VLCP, SAb	[288]
	Amisulpride	0.00078	10 mg/kg	Acute treatment	↑ in striatum	DMCP, VMCP	[272]
			35 mg/kg	Acute treatment	↑ in striatum and cortex	DMCP, VMCP, ACC, M1	[298]
Ania-3	Haloperidol	0.00087	0.8 mg/kg	Acute treatment	↑ in striatum and cortex	DMCP, DLCP, VMCP, VLCP, SAb, ACC, MAC	[288]
					↑ in striatum	DMCP, DLCP, VMCP, VLCP, SAb, CAb	[257,260,261,267]
				Chronic treatment (sacrifice after 90′ from last injection)	↑ in striatum	DMCP, DLCP, VMCP, VLCP, CAb	[283,287]
	GBR 12909	-	15 mg/kg	Acute treatment	↑ in cortex and striatum	M2, M1, DMCP, VMCP, VLCP, SAb, PC outer, PC inner, DLCP	[283,287]
				Chronic treatment (sacrifice after 90′ from last injection)	↑ cortex	SS	
	Quetiapine	0.35	30 mg/kg	Chronic treatment (sacrifice after 90′ from last injection)	↑ in striatum	SAb	[283]
	Aripiprazole	0.0093	12 mg/kg	Acute treatment	↑ in striatum	DMCP, DLCP, VMCP, VLCP	[287]
				Chronic treatment (sacrifice after 90′ from last injection)	↑ in striatum	DLCP, VLCP	
	Haloperidol + Escitalopram	-	0.8 mg/kg + 12 mg/kg	Acute treatment	↑ in striatum and cortex	Inner frontal cortex, DLCP, VLCP	[293]
	Haloperidol + Citalopram	-	0.8 mg/kg + 14 mg/kg	Acute treatment	↑ in striatum	DLCP, VLCP,	[293]
	Amisulpride	0.00078	10 mg/kg	Acute treatment	↓ in cortex	M1	[298]
			35 mg/kg	Acute treatment	↑ in striatum	DMCP, VMCP	[298]
mGluR5	Haloperidol	0.00087	0.8 mg/kg	Chronic treatment (sacrifice after 90′ from last injection)	↑ in striatum and cortex	DMCP, DLCP, VMCP, ACC, MC	[286]
	Sertindole	5.37	2 mg/kg	Chronic treatment (sacrifice after 90′ from last injection)	↑ in striatum and cortex	DMCP, DLCP, VMCP, ACC, MC	[286]
	SCH-23390	-	0.5 mg/kg	Acute treatment	↑ in striatum and hippocampus	DMCP, CAb, CA3, DG	[296]
αCaMKII	Haloperidol	0.00087	0.8 mg/kg	Chronic treatment (sacrifice after 90′ from last injection)	↓ in cortex	MC	[286]
	Ketamine	-	12 mg/kg	Acute treatment	↑ in striatum and brainstem	DMCP, DLCP, VMCP, VLCP, CAb, SAb, SNc and VTA	[315]
Homer 2	Haloperidol	0.00087	0.8 mg/kg	Chronic treatment (sacrifice after 90′ from last injection)	↑ in lateral Septum	Intermediate and ventral septum	[287]
	Clozapine	2.63	15 mg/kg	Chronic treatment (sacrifice after 90′ from last injection)	↑ in lateral Septum	Intermediate septum	[287]
DAT	Ketamine Ketamine	-	12 mg/kg	Acute treatment	↑ in brainstem	SNc and VTA	[315]
			50 mg/kg				
Shank1	Haloperidol	0.00087	0.25 mg/kg	Chronic treatment (sacrifice after 90′ from last injection)	↓ in cortex	ACC, IC	
			0.5 mg/kg		↓ in cortex	ACC, IC	[300]
	Asenapine	3.39	0.05 mg/kg	Chronic treatment (sacrifice after 90′ from last injection)	↓ in cortex and striatum	ACC, IC, VMCP, VLCP, DMCP, DLCP, CAb, SAb	[300]
			0.1 mg/kg		↓ in cortex	ACC, IC	[300]
			0.3 mg/kg		↓ in cortex	IC	[300]
ERK	Valproate	-	500 mg/kg	Acute treatment	↑ in striatum and cortex	ACC, M2, M1, SS, VMCP, VLCP, DMCP, DLCP, SAb	[289]
	Haloperidol + Valproate	-	0.8 mg/kg + 500 mg/kg	Acute treatment	↑ in striatum and cortex	ACC, M2, M1, SS, IC, VMCP, VLCP, DMCP, DLCP	[289]
	Quetiapine + Valproate	-	30 mg/kg + 500 mg/kg	Acute treatment	↑ in striatum and cortex	ACC, M2, M1, SS, DMCP, DLCP, VMCP, SAb	[289]
Norbin	Haloperidol	0.00087	0.8 mg/kg	Acute treatment	↑ in striatum and cortex	VLCP, DLCP, IC	[298]

#### 3.4.2. PSD-95

PSD-95 is a membrane-associated guanylate kinase (MAGUK) scaffolding protein interacting with NMDAR through its NR2 subunit, AMPAR through stargazing, shaker-type potassium channels, cell adhesion molecules neuroligins (NLGNs), transmembrane protein Disintegrin, and metalloprotease 22 (ADAM22) [316,317,318]. PSD95 interacts also with Homer b/c long isoforms to build clusters that connect mGluRs with NMDAR [319]. PSD-95 appears to be central in the organization of the trans-synaptic complex formed by NLGN1 and neurexins, postsynaptically and presynaptically, respectively. On the other hand, it is involved in the shaping of leucine-rich glioma-inactivated protein 1 (LGI1) and ADAM22 [316,320,321] complex, controlling the fluctuation and amplitude of postsynaptic currents and, more extensively, being involved in the plasticity and strength of excitatory signaling [322,323,324]. Based on these observations, it is not surprising that genetic variants in the gene encoding for this protein can result in brain disorders such as intellectual disability (ID), epilepsy, hypotonia, autism spectrum disorder (ASD), attention deficit hyperactivity disorder (ADHD), movement disorders, and schizophrenia [325,326,327,328,329,330,331,332,333,334,335].

PSD-95 seems to have the same distribution of other constitutive genes, such as Homer1b/c, when modulated by asenapine or olanzapine, which share a receptor profile that also involves other monoamines stimulating serotonergic and adrenergic receptors. An increase was found in the signal intensity of PSD-95 throughout all subregions of the cortex and striatum, as well as in the NAc, by asenapine at different doses (0.05 mg/kg, 0.1 mg/kg, 0.3 mg/kg) as well as by olanzapine at 2.5 mg/kg [288] probably due to the progressive stimulation of dopamine, noradrenaline, and serotonin efflux in PFC and NAc related to both 5HT_2A_ and α_2_ adrenoreceptors blockade [303]. Conversely, PSD-95 was induced in ACC and primary motor cortex (M1) by haloperidol at 0.5 mg/kg, and in ACC, supplementary motor cortex (M2), M1, insular cortex (IC), dorsomedial caudate-putamen (DMCP), dorsolateral caudate-putament (DLCP), ventromedial caudate-putamen (VMCP), core (AcCo), and shell (AcSh) of NAc by haloperidol at 0.8 mg/kg [288]. A preclinical study suggests that PSD-95 expression is significantly increased in the striatum of rats sacrificed after 90′ from the last injection in chronic administration of haloperidol and ziprasidone [284]. In rats sacrificed after 24 h, the chronic administration of ziprasidone is able to increase PSD-95 levels in the striatum [284]. Finally, both antipsychotics induce the expression of PSD-95 in the cortex at 90′ and 24 h from the last injection [284]. Chronic antipsychotic administration may significantly modulate PSD-95 expression differently from acute antipsychotics [294] according to the view that prolonged antipsychotic treatments may trigger neuroplastic changes [336], favoring the recruitment at the PSD of the molecules involved in synaptic signaling and organization. Considering the physical and functional interaction between PSD-95 and NR2A, relevant for NMDAR stabilization, NR2A-containing NMDAR leads to an attenuation of glutamate receptor signaling and a loss of the spine [255]. Given the role of PSD-95 in inhibiting D1R-mediated signaling [337], the increase in its levels may represent an indirect mechanism that contributes to reverting hyperdopaminergic conditions in schizophrenia [284]. In rats PFC, D1R, and NMDAR co-localize in single pyramidal neurons and interneurons [338]. NMDAR is not affected by the cAMP-dependent activity of D1R in primary rat prefrontal cultures. In contrast, D1R activation enhanced NMDAR-mediated Ca^2+^ release, an effect blocked by a PKA inhibitor. This finding may suggest that D1R promotes NMDAR-Ca^2+^ signaling via a PKA-dependent mechanism [338]. On the other hand, D2R stimulation is responsible for inhibiting glutamate release, reducing the excitability of medium-spiny neurons [339,340,341]. These opposite effects of D1R/NMDAR and D2R/NMDAR interplay in the striatum may be relevant to both the pathophysiology and treatment of schizophrenia [342]. The stimulation of D2R, as reported in schizophrenia, could worsen an already deficient NMDAR transmission in the cortical regions [342]. On the other hand, D2R blockade induced by antipsychotic drugs could restore striatal glutamatergic transmission, cortico-striatal connectivity, and synaptic plasticity, which are relevant to cognitive processes [342].

#### 3.4.3. Shank Proteins

Shank is a PSD protein whose mutations were found to be strongly associated with ASD, ID, and schizophrenia [343], and that has been demonstrated to be instrumental in the functional and physical coupling of NMDAR and mGluR5 [344]. *De novo* mutation (R1117X) in the Shank isoform ProSAP2/Shank3 identified in a patient affected by schizophrenia is responsible for an accumulation of mutated ProSAP2/Shank3 in hippocampal neurons within the nucleus, resulting in an alteration in the transcription of several genes, such as *synaptotagmin 1* and *leucine-rich repeat transmembrane neuronal protein 1* (*LRRTM1*) and a reduction in synaptic density [345]. A preclinical study in mice also demonstrated the modulation of this molecule by antipsychotics, in particular inducing a reduction in different cortical and subcortical regions after the chronic administration of haloperidol and asenapine at different doses, confirming the view that the modulation of synaptic plasticity processes by these molecules could be finely tuned by antipsychotics depending on their doses and timing of administration [295].

#### 3.4.4. Dopamine Regulation of Key Early Genes

There is robust evidence, embedded in almost 30 pieces of research, on the role of dopaminergic receptors in mediating the induction/activation of several IEG programs of immediate and long-lasting type [346,347,348,349,350]. Therefore, the possibility of exploiting early gene induction to unveil new and non-conventional targets of antipsychotic therapy is not surprising.

##### Activity-Regulated Cytoskeleton-Associated Protein

The activity-regulated cytoskeleton-associated protein (Arc) is an IEG encoding for a protein characterized by a C-terminus involved in the regulation of cytoskeleton structure through the interaction with F-actin [351], microtubules, and microtubule-associated protein 2 (MAP2) [352]. *Arc*-KO mice exhibit a decrease in the proportion of thin and filopodia-like protrusions as well as in the density of spines in hippocampal neurons [353], probably due to NMDAR hypofunction [354], suggesting a crucial role of Arc in the regulation of dendritic spine density and morphology [355]. Arc is also strongly modulated in multiple brain regions by antipsychotics treatment and by the different durations of drug administration: Arc mRNA expression was found to be strongly reduced in the cortex after chronic administration at the highest dose of asenapine (0.3 mg/kg) and olanzapine (2.5 mg/kg) [300], probably due to their antagonism at 5-HT_2A_ [355,356,357,358]. By contrast, haloperidol can strongly induce the *Arc* gene compared to both asenapine and olanzapine, leading to the hypothesis that an upregulation of the IEG in the striatum could be related to a perturbation of dopamine neurotransmission through D2R occupancy [286,300].

##### The Immediate Early Genes *c-Fos* and *Zif-268*

C-fos is a transcription factor that, when induced, forms the heterodimer activator protein-1 (AP-1), which binds to the promoter region of numerous target genes [359]. Generally, the intracellular levels of c-Fos are relatively low without any stimuli but are transiently and rapidly induced by various extracellular stimuli [360]. In this regard, it is considered a putative molecular marker of activation by antipsychotics in the brain [297]. In a previous preclinical study, the c-Fos signal was incrementally recruited in the various areas of the striatum with the increase in haloperidol doses [288], different from Zif-268, which could also explain the progressive reduction in motion related to striatum recruitment more than D2R antagonism; meanwhile, the absence of induction in cortical regions could be explained considering the scarce affinity to serotonergic receptors by this typical antipsychotic [287,288]. On the other hand, amisulpride acts as an antagonist at the D2R/D3R despite the low liability for motor side effects, which is probably due to its effects at the presynaptic terminal at a low dose (0.05 mg/kg), elicited a significant increase in c-Fos and Ania levels in the medial regions of striatum compared to vehicle, consistent with the hypothesis that this type of compound may selectively target limbic forebrain regions [298].

### 3.5. Intracellular Mechanisms and Antipsychotics Signaling: Non-Canonical D2R Druggable Targets in Schizophrenia

#### 3.5.1. Modulation of Neuroprotective Molecules as Targets in Schizophrenia

Some genes involved in neuroprotection appear to be relevant for the pathophysiology of schizophrenia and other neurodevelopmental disorders and may represent novel non-canonical strategies for the treatment of these diseases. Among them, Notch Receptor 4 (NOTCH4) regulates neuronal and glial signaling and maturation [361,362,363], while DISC1 controls the proliferation of neural progenitors [364], modulates the positioning of pyramidal neurons in the cortex [365], and regulates the sensitization of D2R [366]. A post-mortem study on the striatum of patients affected by schizophrenia demonstrated an increased interaction between D2R and DISC1 with the formation and accumulation of intraneural complexes [367]. They are involved in a potential neuroprotective effect, and also seem to exert a modulatory effect on synaptic plasticity, which is able to decrease long-term potentiation (LTP) in the hippocampus of mice [368]. Zheng and co-authors, using FRET and stochastic optical reconstruction microscopy (STORM) techniques in murine striatal neurons, demonstrated that the D2R-DISC1 complex is also able to influence intracellular signaling and affect the growth of dendritic spines. This peculiar interaction could represent a potential D2R-mediated unconventional MOA at the dopamine receptor. In contrast, uncoupling D2R-DISC1 interaction, through the trans-activator of transcription (TAT)-D2pep (TAT-D2pep), reduced the previously discussed effect, resulting in a neuroprotective and preventive effect for neurite outgrowth and dendritic spines by associating with the downregulation of synaptophysin and PSD-95 expression [369].

Brain-derived neurotrophic factor (BDNF) and tropomyosin receptor kinase B (TrkB) modulate synaptic plasticity [349,370,371] and play a role in LTP [372,373,374]. Multiple evidence has shown that neuromuscular synapses’ acute exposure to BDNF increases the frequency of spontaneous miniature excitatory postsynaptic current [375], relevant for hippocampal development [376]. BDNF also acts on hippocampal synaptic transmission, causing potentiation, probably through TrkB [377], of acute glutamate release in the presynaptic terminal [378,379] and is involved in the mobilization and docking of synaptic vesicles [380] through indirect interaction with Myosin VI (Myo6) [381,382], and TrkB via PDZ Domain Containing Family Member 1 (GIPC1), with subsequent neurotransmitter release [383]. The GIPC1 and Myo6 complex is involved in enhancing LTP at hippocampal CA3-CA1 synapses in the postnatal period and improving the synaptic plasticity processes [383], whereas Trk receptors within neurophysins are also directly involved in the modulation of dopaminergic, glutamatergic, GABAergic, and acetylcholinergic neurotransmission, representing another unconventional MOA based on the canonical neurotransmitters systems [370,378,384]. BDNF-induced glutamate release in the presynaptic terminal was also mediated by the phosphorylation of signal-regulated kinase 1/2 (ERK1/2) [379] via tyrosine phosphatase Shp-2 with subsequent neuroprotective effect [385]. In striatal neurons cultures, D1R interacts with Shp-2 determining ERK1/2 activation [386], underlining the crucial role of dopamine neurotransmission also in neuroprotective and anti-inflammatory action, and opening the possibility of identifying new uncanonical druggable molecules.

##### Antipsychotic Modulation of Neuroprotective Processes

Haloperidol and aripiprazole can decrease the formation of D2R-DISC1 complexes by blocking D2R, although haloperidol does not seem to significantly affect neuroprotection [387]. Ray and coauthors showed that BDNF expression was downregulated in layers IV and V of the DLPFC, layer VI of the ACC, layer VI of the inferior temporal gyrus, and layer V and/or VI of the superior temporal gyrus in patients with schizophrenia [388], and other studies have found decreased BDNF expression in the PFC and hippocampus of patients with schizophrenia [389] consistent with the hypothesis of its neuroprotective effect.

Beaulieu and colleagues showed that the increased dopaminergic stimulation of D2R results in decreased Akt activity and serine phosphorylation of glycogen synthase kinase 3 (GSK3) [390]. In coherence with their receptor profiles and D2R occupancy, aripiprazole, clozapine, and haloperidol differentially modulate the AKT-GSK-3β cascade and subsequent dendritic plug. Clozapine and aripiprazole can increase the phosphorylation of Akt (Thr308 and Ser473) and GSK-3β (Ser9), high doses of clozapine decrease the phosphorylation of Akt, whereas haloperidol reduces the phosphorylation of both Akt and GSK-3β [273] (Table 2). Chronic high doses of haloperidol or risperidone are responsible for the downregulation of BDNF transcripts in the hippocampus [391], and a decrease in serum BDNF concentration is reported in patients affected by schizophrenia treated with clozapine [392]. Clozapine may act through non-canonical intracellular mechanisms, for example, increasing neurogenesis in the hippocampus [393], regulating protein degeneration [394,395,396], and preventing apoptosis and cortical atrophy (with prevention of neural DNA fragmentation and pro-telomeric degeneration) [397,398,399,400]. Clozapine may also regulate the release of nerve growth factor (NGF) and BDNF supporting neural survival and differentiation [401,402,403,404,405]. Preclinical studies in rats have shown that chronic clozapine administration may enhance BDNF/TrkB signaling and increase CREB expression in the frontal cortex and hippocampus [406,407], which induces NGF participation in the processes of neuronal differentiation and growth [408] (Table 2). Signaling on CREB induced by clozapine involves two major upstream kinases, Akt and GSK-3β, going on to regulate dendritic remodeling and spine shape [409,410]. Schizophrenia patients, compared to controls, have lower levels of Akt and decreased phosphorylation of GSK-3β in the brain and peripheral lymphocytes [411]. Clozapine activates Akt [412,413,414,415] and increases the phosphorylation of GSK-3β in the PFC, striatum, and ventral midbrain [273,416,417] and appears to have an antiproliferative function through the inhibition of ErbB kinases [418] (Table 2).

Regarding antiproliferative effect, lumateperone could improve NMDAR- and AMPAR-mediated D1R signaling and increase dopamine and glutamate release in the medial PFC in rats via mTORC [419,420,421], regulating dopamine neurotransmission but is also involved in maintaining the integrity of the BBB and modulating molecules such as claudin-5 and intercellular adhesion molecule 1 (ICAM1) in an antiproliferative manner [421] (Table 2) (Figure 3).

**Table 2:** Intracellular mechanism of antipsychotic drugs and dopaminergic correlation. Abbreviations: Akt = protein-kinase B; AMPA = α-amino-3-hydroxy-5-methyl-4-isoxazolepropionic acid; BDNF = brain-derived neurotrophic factor; CREB = cAMP response element-binding protein; D2R = dopamine D2 receptor; DISC1 = disrupted in schizophrenia 1; ErbB = erythroblastic leukemia viral oncogene homologue; ERK = extracellular signal-regulated kinase; GSK3 = glycogen synthase kinase 3; mTOR = mammalian target of rapamycin; NMDA = N-methyl-D-aspartate; DAT = Dopamine transporter.

Antipsychotic Treatment	Molecular Targets	Dopaminergic Modulation	Molecular Mechanism	Biological Role	References
Aripiprazole	Akt Thr308 and Ser473 and GSK-3β Ser9 phosphorylation	D2R-signaling	AKT-GSK-3β cascade	Neuroprotective action	[273,390]
Aripiprazole, haloperidol	decrease D2R-DISC1 complexes	D2R-signaling	DISC1	Neuroprotective action	[387]
Clozapine	BDNF-CREB	D2R-signaling	decreased Akt activity and phosphorylation of GSK3	Neuronal survival and regulation of synaptic plasticity	[390,412,413]
	NMDAR	DAT	Akt-GSK3	Neuroprotective action	[422,423]
	Akt Thr308 and Ser473 and GSK-3β Ser9 phosphorylation	D2R-signaling	Akt-GSK-3β cascade	Neuroprotective action	[273,390]
Clozapine, olanzapine, quetiapine	ErbB kinases, PIK3CD	D2R-signaling	ErbB4-PI3K-Akt pathway	Antiproliferative, neuroprotective action	[424] [425]
Haloperidol	Akt Thr308 and Ser473 and GSK-3β Ser9 phosphorylation.	D2R-signaling	Akt-GSK-3β cascade	Neuroprotective action	[273,390]
	ERK1/2	D2R-signaling	Akt/mTOR pathway	Neuroprotective action	[426]
**Lumateperone**	NMDA and AMPA receptors	D1R-signaling	mTOR pathway	Neuroprotective role	[426]

#### 3.5.2. Neuroinflammation Mechanisms in Schizophrenia

During inflammatory processes, quinolinic acid and kynurenic acid are mainly synthesized in microglia and in astrocytes, respectively. The first is an NMDAR agonist with potential excitotoxicity [427], while the second is an NMDAR antagonist [428] that could induce cognitive dysfunctions, consistent with the NMDAR hypofunction hypothesis of schizophrenia [429,430,431]. These molecules, together with the excess in glutamate levels, can induce excitotoxic effects on oligodendrocytes and, consequently, alterations in myelination processes, inducing alterations in the transmission of GABAergic interneurons that terminate on dopaminergic projections in the striatum [432,433].

Preclinical studies in mice showed that maternal immune activation affects dopamine receptor expression, increasing dopamine levels in the striatum and decreasing D2R levels in adult offspring [434,435]. Alterations in cytokine homeostasis may correlate with the pathogenesis of schizophrenia, probably with a link to alterations in dopaminergic and glutamatergic signaling [435]. IL-1β appears to result in abnormal glutamate release and subsequent neurotransmitter accumulation that induces neuronal death [436]. Studies on rats showed that IL-1β results in the transformation of mesencephalic progenitor cells into a dopaminergic phenotype [437,438], while IL-6 can reduce the survival of serotonergic and dopaminergic neurons [439,440]. Tumor necrosis factor (TNF)-α can cause dopaminergic neurodegeneration with neuronal damage in the striatum [441]. Several cytokines play a role in neurotoxicity and appear to correlate with TRS and potentially to dopaminergic transmission with a pro-inflammatory role: IL-12A that induces an increase in the cytotoxic activity of NK cells and T cells [442,443], IL-18 [444,445], and IL-8 that increases the migration of neutrophils, T lymphocytes, and monocytes [446,447,448], and IL-17 that promotes macrophage and microglia activation [442,449,450]. On the other hand, multiple cytokines exhibit an immunosuppression function: IL-4 increases Th2-mediated cytotoxicity and promotes switching from T-helper to Th2, also modulates macrophage and microglial cell action [451,452], IL-6 modulates the sensitivity of neurons to neurotransmitters [453,454,455], and IL-10 activates JAK1 and STAT3 and induces expression of immunosuppressor genes [442,456,457,458,459]. In addition, TRS patients could have non-dopaminergic mechanisms underlying the disorder, and non-canonical druggable mechanisms could even include neuroinflammatory processes [460].

Dopamine may modulate the activity, migration, differentiation, and proliferation of immune cells [461,462,463,464]. Tetrahydrobiopterin (BH4), a cofactor for tyrosine and dopamine synthesis, can regulate inflammatory cytokines, which in turn modulate the expression of GTPcyclohydrolase 1 (GCH-1), a cofactor for BH4 production [465]. During inflammation, there is a reduction in BH4 synthesis, resulting in decreased dopamine levels; it has been hypothesized that this process is triggered by oxidative processes, ROS, and cytokines (IFN-α, IL-6, and cardiotrophin-1). In contrast, IL-1β, IFN-γ, and TNF-α can increase dopamine levels by increasing BH4 [466,467].

#### 3.5.3. Antipsychotics and Their Potential Anti-Inflammatory Effects

Multiple first- and second-generation antipsychotics may decrease oxidative stress by blocking the release of proinflammatory cytokines from activated microglia [468].

Maes and colleagues demonstrated that patients with TRS had dysregulation cell-mediated immunity with increased monocytic activity [469]. Fernandez-Egea et al. showed that patients affected by schizophrenia have increased numbers of natural killer cells, naïve B cells, memory T cells, and monocytes and decreased numbers of dendritic cells, regulatory T cells, and CD4^+^ T cells in the blood [470]. Typical antipsychotics decrease the plasma levels of IL-6 and IL-6 receptor (IL-6R) [471], while atypical antipsychotics, such as clozapine and risperidone, increase the concentrations of IL-2R, IL-6, and TNF-α [435,472,473]. Clozapine has been demonstrated to interfere with multiple steps of the inflammatory response [474] and inhibit microglial activation [475]. Here, the most relevant anti-inflammatory effects of clozapine are reported: (1) increases IL-6, CC16, and IL-1 receptor antagonist (IL-1Ra) in subjects affected by schizophrenia [472]; (2) inhibits lymphocyte proliferation and production of IL-2, interferon-(IFN)γ, and IL-4 [476]; (3) attenuates the neuroinflammatory response, CD4^+^ T cells of patients treated with clozapine, exhibit an increase in *DRD3* expression [470]; (4) activates the docosahexaenoic acid anti-inflammatory cascade [477]; (5) inhibits Ca^2+/^CaM/Akt-mediated nuclear factor kappa-light-chain-enhancer of activated B cell (NF-κB) [478] and prevents mast cell degranulation in the CNS [479]; (6) regulates cytokine homeostasis [480,481,482,483,484]; and (7) inhibits Akt phosphorylation induced by cytokines released in inflammatory processes [485]. Akt, in turn, can inhibit GSK3 by modulating the level of NMDAR and reducing dopamine concentration in the synapse by increasing DAT activity [422,423]. Finally, lumateperone can modulate serotonergic, dopaminergic, and glutamate neurotransmission (Figure 3), also affecting neuroinflammatory biomarkers by reducing the levels of proinflammatory cytokines such as IL-1b, IL-6, and TNF-α in both the brain and serum [421].

### 3.6. Intracellular Signaling in Schizophrenia: Implication for Dopamine Transmission

A post-mortem study showed that in the PFC of schizophrenia patients, there was an increase in NRG1-induced ErbB4 activation and increased ErbB4/PSD-95 interaction, responsible for the suppression of NMDAR signaling activation [486]. The NRG-ErbB4 signaling pathway regulates dopaminergic and GABAergic transmission and blocking ErbB signaling increases dopamine levels in the striatum [487].

D2R in the striatum may activate the phospholipase C (PLC)/inositol trisphosphate (IP3)/calcineurin signaling pathway and modulate the excitability of GABAergic neurons through the regulation of presynaptic L-type Ca^2+^ channels [488]. Alterations in the regulatory subunits (CNB1 and CNA) of calcineurin can induce changes in vesicle release kinetics through modulation of Ca^2+^ influx from N-type Ca^2+^ channels [489]. Calcineurin is also able to disinhibit protein phosphatase 1 (PP1) [490], which in turn is regulated by dopamine- and cyclic adenosine monophosphate (cAMP)-regulated phosphoprotein, 32 kDa (DARPP-32) strongly expressed in striatal projection neurons [491,492]. DARPP-32 is a cytosolic protein highly expressed in medium spiny neurons of the neostriatum, functioning as an integrator between cortical input and the basal ganglia [493]. Specifically, it has been implicated in schizophrenia proposing its phosphorylation, mediated by dopamine and glutamate signaling, as a potential non-canonical therapeutic target [493]. In addition, preclinical studies on the PFC of schizophrenia animal models have reported decreased levels of DARPP-32 [494], whereas post-mortem clinical studies on the superior temporal gyrus and DLPFC of schizophrenia patients have found a reduced expression [495,496]. In addition, it has demonstrated increased phosphorylation of DARPP-32 during therapy with antipsychotic drugs, such as haloperidol [497,498,499], resulting in improved behavioral performance in animal models. On the other hand, preclinical evidence has shown that D2R can directly activate calcineurin [500], which binds to D1R [501]. These findings suggest the possibility that calcineurin could be a non-canonical target for antipsychotic drugs in patients with schizophrenia [502].

### Antipsychotic Modulation of Dopamine Intracellular Signaling

Multiple lines of evidence have found a link between antipsychotic drugs and epidermal growth factor (EGF) through the signaling of the mitogen-activated protein kinase extracellular signal-regulated kinase (MAPK)-ERK cascade [497,503]. Clozapine induces ERK activation via EGF receptor phosphorylation [504], and it is implicated in ERK1/2 activation, playing a crucial role in synaptogenesis processes and synaptic plasticity [416,417,418,505]. The acute administration of clozapine or haloperidol decreases the activation of proline-rich, extensin-like receptor kinase (pERK)-1 in primary PFC neuronal cultures, whereas clozapine only stimulates pERK1 and pERK2 not affecting the canonical dopamine D2R-G_i/o_-PKA or serotonin 5HT_2A_-G_q_-PLC signaling pathways [504]. Dopamine receptors are G-protein-coupled receptors belonging to two main subfamilies: D1R-like (e.g., D1R and D5R) and D2R-like (e.g., D2R, D3R, and D4R). D1Rs activate cAMP via their coupling to G_s_/G_olf_, whereas D2Rs are bound to G_i/o_ and release Gα_i/o_ and G_βγ_ subunits. Canonically, the function of D2R is implicated in antagonizing cAMP-dependent signaling by inhibiting the activation of PKA [506,507]. In striatal neurons, DARPP-32 is a primary target of PKA and is involved in dopaminergic activity, including the inhibition by D2R of the cAMP-dependent pathway [508]. In this context, β-arrestin is involved in the internalization of the receptor through a complex consisting of Akt, β-arrestin 2, and PP2A phosphatase, demonstrating a key role in the regulation of Akt and GSK [37,390]. Beaulieu and colleagues suggested that the D2R/Akt/β-arrestin pathway might contribute to the dopaminergic dysregulation consistent with the pathophysiology of schizophrenia [390,509]. From a behavioral perspective, genetic KO mice for β-arrestin 2 and GSK reduced acute locomotor response to psychostimulants and exploratory activity [510]. Together, these findings opened new directions for investigating D2R-mediated and cAMP-independent non-canonical intracellular pathways, where alterations in Akt and GSK could be relevant to schizophrenia [509]. On the other hand, dopamine firing was also modulated by serotonin neurotransmission that represents, via 5HT_2A_-G_q_-PLC, the other canonical target of choice for antipsychotics, for second-generation antipsychotics [511]. Through 5HT_2A_ blocking dopamine efflux in all regions of the brain with an excitatory function was facilitated, potentiating dopamine neurotransmission in cortical regions and leading to reduction of dopamine release in the mesolimbic areas that contributes to antipsychotics canonical activity [511].

A preclinical study in the rat cortex and striatum showed that the acute administration of clozapine increased c-Fos levels and induced biphasic phosphorylation of 90 kDa ribosomal s6 kinases (p90RSKs) by MEK/ERK independently of EGF receptor blockade [512]. In contrast, haloperidol and olanzapine resulted in the phosphorylation of p90RSK without ERK signaling [512]. Haloperidol was able to regulate c-Fos expression in the striatum in accordance with the transcriptional regulation of ERK [512]. Clozapine’s properties in modulating the expression of nuclear targets of the ERK cascade, differently from haloperidol and olanzapine, demonstrate a novel signaling pathway that may be relevant as an unconventional mechanism in the treatment of TRS when failure of canonical antipsychotics occurs.

The upregulation of transcripts of the NRG-ErbB signaling pathway in schizophrenia is reported [513], and it has been hypothesized that the abnormal expression of NRG and ErbB4, through the modulation of GABAergic and dopaminergic neurons, may contribute to the onset of schizophrenia is probably via an ErbB4-mediated mechanism exploiting PI3K/Akt signaling [514]. Karbownik and colleagues showed that in glioblastoma cells, and at a dose comparable to ones considered efficacious, clozapine, olanzapine, and quetiapine decreases the mRNA expression of *phosphatidylinositol-4,5-bisphosphate 3-kinase catalytic subunit delta* (*PIK3CD*), a gene encoding for the delta catalytic subunit of PI3K by going on to alter the ErbB4-PI3K pathway [424]. This intracellular signaling has been identified as a possible unconventional molecular target for the treatment of schizophrenia [424]. Liu and coauthors highlighted the correlation between the DRD2-PI3K-AKT signaling cascade and the pathogenesis of schizophrenia [425].

Finally, antipsychotics drugs may regulate the AKT/mTOR pathway by directly affecting dopaminergic signaling: changes in striatal dopamine neurotransmission are caused, at least in part, by elevated D2R expression and upregulated ERK1/2 activation suggesting the implication of mTOR complex 2 (mTORC2) signaling as a non-canonical pathway in regulating striatal dopamine tone and D2R signaling [426].

### 3.7. An Emerging Field: The Repositioning of Antipsychotics in Medicine Implication for Non-Canonical Mechanisms

An ongoing search exploits the possibility of repurposing antipsychotics for the treatment of medical conditions other than psychiatric diseases, such as cancer [515,516,517]. These studies could shed light on new non-canonical MOA of antipsychotics that may be crucial also in the development of novel druggable targets in schizophrenia.

According to this possibility, here we consider non-canonical MOAs of first- and second-generation antipsychotics under evaluation for cancer and other diseases.

Chlorpromazine antagonism at the D2R [518] could play a further role in controlling the growth of certain neoplastic diseases. Chlorpromazine has been identified as a potential candidate for drug repurposing in glioblastoma by inhibiting cell growth and proliferation and controlling mitochondrial mechanisms related to the growth of this type of cancer [515]. This drug induces, in glioma cells, both autophagic cell death via the inhibition of the PI3K/AKT/mTOR pathway [519] and cycle arrest in the G2/M-phase by increasing the expression of the cyclin-dependent kinase (CDK) inhibitor P21 [520]. Colorectal cancer is another type of tumor in which chlorpromazine may have a role in being capable of causing both the upregulation of P53 with subsequent apoptosis and the inhibition of mitotic kinesin KSP/Eg-5 with mitotic arrest [521]. In ovarian cancer, it may induce cell apoptosis by the AKT-dependent activation of GSK3β or B cell lymphoma 2 agonists of cell death (Bad) [522]. Recently, based on the results of preclinical studies, a phase II clinical trial was conducted combining chlorpromazine 50 mg/day to the standard treatment with temozolomide in the sole adjuvant phase of the standard protocol in 41 diagnosed glioblastoma multiforme patients carrying a hypo-methylated *O6-methylguanine-DNA-methyltransferase* (*MGMT*) gene [523]. It was demonstrated that chlorpromazine, along with levomepromazine and thioridazine, promotes cancer stem cell differentiation via the dopamine receptor pathway; these drugs are also involved in the inhibition of mitochondrial DNA polymerase and are responsible for a decrease in ATP production with selective cytotoxicity and antiproliferative activity in leukemic cells [524,525,526,527]. Chlorpromazine appeared to inhibit both the AMPAR [528], relevant for glioblastoma multiforme growth and progression [529], and NMDAR [528] involved in the nesting and growth of brain metastases from breast cancer [530]. A preclinical study for the potential treatment of triple-negative breast cancer and its brain metastases showed that fluphenazine hydrochloride induces downregulation in the expression of cyclin-dependent kinases (CDK) 2, CDK4 and cyclin D1 and the up-regulation of P21 and P27, as well as intrinsic mitochondria-mediated apoptosis in vitro through the induction of G0/G1 cell cycle arrest [531]. Furthermore, fluphenazine hydrochloride is also able to decrease the expression of p44/42 ERK and phosphorylate AKT, involved in the RAS/RAF/MEK/ERK and PI3K/AKT/mTOR pathways, relevant for triple-negative breast cancer, resulting in the suppression of the growth and survival of cancer cells [531]. Considering its antiproliferative effects, in previous years were conducted two clinical trials in 30 patients affected by refractory advanced multiple myeloma to evaluate the safety and tolerability as well as the side effects and best dose of the drug [532,533]. Penfluridol has been explored for the inhibition of cancer metastasis through the action of integrin expression responsible for the suppression of the epithelial-to-mesenchymal transition factors, vimentin, and zinc finger E-box-binding homeobox 1 [534]. The decrease in the levels of integrin α6 and urokinase-type plasminogen activator receptor in glioma cells induced by penfluridol via focal adhesion kinase, paxillin, RAC, and Rho-associated coiled-coil containing kinases proteins activation was found reducing cancer cell migration and invasion [534]. These antiproliferative effects could also be mediated by suppressing cancer growth via the AKT-dependent inhibition of the transcription factor *glioma-associated oncogene 1* [534]. Trifluoperazine acts as a calmodulin antagonist; its involvement in calcium homeostasis has been reported in liver and breast cancer therapy based on the elevated levels of Ca^2+^ and calmodulin typical of these cancers’ cell types, which are associated with increased DNA synthesis and cell proliferation. This drug is found to bind the IP3 (inositol 1,4,5-trisphosphate) receptor, promoting Ca^2+^ release from the endoplasmic reticulum and subsequent cell death [535] and acting as a calmodulin antagonist. It prevents calmodulin–isocitrate dehydrogenase binding that is relevant for the survival and migration of glioblastoma multiforme cells [535]. Finally, trifluoperazine enhanced the effects of doxorubicin on glioma cell growth by increasing the expression of the nuclear tumor suppressor Forkhead box O1 and reducing the levels of multidrug resistance genes [536]. Another example of drug repositioning is represented by pimozide, which through its effect as an antagonist at D2R, D3R, and D4R, has been used to explore the potential anti-metastatic activity in murine melanoma [537]. The epidermal growth factors epiregulin, epigen, and NRG1 are discovered to be downregulated as a result of pimozide’s inhibition of the phosphorylation of signal transducer and activator of transcription (STAT)5 in breast cancer cells [538]. Instead, by targeting the STAT3 signaling pathway, pimozide was found to inhibit proliferation and promote apoptosis in prostate cancer cells [539]. Finally, this drug may weaken the self-renewal and survival of glioblastoma multiforme, inhibit myelin-related infiltration, and increase sensitivity to radiotherapy by acting on the ubiquitin-specific peptidase 1-inhibitor of DNA binding proteins-Nogo ligand1 signaling axis [540,541]. Olanzapine has been reported to have an antiproliferative action and can inhibit glioblastoma cell proliferation, migration, and anchorage-independent growth. Furthermore, it might induce autophagic cell death through the suppression of NF-κB activation [542] and disrupt cholesterol homeostasis, resulting in cell death [543]. Risperidone induces a reduction in PC3 prostate cancer cell proliferation rate in adenocarcinoma [544]. Clomipramine induces the phosphorylation of c-Jun and an increase in cytochrome c release and caspase-3-like activation, leading to the apoptosis of glioblastoma cells [545]. Clozapine seems to have potential adjuvant anticancer activity for the treatment of human breast cancer and metastatic melanoma via the H4 receptor [546,547,548], reducing the growth/survival rates of human astroglioma cells in a dose-dependent manner, as also seen in rat cortical neurons [418] through ErbB kinase inhibition [549].

The potential efficacy of dopamine receptor antagonists, especially at D2R, in cancer therapy, is still in its infancy, and the role of dopamine in cancer biology is worth further exploration [550]. The efficacy of anticancer drugs in breast and colon cancer by dopamine signaling [551], the evidence that polymorphisms of the DR2 modulate the risk of colorectal cancer [552], as well as studies that exhibit a reduction in cancer incidence among schizophrenia patients treated with antipsychotics, add more [553,554,555,556,557,558,559,560].

## 4. Discussion

Although all antipsychotics share the common feature represented by D2R occupancy, which is considered the major mechanism of the “anti-psychotic” effect, in recent years, other cellular mechanisms based on dopaminergic function, its modulation, and non-canonical type have emerged [46,67,174,349,561,562]. Greater precision in defining the molecular and structural nature of dopamine receptors’ interaction with the antipsychotic molecules (e.g., the atomistic molecular dynamics simulations of typical and atypical antipsychotics and the cryoelectronic microscopy structures of D1R-G_s_ and D2R-G_i_ signaling complexes) has also open new avenues to understanding the “dopaminergic side” of antipsychotic action allowing [563,564]. The discovery of different dopaminergic mechanisms at D2R has opened new scenarios of the pharmacological mechanisms, possibly for patients who do not respond or respond poorly to antipsychotics. A relevant change in the canonical conceptualization of dopamine dynamics under antipsychotic exposure is the appreciation of novel presynaptic mechanisms related to dopamine release regulation that is believed to be the major pathogenetic underpinning responsible for hyperdopaminergia in schizophrenia [565]. One of these presynaptic mechanisms is the accumulation of the antipsychotic drug in synaptic vesicles with the endogenous dopamine and its subsequent release upon neuronal activation in extracellular space where the antipsychotic inhibits VGSCs and vesicular exocytosis [78,79]. It has been reported that haloperidol may enhance the activity of VGSCs in cortical neurons in cortical slices after chronic exposure [566], whereas acute haloperidol treatment may induce channel inhibition [567]. It has been suggested that this action upon VGSCs may contribute to time-dependent both therapeutic effects and side effects of haloperidol. A significant effect on VGSCs is also reported for the bias-ligand aripiprazole [568]. Concerning the dopaminergic presynaptic mechanism, DAT has also been implicated for its potential involvement in antipsychotics’ response despite the fact that its role in schizophrenia pathophysiology is still elusive partly due to methodological limitations both at clinical and preclinical levels [5,569,570,571]. Beyond intracellular dopamine D2-directly mediated signaling, dopamine-dependent trans-synaptic mechanisms have emerged, unveiling antipsychotics’ impact on the expression and functions of molecules belonging to different neurotransmitter systems. Among the trans-synaptic effects of antipsychotics, the modulation of the gene expression of glutamatergic PSD proteins has attracted attention for the potential relevance of directly sculpting the synapse architecture based on the pivotal role of PSD proteins in shaping the dendritic spine. It has been demonstrated that PSD proteins, such as PSD-95, Homer, and Shank, are involved in schizophrenia pathophysiology and that their gene expression is modulated by antipsychotics based on the D2R affinity, dose, and duration of treatment, whereas the specific receptor profile beyond dopamine D2R antagonism may further contribute to modulate the pattern of gene expression changes [349]. A critical appraisal of the findings reported in the present systematic review should also consider some limitations and open questions. First, when considering canonical and non-canonical mechanisms of antipsychotics focusing on dopamine, it is probably a reductionistic approach for drugs that have a complex receptor profile. Despite the significant role of other neurotransmitter systems, above all, the glutamatergic [572,573], GABAergic [574], and serotoninergic [82] ones; dopamine remains a pivotal target for schizophrenia treatment [575,576]. Furthermore, the search for dopamine-based non-canonical mechanisms is particularly timing considering the emerging novel concepts on dopamine molecular anatomy [577] and physiology relevant for the pathophysiology and clinics of salience and cognition mechanisms [578], possibly involved in the clinical manifestations of schizophrenia, especially in terms of abnormal cortical-subcortical connectivity [579]. Among these new findings, the repurposing of dopaminergic functional anatomy beyond the classical pathways has been noted [580] and the demonstration of the involvement of nigrostriatal pathways not only in positive symptoms, as already known, but also in cognitive and negative symptoms of psychosis in a more direct way than previously thought [581]. 

Second, it could be questioned if and how non-canonical mechanisms can be pragmatically tackled for the development of novel therapeutic strategies that are needed for those conditions, such as TRS, that do not respond or respond poorly to the available antipsychotics. In this regard and concerning presynaptic dopamine regulation, at least two compounds are in an advanced phase of clinical development represented by TAAR1 agonist [174,582,583,584,585] and xanomeline + trospium [168,169,585,586], both involved in the regulation of dopamine release, though not through a primary dopamine-dependent receptor mechanism. Furthermore, regarding the modulation of synaptic proteins that are a significant non-canonical dopamine-dependent effect of antipsychotics, it should be acknowledged that despite the technical difficulties, there is a growing interest in the possibility of interfering with the PSD proteins’ function. A relevant example is represented by PSD-95 inhibition by *N*PEG4(IETDV)2 (Tat-*N*-dimer), which binds the tandem PDZ1-2 domain of PSD-95 with a high affinity of 4.6 nM [587,588] in the same line of research is the development of the small molecules ZL006 and IC87201 as potential inhibitors of nNOS-PDZ/PSD95-PDZ interactions [589].

Third, an open question of clinical relevance is to what extent novel mechanisms based on non-canonical dopamine-related MOA can be “game-changing” in those difficult-to-treat conditions such as TRS. At present, the xanomelin + trospium combination is under scrutiny in a clinical trial specifically designed for subjects with schizophrenia with an inadequate response to their current antipsychotic treatment [590], and any prediction should be held until the final results are publicly available. With caution, it is conceivable that regulating dopamine release with a non-primary dopamine mechanism could be efficacious for those cases of resistance that emerged after a period of a successful response to antipsychotics’ treatment and believed to be linked, possibly, to the upregulation of D2R (increased B_max_) and/or D2R high affinity increased number.

## 5. Conclusions

Antipsychotics’ dopamine-based non-canonical mechanisms should be exploited (i) for a better understanding of the complex cellular dynamics of antipsychotics beyond D2R occupancy-driven signaling (i.e., cAMP, β-arrestin/AKT and Phospholipase A); (ii) to overcome the limitations, in terms of both efficacy and adverse events of the available compounds. This could allow the exploration of new molecular avenues within the dopamine system, which remains, directly or indirectly, a fundamental pharmacological target in schizophrenia treatment.

## Data Availability

All data are available upon request.

## References

[1] Allikalt A., Purkayastha N., Flad K., Schmidt M.F., Tabor A., Gmeiner P., Hübner H., Weikert D. (2020). Fluorescent ligands for dopamine D2/D3 receptors. Sci. Rep..

[2] Mulvihill K.G. (2018). Presynaptic regulation of dopamine release: Role of the DAT and VMAT2 transporters. Neurochem. Int..

[3] Smigielski L., Wotruba D., Treyer V., Rössler J., Papiol S., Falkai P., Grünblatt E., Walitza S., Rössler W. (2021). The Interplay Between Postsynaptic Striatal D2/3 Receptor Availability, Adversity Exposure and Odd Beliefs: A [11C]-Raclopride PET Study. Schizophr. Bull..

[4] Girgis R.R., Xu X., Miyake N., Easwaramoorthy B., Gunn R.N., A Rabiner E., Abi-Dargham A., Slifstein M. (2010). In Vivo Binding of Antipsychotics to D3 and D2 Receptors: A PET Study in Baboons with [11C]-(+)-PHNO. Neuropsychopharmacology.

[5] Amato D., Vernon A.C., Papaleo F. (2018). Dopamine, the antipsychotic molecule: A perspective on mechanisms underlying antipsychotic response variability. Neurosci. Biobehav. Rev..

[6] Rampino A., Marakhovskaia A., Soares-Silva T., Torretta S., Veneziani F., Beaulieu J.M. (2019). Antipsychotic Drug Responsiveness and Dopamine Receptor Signaling; Old Players and New Prospects. Front. Psychiatry.

[7] Nour M.M., Dahoun T., Schwartenbeck P., Adams R.A., FitzGerald T.H.B., Coello C., Wall M.B., Dolan R.J., Howes O.D. (2018). Dopaminergic basis for signaling belief updates, but not surprise, and the link to paranoia. Proc. Natl. Acad. Sci. USA.

[8] Liloia D., Brasso C., Cauda F., Mancuso L., Nani A., Manuello J., Costa T., Duca S., Rocca P. (2021). Updating and characterizing neuroanatomical markers in high-risk subjects, recently diagnosed and chronic patients with schizophrenia: A revised coordinate-based meta-analysis. Neurosci. Biobehav. Rev..

[9] Allen P., Modinos G., Hubl D., Shields G., Cachia A., Jardri R., Thomas P., Woodward T., Shotbolt P., Plaze M. (2012). Neuroimaging Auditory Hallucinations in Schizophrenia: From Neuroanatomy to Neurochemistry and Beyond. Schizophr. Bull..

[10] Iasevoli F., D’Ambrosio L., Ciccarelli M., Barone A., Gaudieri V., Cocozza S., Pontillo G., Brunetti A., Cuocolo A., de Bartolomeis A. (2022). Altered Patterns of Brain Glucose Metabolism Involve More Extensive and Discrete Cortical Areas in Treatment-resistant Schizophrenia Patients Compared to Responder Patients and Controls: Results From a Head-to-Head 2-[18F]-FDG-PET Study. Schizophr. Bull..

[11] Alnafisah R.S., Reigle J., Eladawi M.A., O’Donovan S.M., Funk A.J., Meller J., Mccullumsmith R.E., Shukla R. (2022). Assessing the effects of antipsychotic medications on schizophrenia functional analysis: A postmortem proteome study. Neuropsychopharmacology.

[12] Reynolds G.P. (2021). The neurochemical pathology of schizophrenia: Post-mortem studies from dopamine to parvalbumin. J. Neural Transm..

[13] Guerrin C.G., Doorduin J., Sommer I.E., de Vries E.F. (2021). The dual hit hypothesis of schizophrenia: Evidence from animal models. Neurosci. Biobehav. Rev..

[14] Howes O.D., McCutcheon R., Agid O., De Bartolomeis A., Van Beveren N.J., Birnbaum M.L., Bloomfield M., Bressan R.A., Buchanan R.W., Carpenter W.T. (2017). Treatment-Resistant Schizophrenia: Treatment Response and Resistance in Psychosis (TRRIP) Working Group Consensus Guidelines on Diagnosis and Terminology. Am. J. Psychiatry.

[15] Page M.J., McKenzie J.E., Bossuyt P.M., Boutron I., Hoffmann T.C., Mulrow C.D., Shamseer L., Tetzlaff J.M., Akl E.A., Brennan S.E. (2021). The PRISMA 2020 statement: An updated guideline for reporting systematic reviews. Syst. Rev..

[16] Howes O., Kapur S. (2009). The Dopamine Hypothesis of Schizophrenia: Version III--The Final Common Pathway. Schizophr. Bull..

[17] Moghaddam B., Javitt D.C. (2011). From Revolution to Evolution: The Glutamate Hypothesis of Schizophrenia and its Implication for Treatment. Neuropsychopharmacology.

[18] Stępnicki P., Kondej M., Kaczor A.A. (2018). Current Concepts and Treatments of Schizophrenia. Molecules.

[19] Cornett E.M., Novitch M., Kaye A.D., Kata V., Kaye A.M. (2017). Medication-Induced Tardive Dyskinesia: A Review and Update. Ochsner J..

[20] Iyo M., Tadokoro S., Kanahara N., Hashimoto T., Niitsu T., Watanabe H., Hashimoto K. (2013). Optimal Extent of Dopamine D2 Receptor Occupancy by Antipsychotics for Treatment of Dopamine Supersensitivity Psychosis and Late-Onset Psychosis. J. Clin. Psychopharmacol..

[21] Tenback D.E., van Harten P.N. (2011). Epidemiology and Risk Factors for (Tardive) Dyskinesia. Int. Rev. Neurobiol..

[22] Kruyer A., Parrilla-Carrero J., Powell C., Brandt L., Gutwinski S., Angelis A., Chalhoub R.M., Jhou T.C., Kalivas P.W., Amato D. (2021). Accumbens D2-MSN hyperactivity drives antipsychotic-induced behavioral supersensitivity. Mol. Psychiatry.

[23] Meiser J., Weindl D., Hiller K. (2013). Complexity of dopamine metabolism. Cell Commun. Signal..

[24] Medina-Hernández V., Ramos-Loyo J., Luquin S., Sánchez L.C., García-Estrada J., Navarro-Ruiz A. (2007). Increased lipid peroxidation and neuron specific enolase in treatment refractory schizophrenics. J. Psychiatr. Res..

[25] Charron A., El Hage C., Servonnet A., Samaha A.-N. (2015). 5-HT2 receptors modulate the expression of antipsychotic-induced dopamine supersensitivity. Eur. Neuropsychopharmacol..

[26] Aldrin-Kirk P., Heuer A., Wang G., Mattsson B., Lundblad M., Parmar M., Björklund T. (2016). DREADD Modulation of Transplanted DA Neurons Reveals a Novel Parkinsonian Dyskinesia Mechanism Mediated by the Serotonin 5-HT6 Receptor. Neuron.

[27] Torrisi S., Laudani S., Contarini G., De Luca A., Geraci F., Managò F., Papaleo F., Salomone S., Drago F., Leggio G. (2020). Dopamine, Cognitive Impairments and Second-Generation Antipsychotics: From Mechanistic Advances to More Personalized Treatments. Pharmaceuticals.

[28] Watson D.J., Loiseau F., Ingallinesi M., Millan M.J., A Marsden C., Fone K.C.F. (2011). Selective Blockade of Dopamine D3 Receptors Enhances while D2 Receptor Antagonism Impairs Social Novelty Discrimination and Novel Object Recognition in Rats: A Key Role for the Prefrontal Cortex. Neuropsychopharmacology.

[29] Truong J.G., Newman A.H., Hanson G.R., Fleckenstein A.E. (2004). Dopamine D2 receptor activation increases vesicular dopamine uptake and redistributes vesicular monoamine transporter-2 protein. Eur. J. Pharmacol..

[30] Remington G., Kapur S., Foussias G., Agid O., Mann S., Borlido C., Richards S., Javaid N. (2012). Tetrabenazine Augmentation in Treatment-Resistant Schizophrenia. J. Clin. Psychopharmacol..

[31] Oda Y., Kanahara N., Iyo M. (2015). Alterations of Dopamine D2 Receptors and Related Receptor-Interacting Proteins in Schizophrenia: The Pivotal Position of Dopamine Supersensitivity Psychosis in Treatment-Resistant Schizophrenia. Int. J. Mol. Sci..

[32] Arinami T. (1997). A functional polymorphism in the promoter region of the dopamine D2 receptor gene is associated with schizophrenia. Hum. Mol. Genet..

[33] Zhang J.-P., Lencz T., Malhotra A.K., Robinson D., Yu J., Gallego J., Fleischhacker W.W., Kahn R.S., Crespo-Facorro B., Vazquez-Bourgon J. (2010). D_2_Receptor Genetic Variation and Clinical Response to Antipsychotic Drug Treatment: A Meta-Analysis. Am. J. Psychiatry.

[34] Oda Y., Tadokoro S., Takase M., Kanahara N., Watanabe H., Shirayama Y., Hashimoto K., Iyo M. (2015). G protein-coupled receptor kinase 6/β-arrestin 2 system in a rat model of dopamine supersensitivity psychosis. J. Psychopharmacol..

[35] Kanahara N., Oda Y., Kimura H., Watanabe H., Hashimoto K., Iyo M. (2015). Genetic association between G protein-coupled receptor kinase 6/β-arrestin 2 and dopamine supersensitivity psychosis in schizophrenia. Neuropsychiatr. Dis. Treat..

[36] Beaulieu J.-M., Gainetdinov R.R. (2011). The Physiology, Signaling, and Pharmacology of Dopamine Receptors. Pharmacol. Rev..

[37] Beaulieu J.-M., Sotnikova T.D., Marion S., Lefkowitz R.J., Gainetdinov R.R., Caron M.G. (2005). An Akt/β-Arrestin 2/PP2A Signaling Complex Mediates Dopaminergic Neurotransmission and Behavior. Cell.

[38] Allen J.A., Yost J.M., Setola V., Chen X., Sassano M.F., Chen M., Peterson S., Yadav P.N., Huang X.-P., Feng B. (2011). Discovery of β-Arrestin–Biased Dopamine D _2_ Ligands for Probing Signal Transduction Pathways Essential for Antipsychotic Efficacy. Proc. Natl. Acad. Sci. USA.

[39] Javitt D.C., Zukin S. (1991). Recent advances in the phencyclidine model of schizophrenia. Am. J. Psychiatry.

[40] Krystal J.H., Karper L.P., Seibyl J.P., Freeman G.K., Delaney R., Bremner J.D., Heninger G.R., Bowers M.B., Charney D.S. (1994). Subanesthetic effects of the noncompetitive NMDA antagonist, ketamine, in humans: Psychotomimetic, perceptual, cognitive, and neuroendocrine responses. Arch. Gen. Psychiatry.

[41] Malhotra A.K., A Pinals D., Weingartner H., Sirocco K., Missar C.D., Pickar D., Breier A. (1996). NMDA receptor function and human cognition: The effects of ketamine in healthy volunteers. Neuropsychopharmacology.

[42] Newcomer J.W., Farber N., Jevtovic-Todorovic V., Selke G., Melson A.K., Hershey T., Craft S., Olney J.W. (1999). Ketamine-Induced NMDA Receptor Hypofunction as a Model of Memory Impairment and Psychosis. Neuropsychopharmacology.

[43] Malhotra A.K., Adler C.M., Kennison S.D., Elman I., Pickar D., Breier A. (1997). Clozapine Blunts N-Methyl-d-Aspartate Antagonist-Induced Psychosis: A Study with Ketamine. Biol. Psychiatry.

[44] Lahti A.C., A Weiler M., Tamara M., Parwani A., A Tamminga C. (2001). Effects of Ketamine in Normal and Schizophrenic Volunteers. Neuropsychopharmacology.

[45] Seeman P. (2013). Are dopamine D2 receptors out of control in psychosis?. Prog. Neuro-Psychopharmacol. Biol. Psychiatry.

[46] De Bartolomeis A., Vellucci L., Barone A., Manchia M., De Luca V., Iasevoli F., Correll C.U. (2022). Clozapine’s multiple cellular mechanisms: What do we know after more than fifty years? A systematic review and critical assessment of translational mechanisms relevant for innovative strategies in treatment-resistant schizophrenia. Pharmacol. Ther..

[47] Schrader J.M., Irving C.M., Octeau J.C., Christian J.A., Aballo T.J., Kareemo D.J., Conti J., Camberg J.L., Lane J.R., Javitch J.A. (2019). The differential actions of clozapine and other antipsychotic drugs on the translocation of dopamine D2 receptors to the cell surface. J. Biol. Chem..

[48] Kiss B., Horváth A., Némethy Z., Schmidt É., Laszlovszky I., Bugovics G., Fazekas K., Hornok K., Orosz S., Gyertyán I. (2010). Cariprazine (RGH-188), a Dopamine D_3_ Receptor-Preferring, D_3_/D_2_ Dopamine Receptor Antagonist–Partial Agonist Antipsychotic Candidate: In Vitro and Neurochemical Profile. Experiment.

[49] Németh G., Laszlovszky I., Czobor P., Szalai E., Szatmári B., Harsányi J., Barabássy Á., Debelle M., Durgam S., Bitter I. (2017). Cariprazine versus risperidone monotherapy for treatment of predominant negative symptoms in patients with schizophrenia: A randomised, double-blind, controlled trial. Lancet.

[50] Németh B., Molnár A., Akehurst R., Horváth M., Kóczián K., Németh G., Götze Á., Vokó Z., Lane H.-Y., Lee C.-C. (2017). Quality-adjusted life year difference in patients with predominant negative symptoms of schizophrenia treated with cariprazine and risperidone. J. Comp. Eff. Res..

[51] Leriche L., Schwartz J.-C., Sokoloff P. (2003). The dopamine D3 receptor mediates locomotor hyperactivity induced by NMDA receptor blockade. Neuropharmacology.

[52] Sun X., Gou H.-Y., Li F., Lu G.-Y., Song R., Yang R.-F., Wu N., Su R.-B., Cong B., Li J. (2016). Y-QA31, a novel dopamine D3 receptor antagonist, exhibits antipsychotic-like properties in preclinical animal models of schizophrenia. Acta Pharmacol. Sin..

[53] Scharfetter J., Chaudhry H.R., Hornik K., Fuchs K., Sieghart W., Kasper S., Aschauer H.N. (1999). Dopamine D3 receptor gene polymorphism and response to clozapine in schizophrenic Pakistani patients. Eur. Neuropsychopharmacol..

[54] Nakajima S., Gerretsen P., Takeuchi H., Caravaggio F., Chow T., Le Foll B., Mulsant B., Pollock B., Graff-Guerrero A. (2013). The potential role of dopamine D3 receptor neurotransmission in cognition. Eur. Neuropsychopharmacol..

[55] Leggio G.M., Torrisi S.A., Mastrogiacomo R., Mauro D., Chisari M., Devroye C., Scheggia D., Nigro M., Geraci F., Pintori N. (2019). The epistatic interaction between the dopamine D3 receptor and dysbindin-1 modulates higher-order cognitive functions in mice and humans. Mol. Psychiatry.

[56] Kiss B., Laszlovszky I., Krámos B., Visegrády A., Bobok A., Lévay G., Lendvai B., Román V. (2021). Neuronal Dopamine D3 Receptors: Translational Implications for Preclinical Research and CNS Disorders. Biomolecules.

[57] Gonzalez-Burgos G., Cho R.Y., Lewis D.A. (2015). Alterations in Cortical Network Oscillations and Parvalbumin Neurons in Schizophrenia. Biol. Psychiatry.

[58] Uhlhaas P.J., Singer W. (2010). Abnormal neural oscillations and synchrony in schizophrenia. Nat. Rev. Neurosci..

[59] Kulkarni S.K., Ninan I. (2000). Dopamine D_4_receptors and development of newer antipsychotic drugs. Fundam. Clin. Pharmacol..

[60] Mrzljak L., Bergson C., Pappy M., Huff R., Levenson R., Goldman-Rakic P.S. (1996). Localization of dopamine D4 receptors in GABAergic neurons of the primate brain. Nature.

[61] Wang Y., Jin Y.-K., Guo T.-C., Li Z.-R., Feng B.-Y., Han J.-H., Vreugdenhil M., Lu C.-B. (2022). Activation of Dopamine 4 Receptor Subtype Enhances Gamma Oscillations in Hippocampal Slices of Aged Mice. Front. Aging Neurosci..

[62] Andersson R.H., Johnston A., Herman P.A., Winzer-Serhan U.H., Karavanova I., Vullhorst D., Fisahn A., Buonanno A. (2012). Neuregulin and dopamine modulation of hippocampal gamma oscillations is dependent on dopamine D4 receptors. Proc. Natl. Acad. Sci. USA.

[63] Roth B.L., Tandra S., Burgess L.H., Sibley D.R., Meltzer H.Y. (1995). D4 dopamine receptor binding affinity does not distinguish between typical and atypical antipsychotic drugs. Psychopharmacology.

[64] Prus A.J., Wise L.E., Pehrson A.L., Philibin S.D., Bang-Andersen B., Arnt J., Porter J.H. (2016). Discriminative stimulus properties of 1.25 mg/kg clozapine in rats: Mediation by serotonin 5-HT 2 and dopamine D 4 receptors. Brain Res..

[65] Rajagopal V., Sundaresan L., Rajkumar A.P., Chittybabu C., Kuruvilla A., Srivastava A., Balasubramanian P., Jacob K.S., Jacob M. (2014). Genetic association between the DRD4 promoter polymorphism and clozapine-induced sialorrhea. Psychiatr. Genet..

[66] Brisch R., Saniotis A., Wolf R., Bielau H., Bernstein H.-G., Steiner J., Bogerts B., Braun A.K., Jankowski Z.P., Ekumaratilake J. (2014). The Role of Dopamine in Schizophrenia from a Neurobiological and Evolutionary Perspective: Old Fashioned, but Still in Vogue. Front. Psychiatry.

[67] McCarthy C.I., Mustafá E.R., Cornejo M.P., Yaneff A., Rodríguez S.S., Perello M., Raingo J. (2023). Chlorpromazine, an Inverse Agonist of D1R-Like, Differentially Targets Voltage-Gated Calcium Channel (CaV) Subtypes in mPFC Neurons. Mol. Neurobiol..

[68] McCarthy C.I., Chou-Freed C., Rodríguez S.S., Yaneff A., Davio C., Raingo J. (2020). Constitutive activity of dopamine receptor type 1 (D1R) increases CaV2.2 currents in PFC neurons. J. Gen. Physiol..

[69] Glass M.J., Robinson D.C., Waters E., Pickel V.M. (2013). Deletion of the NMDA-NR1 receptor subunit gene in the mouse nucleus accumbens attenuates apomorphine-induced dopamine D1 receptor trafficking and acoustic startle behavior. Synapse.

[70] Lynch M.R. (1992). Schizophrenia and the D1 receptor: Focus on negative symptoms. Prog. Neuro-Psychopharmacol. Biol. Psychiatry.

[71] Wenthur C.J., Lindsley C.W. (2013). Classics in Chemical Neuroscience: Clozapine. ACS Chem. Neurosci..

[72] Chou Y.-H., Halldin C., Farde L. (2006). Clozapine binds preferentially to cortical D1-like dopamine receptors in the primate brain: A PET study. Psychopharmacology.

[73] Abekawa T., Ito K., Koyama T. (2006). Role of the simultaneous enhancement of NMDA and dopamine D1 receptor-mediated neurotransmission in the effects of clozapine on phencyclidine-induced acute increases in glutamate levels in the rat medial prefrontal cortex. Naunyn-Schmiedeberg’s Arch. Pharmacol..

[74] Karlsson P., Farde L., Härnryd C., Sedvall G., Smith L., Wiesel F.-A. (1995). Lack of apparent antipsychotic effect of the D1-dopamine recepotr antagonist SCH39166 in acutely ill schizophrenic patients. Psychopharmacology.

[75] Faron-Górecka A., Górecki A., Kuśmider M., Wasylewski Z., Dziedzicka-Wasylewska M. (2008). The role of D1–D2 receptor hetero-dimerization in the mechanism of action of clozapine. Eur. Neuropsychopharmacol..

[76] Perreault M.L., Hasbi A., Alijaniaram M., Fan T., Varghese G., Fletcher P.J., Seeman P., O’Dowd B.F., George S.R. (2010). The Dopamine D1-D2 Receptor Heteromer Localizes in Dynorphin/Enkephalin Neurons: Increased High Affinity State Following amphetamine and in Schizophrenia. J. Biol. Chem..

[77] Hasbi A., O’Dowd B.F., George S.R. (2011). Dopamine D1-D2 receptor heteromer signaling pathway in the brain: Emerging physiological relevance. Mol. Brain.

[78] Tischbirek C.H., Wenzel E.M., Zheng F., Huth T., Amato D., Trapp S., Denker A., Welzel O., Lueke K., Svetlitchny A. (2012). Use-Dependent Inhibition of Synaptic Transmission by the Secretion of Intravesicularly Accumulated Antipsychotic Drugs. Neuron.

[79] Chestnykh D.A., Amato D., Kornhuber J., Müller C.P. (2021). Pharmacotherapy of schizophrenia: Mechanisms of antipsychotic accumulation, therapeutic action and failure. Behav. Brain Res..

[80] Takano A., Suhara T., Ikoma Y., Yasuno F., Maeda J., Ichimiya T., Sudo Y., Inoue M., Okubo Y. (1999). Estimation of the time-course of dopamine D2 receptor occupancy in living human brain from plasma pharmacokinetics of antipsychotics. Int. J. Neuropsychopharmacol..

[81] Morton A., Cousin M.A. (2012). The Best Things Come in Small Packages—Vesicular Delivery of Weak Base Antipsychotics. Neuron.

[82] Schmidt C.J., Sorensen S.M., Kenne J.H., Carr A.A., Palfreyman M.G. (1995). The role of 5-HT2A receptors in antipsychotic activity. Life Sci..

[83] Borroto-Escuela D.O., Romero-Fernandez W., Narvaez M., Oflijan J., Agnati L.F., Fuxe K. (2013). Hallucinogenic 5-HT2AR agonists LSD and DOI enhance dopamine D2R protomer recognition and signaling of D2-5-HT2A heteroreceptor complexes. Biochem. Biophys. Res. Commun..

[84] Łukasiewicz S., Polit A., Kędracka-Krok S., Wędzony K., Maćkowiak M., Dziedzicka-Wasylewska M. (2010). Hetero-dimerization of serotonin 5-HT2A and dopamine D2 receptors. Biochim. Biophys. Acta (BBA) Mol. Cell Res..

[85] Borroto-Escuela D.O., Romero-Fernandez W., Tarakanov A.O., Marcellino D., Ciruela F., Agnati L.F., Fuxe K. (2010). Dopamine D2 and 5-hydroxytryptamine 5-HT2A receptors assemble into functionally interacting heteromers. Biochem. Biophys. Res. Commun..

[86] González-Maeso J., Ang R.L., Yuen T., Chan P., Weisstaub N.V., López-Giménez J.F., Zhou M., Okawa Y., Callado L.F., Milligan G. (2008). Identification of a serotonin/glutamate receptor complex implicated in psychosis. Nature.

[87] Moreno J.L., Muguruza C., Umali A., Mortillo S., Holloway T., Pilar-Cuéllar F., Mocci G., Seto J., Callado L.F., Neve R.L. (2012). Identification of Three Residues Essential for 5-Hydroxytryptamine 2A-Metabotropic Glutamate 2 (5-HT2A·mGlu2) Receptor Heteromerization and Its Psychoactive Behavioral Function. J. Biol. Chem..

[88] Fribourg M., Moreno J.L., Holloway T., Provasi D., Baki L., Mahajan R., Park G., Adney S.K., Hatcher C., Eltit J.M. (2011). Decoding the Signaling of a GPCR Heteromeric Complex Reveals a Unifying Mechanism of Action of Antipsychotic Drugs. Cell.

[89] Barnes N.M., Sharp T. (1999). A review of central 5-HT receptors and their function. Neuropharmacology.

[90] Mylecharane E. (1995). Ventral tegmental area 5-HT receptors: Mesolimbic dopamine release and behavioural studies. Behav. Brain Res..

[91] Wang R.Y., Ashby C.R., Edwards E., Zhang J.Y. (1994). The role of 5-HT3-like receptors in the action of clozapine. J. Clin. Psychiatry.

[92] Ji X., Takahashi N., Saito S., Ishihara R., Maeno N., Inada T., Ozaki N. (2008). Relationship between three serotonin receptor subtypes (HTR3A, HTR2A and HTR4) and treatment-resistant schizophrenia in the Japanese population. Neurosci. Lett..

[93] A Dawson L., Nguyen H.Q., Li P. (2003). Potentiation of amphetamine-induced changes in dopamine and 5-HT by a 5-HT6 receptor antagonist. Brain Res. Bull..

[94] Song X., Jensen M., Jogini V., Stein R.A., Lee C.-H., Mchaourab H.S., Shaw D.E., Gouaux E. (2018). Mechanism of NMDA receptor channel block by MK-801 and memantine. Nature.

[95] De Bruin N., van Drimmelen M., Kops M., van Elk J., de Wetering M.M.-V., Schwienbacher I. (2013). Effects of risperidone, clozapine and the 5-HT6 antagonist GSK-742457 on PCP-induced deficits in reversal learning in the two-lever operant task in male Sprague Dawley rats. Behav. Brain Res..

[96] Rodefer J.S., Nguyen T.N., Karlsson J.-J., Arnt J. (2007). Reversal of Subchronic PCP-Induced Deficits in Attentional Set Shifting in Rats by Sertindole and a 5-HT6 Receptor Antagonist: Comparison Among Antipsychotics. Neuropsychopharmacology.

[97] Nikiforuk A., Kos T., Fijał K., Hołuj M., Rafa D., Popik P. (2013). Effects of the Selective 5-HT7 Receptor Antagonist SB-269970 and Amisulpride on Ketamine-Induced Schizophrenia-like Deficits in Rats. PLoS ONE.

[98] Darmani N.A., Martin B.R., Pandey U., Glennon R.A. (1990). Do functional relationships exist between 5-HT1A and 5-HT2 receptors?. Pharmacol. Biochem. Behav..

[99] Blier P., Ward N.M. (2003). Is there a role for 5-HT1A agonists in the treatment of depression?. Biol. Psychiatry.

[100] Feighner J.P., Boyer W.F. (1989). Serotonin-IA Anxiolytics: An Overview. Psychopathology.

[101] Pucadyil T.J., Kalipatnapu S., Chattopadhyay A. (2005). The Serotonin1A A Receptor: A Representative Member of the Serotonin Receptor Family. Cell. Mol. Neurobiol..

[102] Naidu P.S., Kulkarni S.K. (2001). Effect of 5-HT1A and 5-HT2A/2C receptor modulation on neuroleptic-induced vacuous chewing movements. Eur. J. Pharmacol..

[103] Zazpe A., Artaiz I., Innerárity A., del Olmo E., Castro E., Labeaga L., Pazos A., Orjales A. (2006). In vitro and in vivo characterization of F-97013-GD, a partial 5-HT1A agonist with antipsychotic- and antiparkinsonian-like properties. Neuropharmacology.

[104] Meltzer H.Y., Sumiyoshi T. (2008). Does stimulation of 5-HT1A receptors improve cognition in schizophrenia?. Behav. Brain Res..

[105] Schreiber R., Newman-Tancredi A. (2014). Improving cognition in schizophrenia with antipsychotics that elicit neurogenesis through 5-HT1A receptor activation. Neurobiol. Learn. Mem..

[106] Plesnicar B., Terzic T., Kastelic M., Dolzan V. (2015). Influence of 5-HT1A and 5-HTTLPR genetic variants on the schizophrenia symptoms and occurrence of treatment-resistant schizophrenia. Neuropsychiatr. Dis. Treat..

[107] Tejedor-Real P., Vogel R., Mallet J., Biguet N.F. (2005). Gi/Go protein-dependent presynaptic mechanisms are involved in clozapine-induced down-regulation of tyrosine hydroxylase in PC12 cells. J. Neurosci. Res..

[108] Benes F.M. (1991). Deficits in Small Interneurons in Prefrontal and Cingulate Cortices of Schizophrenic and Schizoaffective Patients. Arch. Gen. Psychiatry.

[109] Abekawa T., Ito K., Nakagawa S., Koyama T. (2007). Prenatal exposure to an NMDA receptor antagonist, MK-801 reduces density of parvalbumin-immunoreactive GABAergic neurons in the medial prefrontal cortex and enhances phencyclidine-induced hyperlocomotion but not behavioral sensitization to methamphetamine in postpubertal rats. Psychopharmacology.

[110] Fatemi S.H., Folsom T.D., Thuras P.D. (2011). Deficits in GABAB receptor system in schizophrenia and mood disorders: A postmortem study. Schizophr. Res..

[111] Orhan F., Fatouros-Bergman H., Goiny M., Malmqvist A., Piehl F., Cervenka S., Collste K., Victorsson P., Sellgren C.M., Karolinska Schizophrenia Project (KaSP) Consortium (2017). CSF GABA is reduced in first-episode psychosis and associates to symptom severity. Mol. Psychiatry.

[112] Akbarian S. (1995). Gene Expression for Glutamic Acid Decarboxylase Is Reduced Without Loss of Neurons in Prefrontal Cortex of Schizophrenics. Arch. Gen. Psychiatry.

[113] Chung D.W., Fish K.N., Lewis D.A. (2016). Pathological Basis for Deficient Excitatory Drive to Cortical Parvalbumin Interneurons in Schizophrenia. Am. J. Psychiatry.

[114] Ishikawa M., Mizukami K., Iwakiri M., Asada T. (2005). Immunohistochemical and immunoblot analysis of γ-aminobutyric acid B receptor in the prefrontal cortex of subjects with schizophrenia and bipolar disorder. Neurosci. Lett..

[115] Mizukami K., Sasaki M., Ishikawa M., Iwakiri M., Hidaka S., Shiraishi H., Iritani S. (2000). Immunohistochemical localization of γ-aminobutyric acidB receptor in the hippocampus of subjects with schizophrenia. Neurosci. Lett..

[116] Miyazawa A., Kanahara N., Kogure M., Otsuka I., Okazaki S., Watanabe Y., Yamasaki F., Nakata Y., Oda Y., Hishimoto A. (2021). A preliminary genetic association study of GAD1 and GABAB receptor genes in patients with treatment-resistant schizophrenia. Mol. Biol. Rep..

[117] Adler L.E., Olincy A., Cawthra E.M., McRae K.A., Harris J.G., Nagamoto H.T., Waldo M.C., Hall M.-H., Bowles A., Woodward L. (2004). Varied Effects of Atypical Neuroleptics on P50 Auditory Gating in Schizophrenia Patients. Am. J. Psychiatry.

[118] Freedman R., Adams C., Adler L.E., Bickford P.C., Gault J., Harris J.G., Nagamoto H.T., Olincy A., Ross R.G., Stevens K.E. (2000). Inhibitory neurophysiological deficit as a phenotype for genetic investigation of schizophrenia. Am. J. Med. Genet..

[119] Miyazawa A., Kanahara N., Nakata Y., Kodama S., Kimura H., Kimura A., Oda Y., Watanabe H., Iyo M. (2021). Clozapine Prolongs Cortical Silent Period in Patients with Treatment-Resistant Schizophrenia. Psychopharmacol. Bull..

[120] Kaster T.S., de Jesus D., Radhu N., Farzan F., Blumberger D.M., Rajji T.K., Fitzgerald P., Daskalakis Z.J. (2015). Clozapine potentiation of GABA mediated cortical inhibition in treatment resistant schizophrenia. Schizophr. Res..

[121] Daskalakis Z.J., George T.P. (2009). Clozapine, GABAB, and the Treatment of Resistant Schizophrenia. Clin. Pharmacol. Ther..

[122] Nair P.C., McKinnon R.A., Miners J.O., Bastiampillai T. (2020). Binding of clozapine to the GABAB receptor: Clinical and structural insights. Mol. Psychiatry.

[123] Ueno F., Nakajima S., Iwata Y., Honda S., Torres-Carmona E., Mar W., Tsugawa S., Truong P., Plitman E., Noda Y. (2022). Gamma-aminobutyric acid (GABA) levels in the midcingulate cortex and clozapine response in patients with treatment-resistant schizophrenia: A proton magnetic resonance spectroscopy ( ^1^ H-MRS) study. Psychiatry Clin. Neurosci..

[124] Nakahara T., Tsugawa S., Noda Y., Ueno F., Honda S., Kinjo M., Segawa H., Hondo N., Mori Y., Watanabe H. (2021). Glutamatergic and GABAergic metabolite levels in schizophrenia-spectrum disorders: A meta-analysis of 1H-magnetic resonance spectroscopy studies. Mol. Psychiatry.

[125] Gluck M.R., Thomas R.G., Davis K.L., Haroutunian V. (2002). Implications for Altered Glutamate and GABA Metabolism in the Dorsolateral Prefrontal Cortex of Aged Schizophrenic Patients. Am. J. Psychiatry.

[126] Burbaeva G.S., Boksha I.S., Turishcheva M.S., Vorobyeva E.A., Savushkina O.K., Tereshkina E.B. (2003). Glutamine synthetase and glutamate dehydrogenase in the prefrontal cortex of patients with schizophrenia. Prog. Neuro-Psychopharmacol. Biol. Psychiatry.

[127] Mei Y.-Y., Wu D.C., Zhou N. (2018). Astrocytic Regulation of Glutamate Transmission in Schizophrenia. Front. Psychiatry.

[128] Erecińska M., Silver I.A. (1990). Metabolism and role of glutamate in mammalian brain. Prog. Neurobiol..

[129] Walton H.S., Dodd P.R. (2007). Glutamate–glutamine cycling in Alzheimer’s disease. Neurochem. Int..

[130] Madeira C., Alheira F.V., Calcia M., Silva T.C.S., Tannos F.M., Vargas-Lopes C., Fisher M., Goldenstein N., Brasil M.A., Vinogradov S. (2018). Blood Levels of Glutamate and Glutamine in Recent Onset and Chronic Schizophrenia. Front. Psychiatry.

[131] Marques T.R., Ashok A.H., Angelescu I., Borgan F., Myers J., Lingford-Hughes A., Nutt D.J., Veronese M., Turkheimer F.E., Howes O.D. (2020). GABA-A receptor differences in schizophrenia: A positron emission tomography study using [11C]Ro154513. Mol. Psychiatry.

[132] Lu C., Zhu X., Feng Y., Ao W., Li J., Gao Z., Luo H., Chen M., Cai F., Zhan S. (2023). Atypical antipsychotics antagonize GABAA receptors in the ventral tegmental area GABA neurons to relieve psychotic behaviors. Mol. Psychiatry.

[133] Svensson T.H. (2003). α-Adrenoceptor modulation hypothesis of antipsychotic atypicality. Prog. Neuro-Psychopharmacol. Biol. Psychiatry.

[134] Stuchlík A., Petrásek T., Valeš K. (2009). Effect of alpha1-adrenergic antagonist prazosin on behavioral alterations induced by MK-801 in a spatial memory task in Long-Evans rats. Physiol. Res..

[135] Sallinen J., Höglund I., Engström M., Lehtimäki J., Virtanen R., Sirviö J., Wurster S., Savola J.-M., Haapalinna A. (2007). Pharmacological characterization and CNS effects of a novel highly selective α_2C_-adrenoceptor antagonist JP-1302. Br. J. Pharmacol..

[136] Uys M., Shahid M., Sallinen J., Dreyer W., Cockeran M., Harvey B.H. (2016). The α2C-adrenoceptor antagonist, ORM-10921, has antipsychotic-like effects in social isolation reared rats and bolsters the response to haloperidol. Prog. Neuro-Psychopharmacol. Biol. Psychiatry.

[137] Franowicz J.S., Kessler L.E., Borja C.M.D., Kobilka B.K., Limbird L.E., Arnsten A.F.T. (2002). Mutation of the α_2A_-Adrenoceptor Impairs Working Memory Performance and Annuls Cognitive Enhancement by Guanfacine. J. Neurosci..

[138] Dutra R.C., Andreazza A.P., Andreatini R., Tufik S., Vital M.A. (2002). Behavioral effects of MK-801 on reserpine-treated mice. Prog. Neuro-Psychopharmacol. Biol. Psychiatry.

[139] Ramos B.P., Arnsten A.F. (2007). Adrenergic pharmacology and cognition: Focus on the prefrontal cortex. Pharmacol. Ther..

[140] Correll C.U., Schooler N.R. (2020). Negative Symptoms in Schizophrenia: A Review and Clinical Guide for Recognition, Assessment, and Treatment. Neuropsychiatr. Dis. Treat..

[141] Marcus M.M., Wiker C., Frånberg O., Konradsson-Geuken A., Langlois X., Jardemark K., Svensson, T.H. (2009). Adjunctive α2-adrenoceptor blockade enhances the antipsychotic-like effect of risperidone and facilitates cortical dopaminergic and glutamatergic, NMDA receptor-mediated transmission. Int. J. Neuropsychopharmacol..

[142] Ballmaier M., Zoli M., Mazzoncini R., Gennarelli M., Spano P., Spano P. (2001). Combined α2-adrenergic/D2 dopamine receptor blockade fails to reproduce the ability of clozapine to reverse phencyclidine-induced deficits in prepulse inhibition of startle. Psychopharmacology.

[143] Litman R.E., Su T.-P., Potter W.Z., Hong W.W., Pickar D. (1996). Idazoxan and Response to Typical Neuroleptics in Treatment-Resistant Schizophrenia. Br. J. Psychiatry.

[144] E Litman R., Hong W.W., Weissman E.M., Su T.P., Potter W.Z., Pickar D. (1993). Idazoxan, an alpha 2 antagonist, augments fluphenazine in schizophrenic patients: A pilot study. J. Clin. Psychopharmacol..

[145] Bellingham M.C. (2011). A Review of the Neural Mechanisms of Action and Clinical Efficiency of Riluzole in Treating Amyotrophic Lateral Sclerosis: What have we Learned in the Last Decade?. CNS Neurosci. Ther..

[146] Doble A. (1996). The pharmacology and mechanism of action of riluzole. Neurology.

[147] Snyder G.L., Vanover K.E., Zhu H., Miller D.B., O’Callaghan J.P., Tomesch J., Li P., Zhang Q., Krishnan V., Hendrick J.P. (2014). Functional profile of a novel modulator of serotonin, dopamine, and glutamate neurotransmission. Psychopharmacology.

[148] E Shannon H., Bymaster F.P., O Calligaro D., Greenwood B., Mitch C.H., Sawyer B.D., Ward J.S., Wong D.T., Olesen P.H., Sheardown M.J. (1994). Xanomeline: A novel muscarinic receptor agonist with functional selectivity for M1 receptors. Experiment.

[149] Crook J.M., Dean B., Pavey G., Copolov D. (1999). The binding of [3H]AF-DX 384 is reduced in the caudate-putamen of subjects with schizophrenia. Life Sci..

[150] Crook J.M., Tomaskovic-Crook E., Copolov D.L., Dean B. (2000). Decreased muscarinic receptor binding in subjects with schizophrenia: A study of the human hippocampal formation. Biol. Psychiatry.

[151] Crook J.M., Tomaskovic-Crook E., Copolov D.L., Dean B. (2001). Low Muscarinic Receptor Binding in Prefrontal Cortex From Subjects With Schizophrenia: A Study of Brodmann’s Areas 8, 9, 10, and 46 and the Effects of Neuroleptic Drug Treatment. Am. J. Psychiatry.

[152] Dean B., Thomas N., Lai C.-Y., Chen W.J., Scarr E. (2015). Changes in cholinergic and glutamatergic markers in the striatum from a sub-set of subjects with schizophrenia. Schizophr. Res..

[153] Prus A.J., Pehrson A.L., Philibin S.D., Wood J.T., Vunck S.A., Porter J.H. (2008). The role of M1 muscarinic cholinergic receptors in the discriminative stimulus properties of N-desmethylclozapine and the atypical antipsychotic drug clozapine in rats. Psychopharmacology.

[154] Carruthers S.P., Gurvich C.T., Rossell S.L. (2015). The muscarinic system, cognition and schizophrenia. Neurosci. Biobehav. Rev..

[155] Malkoff A., Weizman A., Gozes I., Rehavi M. (2008). Decreased M1 muscarinic receptor density in rat amphetamine model of schizophrenia is normalized by clozapine, but not haloperidol. J. Neural Transm..

[156] Sur C., Mallorga P.J., Wittmann M., Jacobson M.A., Pascarella D., Williams J.B., Brandish P.E., Pettibone D.J., Scolnick E.M., Conn P.J. (2003). N-desmethylclozapine, an allosteric agonist at muscarinic 1 receptor, potentiates N-methyl-d-aspartate receptor activity. Proc. Natl. Acad. Sci. USA.

[157] Ghoshal A., Rook J.M., Dickerson J.W., Roop G.N., Morrison R.D., Jalansakrikar N., Lamsal A., Noetzel M.J., Poslusney M.S., Wood M.R. (2015). Potentiation of M1 Muscarinic Receptor Reverses Plasticity Deficits and Negative and Cognitive Symptoms in a Schizophrenia Mouse Model. Neuropsychopharmacology.

[158] Weston-Green K., Huang X.-F., Deng C. (2013). Second Generation Antipsychotic-Induced Type 2 Diabetes: A Role for the Muscarinic M3 Receptor. CNS Drugs.

[159] de Azua I.R., Gautam D., Guettier J.-M., Wess J. (2011). Novel insights into the function of β-cell M3 muscarinic acetylcholine receptors: Therapeutic implications. Trends Endocrinol. Metab..

[160] De Luca V., Wang H., Squassina A., Wong G.W., Yeomans J., Kennedy J.L. (2004). Linkage of M5 Muscarinic and α7-Nicotinic Receptor Genes on 15q13 to Schizophrenia. Neuropsychobiology.

[161] Thomsen M., Wörtwein G., Fink-Jensen A., Woldbye D.P.D., Wess J., Caine S.B. (2007). Decreased prepulse inhibition and increased sensitivity to muscarinic, but not dopaminergic drugs in M5 muscarinic acetylcholine receptor knockout mice. Psychopharmacology.

[162] Galloway C.R., Lebois E.P., Shagarabi S.L., Hernandez N.A., Manns J.R. (2014). Effects of Selective Activation of M1 and M4 Muscarinic Receptors on Object Recognition Memory Performance in Rats. Pharmacology.

[163] Tzavara E.T., Bymaster F.P., Davis R.J., Wade M.R., Perry K.W., Wess J., McKinzie D.L., Felder C., Nomikos G.G. (2004). M _4_ muscarinic receptors regulate the dynamics of cholinergic and dopaminergic neurotransmission: Relevance to the pathophysiology and treatment of related central nervous system pathologies. FASEB J..

[164] E Shannon H., Hart J.C., Bymaster F.P., O Calligaro D., DeLapp N.W., Mitch C.H., Ward J.S., Fink-Jensen A., Sauerberg P., Jeppesen L. (1999). Muscarinic receptor agonists, like dopamine receptor antagonist antipsychotics, inhibit conditioned avoidance response in rats. Experiment.

[165] Shannon H.E., Rasmussen K., Bymaster F.P., Hart J.C., Peters S.C., Swedberg M.D., Jeppesen L., Sheardown M.J., Sauerberg P., Fink-Jensen A. (2000). Xanomeline, an M1/M4 preferring muscarinic cholinergic receptor agonist, produces antipsychotic-like activity in rats and mice. Schizophr. Res..

[166] Thomsen M., Wess J., Fulton B.S., Fink-Jensen A., Caine S.B. (2009). Modulation of prepulse inhibition through both M1 and M4 muscarinic receptors in mice. Psychopharmacology.

[167] Watson J., Brough S., Coldwell M.C., Gager T., Ho M., Hunter A.J., Jerman J., Middlemiss D.N., Riley G.J., Brown A.M. (1998). Functional effects of the muscarinic receptor agonist, xanomeline, at 5-HT_1_and 5-HT_2_receptors. Br. J. Pharmacol..

[168] Brannan S.K., Sawchak S., Miller A.C., Lieberman J.A., Paul S.M., Breier A. (2021). Muscarinic Cholinergic Receptor Agonist and Peripheral Antagonist for Schizophrenia. N. Engl. J. Med..

[169] Weiden P.J., Breier A., Kavanagh S., Miller A.C., Brannan S.K., Paul S.M. (2022). Antipsychotic Efficacy of KarXT (Xanomeline−Trospium). J. Clin. Psychiatry.

[170] Lavrador M., Castel-Branco M.M., Cabral A.C., Veríssimo M.T., Figueiredo I.V., Fernandez-Llimos F. (2020). Association between anticholinergic burden and anticholinergic adverse outcomes in the elderly: Pharmacological basis of their predictive value for adverse outcomes. Pharmacol. Res..

[171] Lieberman J.A. (2004). Managing anticholinergic side effects. Prim. Care Companion J. Clin. Psychiatry.

[172] Shekhar A., Potter W.Z., Lightfoot J., Lienemann J., Dube S., Mallinckrodt C., Bymaster F.P., McKinzie D.L., Felder C.C. (2008). Selective Muscarinic Receptor Agonist Xanomeline as a Novel Treatment Approach for Schizophrenia. Am. J. Psychiatry.

[173] Maehara S., Hikichi H., Ohta H. (2011). Behavioral effects of N-desmethylclozapine on locomotor activity and sensorimotor gating function in mice—Possible involvement of muscarinic receptors. Brain Res..

[174] De Bartolomeis A., Ciccarelli M., Vellucci L., Fornaro M., Iasevoli F., Barone A. (2022). Update on novel antipsychotics and pharmacological strategies for treatment-resistant schizophrenia. Expert Opin. Pharmacother..

[175] Lloyd G.K., Williams M. (2000). Neuronal nicotinic acetylcholine receptors as novel drug targets. Experiment.

[176] Simosky J.K., Stevens K.E., Adler L.E., Freedman R. (2003). Clozapine improves deficient inhibitory auditory processing in DBA/2 mice, via a nicotinic cholinergic mechanism. Psychopharmacology.

[177] Unal G., Sirvanci S., Aricioglu F. (2020). α7 nicotinic receptor agonist and positive allosteric modulators differently improved schizophrenia-like cognitive deficits in male rats. Behav. Brain Res..

[178] George T.P., Sernyak M.J., Ziedonis D.M., Woods S.W. (1995). Effects of clozapine on smoking in chronic schizophrenic outpatients. J. Clin. Psychiatry.

[179] McEvoy J., Freudenreich O., McGee M., Vanderzwaag C., Levin E., Rose J. (1995). Clozapine decreases smoking in patients with chronic schizophrenia. Biol. Psychiatry.

[180] Wu B.-J., Chen H.-K., Lee S.-M. (2013). Do Atypical Antipsychotics Really Enhance Smoking Reduction More Than Typical Ones?. J. Clin. Psychopharmacol..

[181] Simosky K.E.S.A.R.F.J.K., Stevens K., Freedman R. (2002). Nicotinic Agonists and Psychosis. Curr. Drug Target -CNS Neurol. Disord..

[182] Unal G., Bekci H., Cumaoglu A., Yerer M., Aricioglu F. (2020). Alpha 7 nicotinic receptor agonist and positive allosteric modulators improved social and molecular deficits of MK-801 model of schizophrenia in rats. Pharmacol. Biochem. Behav..

[183] Keefe R.S.E., Meltzer H.A., Dgetluck N., Gawryl M., Koenig G., Moebius H.J., Lombardo I., Hilt D.C. (2015). Randomized, Double-Blind, Placebo-Controlled Study of Encenicline, an α7 Nicotinic Acetylcholine Receptor Agonist, as a Treatment for Cognitive Impairment in Schizophrenia. Neuropsychopharmacology.

[184] Rowe A.R., Mercer L., Casetti V., Sendt K.-V., Giaroli G., Shergill S.S., Tracy D.K. (2015). Dementia praecox redux: A systematic review of the nicotinic receptor as a target for cognitive symptoms of schizophrenia. J. Psychopharmacol..

[185] Money T., Scarr E., Udawela M., Gibbons A., Jeon W., Seo M., Dean B. (2010). Treating schizophrenia: Novel targets for the cholinergic system. CNS Neurol. Disord. Drug Targets.

[186] Haas H.L., Panula P. (2003). The role of histamine and the tuberomamillary nucleus in the nervous system. Nat. Rev. Neurosci..

[187] Hill S.J., Ganellin C.R., Timmerman H., Schwartz J.C., Shankley N.P., Young J.M., Schunack W., Levi R., Haas H.L. (1997). International Union of Pharmacology. XIII. Classification of histamine receptors. Pharmacol. Rev..

[188] Schwartz J.-C., Morisset S., Rouleau A., Tardivel-Lacombe J., Gbahou F., Ligneau X., Héron A., Sasse A., Stark H., Schunack W. (2001). Application of genomics to drug design: The example of the histamine H3 receptor. Eur. Neuropsychopharmacol..

[189] Toyota H., Dugovic C., Koehl M., Laposky A.D., Weber C., Ngo K., Wu Y., Lee D.H., Yanai K., Sakurai E. (2002). Behavioral Characterization of Mice Lacking Histamine H_3_ Receptors. Mol. Pharmacol..

[190] Pillot C., Héron A., Schwartz J.-C., Arrang J.-M. (2003). Ciproxifan, a histamine H_3_-receptor antagonist/inverse agonist, modulates the effects of methamphetamine on neuropeptide mRNA expression in rat striatum. Eur. J. Neurosci..

[191] Ito C. (2009). Histamine H3-Receptor Inverse Agonists as Novel Antipsychotics. Central Nerv. Syst. Agents Med. Chem..

[192] Kathmann M., Schlicker E., Göthert M. (1994). Intermediate affinity and potency of clozapine and low affinity of other neuroleptics and of antidepressants at H3 receptors. Psychopharmacology.

[193] Mahmood D., Akhtar M., Jahan K., Goswami D. (2016). Histamine H3 receptor antagonists display antischizophrenic activities in rats treated with MK-801. J. Basic Clin. Physiol. Pharmacol..

[194] Rodrigues A.A., Jansen F., Leurs R., Timmerman H., Prell G. (1995). Interaction of clozapine with the histamine H3 receptor in rat brain. Br. J. Pharmacol..

[195] Goto A., Mouri A., Nagai T., Yoshimi A., Ukigai M., Tsubai T., Hida H., Ozaki N., Noda Y. (2016). Involvement of the histamine H4 receptor in clozapine-induced hematopoietic toxicity: Vulnerability under granulocytic differentiation of HL-60 cells. Toxicol. Appl. Pharmacol..

[196] Deng C., Weston-Green K., Huang X.-F. (2010). The role of histaminergic H1 and H3 receptors in food intake: A mechanism for atypical antipsychotic-induced weight gain?. Prog. Neuro-Psychopharmacol. Biol. Psychiatry.

[197] Humbert-Claude M., Davenas E., Gbahou F., Vincent L., Arrang J.-M. (2011). Involvement of histamine receptors in the atypical antipsychotic profile of clozapine: A reassessment in vitro and in vivo. Psychopharmacology.

[198] Iwabuchi K., Ito C., Tashiro M., Kato M., Kano M., Itoh M., Iwata R., Matsuoka H., Sato M., Yanai K. (2005). Histamine H1 receptors in schizophrenic patients measured by positron emission tomography. Eur. Neuropsychopharmacol..

[199] Nakai T., Kitamura N., Hashimoto T., Kajimoto Y., Nishino N., Mita T., Tanaka C. (1991). Decreased histamine H1 receptors in the frontal cortex of brains from patients with chronic schizophrenia. Biol. Psychiatry.

[200] Sato H., Ito C., Hiraoka K., Tashiro M., Shibuya K., Funaki Y., Yoshikawa T., Iwata R., Matsuoka H., Yanai K. (2015). Histamine H1 receptor occupancy by the new-generation antipsychotics olanzapine and quetiapine: A positron emission tomography study in healthy volunteers. Psychopharmacology.

[201] Cardozo T., Shmelkov E., Felsovalyi K., Swetnam J., Butler T., Malaspina D., Shmelkov S.V. (2017). Chemistry-based molecular signature underlying the atypia of clozapine. Transl. Psychiatry.

[202] Fang F., Sun H., Wang Z., Ren M., Calabrese J.R., Gao K. (2016). Antipsychotic Drug-Induced Somnolence: Incidence, Mechanisms, and Management. CNS Drugs.

[203] Kim S.F., Huang A.S., Snowman A.M., Teuscher C., Snyder S.H. (2007). Antipsychotic drug-induced weight gain mediated by histamine H_1_ receptor-linked activation of hypothalamic AMP-kinase. Proc. Natl. Acad. Sci. USA.

[204] Kroeze W.K., Hufeisen S.J., A Popadak B., Renock S.M., Steinberg S., Ernsberger P., Jayathilake K., Meltzer H.Y., Roth B.L. (2003). H1-Histamine Receptor Affinity Predicts Short-Term Weight Gain for Typical and Atypical Antipsychotic Drugs. Neuropsychopharmacology.

[205] Solismaa A., Kampman O., Lyytikäinen L.-P., Seppälä N., Viikki M., Mononen N., Lehtimäki T., Leinonen E. (2017). Histaminergic gene polymorphisms associated with sedation in clozapine-treated patients. Eur. Neuropsychopharmacol..

[206] Chen W.-Y., Chen L.-Y., Liu H.-C., Wu C.-S., Yang S.-Y., Pan C.-H., Tsai S.-Y., Chen C.-C., Kuo C.-J. (2019). Correction: Antipsychotic medications and stroke in schizophrenia: A case-crossover study. PLoS ONE.

[207] Roegge C.S., Perraut C., Hao X., Levin E.D. (2007). Histamine H1 receptor involvement in prepulse inhibition and memory function: Relevance for the antipsychotic actions of clozapine. Pharmacol. Biochem. Behav..

[208] Meskanen K., Ekelund H., Laitinen J., Neuvonen P., Haukka J., Panula P., Ekelund J. (2013). A Randomized Clinical Trial of Histamine 2 Receptor Antagonism in Treatment-Resistant Schizophrenia. J. Clin. Psychopharmacol..

[209] Rutigliano G., Accorroni A., Zucchi R. (2018). The Case for TAAR1 as a Modulator of Central Nervous System Function. Front. Pharmacol..

[210] Krogmann A., Peters L., Von Hardenberg L., Bödeker K., Nöhles V.B., Correll C.U. (2019). Keeping up with the therapeutic advances in schizophrenia: A review of novel and emerging pharmacological entities. CNS Spectrums.

[211] Harmeier A., Obermueller S., Meyer C.A., Revel F.G., Buchy D., Chaboz S., Dernick G., Wettstein J.G., Iglesias A., Rolink A. (2015). Trace amine-associated receptor 1 activation silences GSK3β signaling of TAAR1 and D2R heteromers. Eur. Neuropsychopharmacol..

[212] Schwartz M.D., Canales J.J., Zucchi R., Espinoza S., Sukhanov I., Gainetdinov R.R. (2018). Trace amine-associated receptor 1: A multimodal therapeutic target for neuropsychiatric diseases. Expert Opin. Ther. Targets.

[213] Revel F.G., Moreau J.-L., Pouzet B., Mory R., Bradaia A., Buchy D., Metzler V., Chaboz S., Zbinden K.G., Galley G. (2012). A new perspective for schizophrenia: TAAR1 agonists reveal antipsychotic- and antidepressant-like activity, improve cognition and control body weight. Mol. Psychiatry.

[214] Koblan K.S., Kent J., Hopkins S.C., Krystal J.H., Cheng H., Goldman R., Loebel A. (2020). A Non–D2-Receptor-Binding Drug for the Treatment of Schizophrenia. N. Engl. J. Med..

[215] Hopkins S.C., Ogirala A., Worden M., Koblan K.S. (2021). Depicting Safety Profile of TAAR1 Agonist Ulotaront Relative to Reactions Anticipated for a Dopamine D2-Based Pharmacological Class in FAERS. Clin. Drug Investig..

[216] Nakazawa K., Sapkota K. (2019). The origin of NMDA receptor hypofunction in schizophrenia. Pharmacol. Ther..

[217] Lin C.-H., Lin C.-H., Chang Y.-C., Huang Y.-J., Chen P.-W., Yang H.-T., Lane H.-Y. (2018). Sodium Benzoate, a D-Amino Acid Oxidase Inhibitor, Added to Clozapine for the Treatment of Schizophrenia: A Randomized, Double-Blind, Placebo-Controlled Trial. Biol. Psychiatry.

[218] Lane H.-Y., Lin C.-H., Green M.F., Hellemann G., Huang C.-C., Chen P.-W., Tun R., Chang Y.-C., Tsai G.E. (2013). Add-on Treatment of Benzoate for Schizophrenia. JAMA Psychiatry.

[219] Molla G. (2017). Competitive Inhibitors Unveil Structure/Function Relationships in Human D-Amino Acid Oxidase. Front. Mol. Biosci..

[220] Siskind D.J., Lee M., Ravindran A., Zhang Q., Ma E., Motamarri B., Kisely S. (2018). Augmentation strategies for clozapine refractory schizophrenia: A systematic review and meta-analysis. Aust. N. Zealand J. Psychiatry.

[221] De Bartolomeis A., Vellucci L., Austin M.C., De Simone G., Barone A. (2022). Rational and Translational Implications of D-Amino Acids for Treatment-Resistant Schizophrenia: From Neurobiology to the Clinics. Biomolecules.

[222] Goh K.K., Wu T.-H., Chen C.-H., Lu M.-L. (2021). Efficacy of *N*-methyl-*D*-aspartate receptor modulator augmentation in schizophrenia: A meta-analysis of randomised, placebo-controlled trials. J. Psychopharmacol..

[223] Schwieler L., Linderholm K.R., Nilsson-Todd L.K., Erhardt S., Engberg G. (2008). Clozapine interacts with the glycine site of the NMDA receptor: Electrophysiological studies of dopamine neurons in the rat ventral tegmental area. Life Sci..

[224] Alberati D., Moreau J.-L., Lengyel J., Hauser N., Mory R., Borroni E., Pinard E., Knoflach F., Schlotterbeck G., Hainzl D. (2012). Glycine reuptake inhibitor RG1678: A pharmacologic characterization of an investigational agent for the treatment of schizophrenia. Neuropharmacology.

[225] Kantrowitz J.T., Nolan K.A., Epstein M.L., Lehrfeld N., Shope C., Petkova E., Javitt D.C. (2017). Neurophysiological Effects of Bitopertin in Schizophrenia. J. Clin. Psychopharmacol..

[226] Fleischhacker W.W., Podhorna J., Gröschl M., Hake S., Zhao Y., Huang S., E Keefe R.S., Desch M., Brenner R., Walling D.P. (2021). Efficacy and safety of the novel glycine transporter inhibitor BI 425809 once daily in patients with schizophrenia: A double-blind, randomised, placebo-controlled phase 2 study. Lancet Psychiatry.

[227] Costa B.M., Kwapisz L.C., Mehrkens B., Bledsoe D.N., Vacca B.N., Johnston T.V., Razzaq R., Manickam D., Klein B.G. (2021). A glutamate concentration-biased allosteric modulator potentiates NMDA-induced ion influx in neurons. Pharmacol. Res. Perspect..

[228] Frizzo M.E.D.S., Dall’Onder L.P., Dalcin K.B., Souza D.O. (2004). Riluzole Enhances Glutamate Uptake in Rat Astrocyte Cultures. Cell. Mol. Neurobiol..

[229] Carbone M., Duty S., Rattray M. (2012). Riluzole elevates GLT-1 activity and levels in striatal astrocytes. Neurochem. Int..

[230] Chowdhury G.M.I., Banasr M., A De Graaf R., Rothman D.L., Behar K., Sanacora G. (2008). Chronic Riluzole Treatment Increases Glucose Metabolism in Rat Prefrontal Cortex and Hippocampus. J. Cereb. Blood Flow Metab..

[231] Lazarevic V., Yang Y., Ivanova D., Fejtova A., Svenningsson P. (2018). Riluzole attenuates the efficacy of glutamatergic transmission by interfering with the size of the readily releasable neurotransmitter pool. Neuropharmacology.

[232] Pillinger T., Rogdaki M., McCutcheon R.A., Hathway P., Egerton A., Howes O.D. (2019). Altered glutamatergic response and functional connectivity in treatment resistant schizophrenia: The effect of riluzole and therapeutic implications. Psychopharmacology.

[233] Farokhnia M., Sabzabadi M., Pourmahmoud H., Khodaie-Ardakani M.-R., Hosseini S.-M., Yekehtaz H., Tabrizi M., Rezaei F., Salehi B., Akhondzadeh S. (2013). A double-blind, placebo controlled, randomized trial of riluzole as an adjunct to risperidone for treatment of negative symptoms in patients with chronic schizophrenia. Psychopharmacology.

[234] Nakazawa K., Jeevakumar V., Nakao K. (2017). Spatial and temporal boundaries of NMDA receptor hypofunction leading to schizophrenia. Schizophrenia.

[235] Corbett R., Camacho F., Woods A.T., Kerman L.L., Fishkin R.J., Brooks K., Dunn R.W. (1995). Antipsychotic agents antagonize non-competitiveN-methyl-d-aspartate antagonist-induced behaviors. Psychopharmacology.

[236] Sheng M., Hoogenraad C.C. (2007). The Postsynaptic Architecture of Excitatory Synapses: A More Quantitative View. Annu. Rev. Biochem..

[237] De Bartolomeis A., Sarappa C., Magara S., Iasevoli F. (2012). Targeting glutamate system for novel antipsychotic approaches: Relevance for residual psychotic symptoms and treatment resistant schizophrenia. Eur. J. Pharmacol..

[238] Gao C., Tronson N.C., Radulovic J. (2013). Modulation of behavior by scaffolding proteins of the post-synaptic density. Neurobiol. Learn. Mem..

[239] Iasevoli F., Tomasetti C., De Bartolomeis A. (2013). Scaffolding Proteins of the Post-synaptic Density Contribute to Synaptic Plasticity by Regulating Receptor Localization and Distribution: Relevance for Neuropsychiatric Diseases. Neurochem. Res..

[240] Wilson R.S., Rauniyar N., Sakaue F., Lam T.T., Williams K.R., Nairn A.C. (2019). Development of Targeted Mass Spectrometry-Based Approaches for Quantitation of Proteins Enriched in the Postsynaptic Density (PSD). Proteomes.

[241] Suzuki T., Kametani K., Guo W., Li W. (2017). Protein components of post-synaptic density lattice, a backbone structure for type I excitatory synapses. J. Neurochem..

[242] De Bartolomeis A., Tomasetti C. (2012). Calcium-Dependent Networks in Dopamine–Glutamate Interaction: The Role of Postsynaptic Scaffolding Proteins. Mol. Neurobiol..

[243] Chen X., Winters C., Azzam R., Li X., Galbraith J.A., Leapman R.D., Reese T.S. (2008). Organization of the core structure of the postsynaptic density. Proc. Natl. Acad. Sci. USA.

[244] Owczarek S., Hou J., Secher T., Kristiansen L. (2011). Phencyclidine treatment increases NR2A and NR2B N-methyl-D-aspartate receptor subunit expression in rats. Neuroreport.

[245] MacDonald M.L., Ding Y., Newman J., Hemby S., Penzes P., Lewis D.A., Yates N.A., Sweet R.A. (2014). Altered Glutamate Protein Co-Expression Network Topology Linked to Spine Loss in the Auditory Cortex of Schizophrenia. Biol. Psychiatry.

[246] Clinton S.M., Haroutunian V., Davis K.L., Meador-Woodruff J.H. (2003). Altered Transcript Expression of NMDA Receptor-Associated Postsynaptic Proteins in the Thalamus of Subjects With Schizophrenia. Am. J. Psychiatry.

[247] Shen Y.-C., Liao D.-L., Chen J.-Y., Wang Y.-C., Lai I.-C., Liou Y.-J., Chen Y.-J., Luu S.-U., Chen C.-H. (2009). Resequencing and association study of vesicular glutamate transporter 1 gene (VGLUT1) with schizophrenia. Schizophr. Res..

[248] Oni-Orisan A., Kristiansen L.V., Haroutunian V., Meador-Woodruff J.H., McCullumsmith R.E. (2008). Altered Vesicular Glutamate Transporter Expression in the Anterior Cingulate Cortex in Schizophrenia. Biol. Psychiatry.

[249] Iwata Y., Nakajima S., Plitman E., Caravaggio F., Kim J., Shah P., Mar W., Chavez S., De Luca V., Mimura M. (2018). Glutamatergic Neurometabolite Levels in Patients With Ultra-Treatment-Resistant Schizophrenia: A Cross-Sectional 3T Proton Magnetic Resonance Spectroscopy Study. Biol. Psychiatry.

[250] Webster M.J., Elashoff M., Weickert C.S. (2011). Molecular evidence that cortical synaptic growth predominates during the first decade of life in humans. Int. J. Dev. Neurosci..

[251] Kaizuka T., Takumi T. (2018). Postsynaptic density proteins and their involvement in neurodevelopmental disorders. J. Biochem..

[252] (2015). The Network and Pathway Analysis Subgroup of the Psychiatric Genomics Consortium Psychiatric genome-wide association study analyses implicate neuronal, immune and histone pathways. Nat. Neurosci..

[253] Soler J., Fañanás L., Parellada M., Krebs M.-O., Rouleau G.A., Fatjó-Vilas M. (2018). Genetic variability in scaffolding proteins and risk for schizophrenia and autism-spectrum disorders: A systematic review. J. Psychiatry Neurosci..

[254] Leber S.L., Llenos I.C., Miller C.L., Dulay J.R., Haybaeck J., Weis S. (2017). Homer1a protein expression in schizophrenia, bipolar disorder, and major depression. J. Neural Transm..

[255] Coley A.A., Gao W.-J. (2017). PSD95: A synaptic protein implicated in schizophrenia or autism?. Prog. Neuro-Psychopharmacol. Biol. Psychiatry.

[256] Fromer M., Pocklington A.J., Kavanagh D.H., Williams H.J., Dwyer S., Gormley P., Georgieva L., Rees E., Palta P., Ruderfer D.M. (2014). De novo mutations in schizophrenia implicate synaptic networks. Nature.

[257] Kirov G., Pocklington A.J., Holmans P., Ivanov D., Ikeda M., Ruderfer D., Moran J., Chambert K., Toncheva D., Georgieva L. (2011). De novo CNV analysis implicates specific abnormalities of postsynaptic signalling complexes in the pathogenesis of schizophrenia. Mol. Psychiatry.

[258] Ting J.T., Peça J., Feng G. (2012). Functional Consequences of Mutations in Postsynaptic Scaffolding Proteins and Relevance to Psychiatric Disorders. Annu. Rev. Neurosci..

[259] Eastwood S., Harrison P. (1995). Decreased synaptophysin in the medial temporal lobe in schizophrenia demonstrated using immunoautoradiography. Neuroscience.

[260] Hotta Y., Ohnuma T., Hanzawa R., Shibata N., Maeshima H., Baba H., Hatano T., Takebayashi Y., Kitazawa M., Higa M. (2011). Association study between Disrupted-in-Schizophrenia-1 (DISC1) and Japanese patients with treatment-resistant schizophrenia (TRS). Prog. Neuro-Psychopharmacol. Biol. Psychiatry.

[261] Blennow K., Bogdanovic N., Gottfries C.-G., Davidsson P. (1999). The Growth-Associated Protein GAP-43 Is Increased in the Hippocampus and in the Gyrus Cinguli in Schizophrenia. J. Mol. Neurosci..

[262] Webster M.J., Weickert C.S., Herman M.M., Hyde T.M., Kleinman J.E. (2001). Synaptophysin and GAP-43 mRNA levels in the hippocampus of subjects with schizophrenia. Schizophr. Res..

[263] Glantz L.A. (1997). Reduction of Synaptophysin Immunoreactivity in the Prefrontal Cortex of Subjects With Schizophrenia. Arch. Gen. Psychiatry.

[264] De Bartolomeis A., Latte G., Tomasetti C., Iasevoli F. (2013). Glutamatergic Postsynaptic Density Protein Dysfunctions in Synaptic Plasticity and Dendritic Spines Morphology: Relevance to Schizophrenia and Other Behavioral Disorders Pathophysiology, and Implications for Novel Therapeutic Approaches. Mol. Neurobiol..

[265] De Bartolomeis A., Avagliano C., Vellucci L., D’Ambrosio L., Manchia M., D’Urso G., Buonaguro E.F., Iasevoli F. (2019). Translating preclinical findings in clinically relevant new antipsychotic targets: Focus on the glutamatergic postsynaptic density. Implications for treatment resistant schizophrenia. Neurosci. Biobehav. Rev..

[266] Li C., Wang A., Wang C., Ramamurthy J., Zhang E., Guadagno E., Trakadis Y. (2018). Metabolomics in patients with psychosis: A systematic review. Am. J. Med. Genet. Part B Neuropsychiatr. Genet..

[267] Föcking M., Lopez L.M., A English J., Dicker P., Wolff A., Brindley E., Wynne K., Cagney G., Cotter D.R. (2014). Proteomic and genomic evidence implicates the postsynaptic density in schizophrenia. Mol. Psychiatry.

[268] Matosin N., Fernandez-Enright F., Lum J.S., Newell K.A. (2017). Shifting towards a model of mGluR5 dysregulation in schizophrenia: Consequences for future schizophrenia treatment. Neuropharmacology.

[269] Bipolar Disorder and Schizophrenia Working Group of the Psychiatric Genomics Consortium (2018). Genomic Dissection of Bipolar Disorder and Schizophrenia, Including 28 Subphenotypes. Cell.

[270] Tomasetti C., Iasevoli F., Buonaguro E.F., De Berardis D., Fornaro M., Fiengo A.L.C., Martinotti G., Orsolini L., Valchera A., Di Giannantonio M. (2017). Treating the Synapse in Major Psychiatric Disorders: The Role of Postsynaptic Density Network in Dopamine-Glutamate Interplay and Psychopharmacologic Drugs Molecular Actions. Int. J. Mol. Sci..

[271] Brocos-Mosquera I., Gabilondo A.M., Meana J.J., Callado L.F., Erdozain A.M. (2020). Spinophilin expression in postmortem prefrontal cortex of schizophrenic subjects: Effects of antipsychotic treatment. Eur. Neuropsychopharmacol..

[272] Funk A.J., Mielnik C.A., Koene R., Newburn E., Ramsey A.J., Lipska B.K., McCullumsmith R.E. (2017). Postsynaptic Density-95 Isoform Abnormalities in Schizophrenia. Schizophr. Bull..

[273] Takaki M., Kodama M., Mizuki Y., Kawai H., Yoshimura B., Kishimoto M., Sakamoto S., Okahisa Y., Yamada N. (2018). Effects of the antipsychotics haloperidol, clozapine, and aripiprazole on the dendritic spine. Eur. Neuropsychopharmacol..

[274] Toyooka K., Iritani S., Makifuchi T., Shirakawa O., Kitamura N., Maeda K., Nakamura R., Niizato K., Watanabe M., Kakita A. (2002). Selective reduction of a PDZ protein, SAP-97, in the prefrontal cortex of patients with chronic schizophrenia. J. Neurochem..

[275] Norton N., Williams H., Williams N., Spurlock G., Zammit S., Jones G., Jones S., Owen R., O’Donovan M., Owen M. (2003). Mutation screening of theHomer gene family and association analysis in schizophrenia. Am. J. Med. Genet..

[276] Szumlinski K.K., Lominac K.D., Kleschen M.J., Oleson E.B., Dehoff M.H., Schwartz M.K., Seeberg P.H., Worley P.F., Kalivas P.W. (2005). Behavioral and neurochemical phenotyping of Homer1 mutant mice: Possible relevance to schizophrenia. Genes Brain Behav..

[277] Kato A., Ozawa F., Saitoh Y., Fukazawa Y., Sugiyama H., Inokuchi K. (1998). Novel Members of the Vesl/Homer Family of PDZ Proteins That Bind Metabotropic Glutamate Receptors. J. Biol. Chem..

[278] Xiao B., Tu J.C., Petralia R.S., Yuan J.P., Doan A., Breder C.D., Ruggiero A., A Lanahan A., Wenthold R.J., Worley P.F. (1998). Homer Regulates the Association of Group 1 Metabotropic Glutamate Receptors with Multivalent Complexes of Homer-Related, Synaptic Proteins. Neuron.

[279] Brakeman P.R., Lanahan A.A., O’Brien R., Roche K., Barnes C.A., Huganir R.L., Worley P.F. (1997). Homer: A protein that selectively binds metabotropic glutamate receptors. Nature.

[280] Ghasemzadeh M.B., Windham L.K., Lake R.W., Acker C.J., Kalivas P.W. (2009). Cocaine activates Homer1 immediate early gene transcription in the mesocorticolimbic circuit: Differential regulation by dopamine and glutamate signaling. Synapse.

[281] Sala C., Futai K., Yamamoto K., Worley P.F., Hayashi Y., Sheng M. (2003). Inhibition of Dendritic Spine Morphogenesis and Synaptic Transmission by Activity-Inducible Protein Homer1a. J. Neurosci..

[282] Borod J.C. (1992). Interhemispheric and intrahemispheric control of emotion: A focus on unilateral brain damage. J. Consult. Clin. Psychol..

[283] Ambesi-Impiombato A., Panariello F., Dell’Aversano C., Tomasetti C., Muscettola G., de Bartolomeis A. (2007). Differential expression ofHomer 1 gene by acute and chronic administration of antipsychotics and dopamine transporter inhibitors in the rat forebrain. Synapse.

[284] Iasevoli F., Ambesi-Impiombato A., Fiore G., Panariello F., Muscettola G., de Bartolomeis A. (2010). Pattern of acute induction of *Homer1a* gene is preserved after chronic treatment with first- and second-generation antipsychotics: Effect of short-term drug discontinuation and comparison with Homer1a-interacting genes. J. Psychopharmacol..

[285] Sotelo J. (1989). PRAZIQUANTEL FOR NEUROCYSTICERCOSIS. Lancet.

[286] Iasevoli F., Tomasetti C., Marmo F., Bravi D., Arnt J., De Bartolomeis A. (2010). Divergent acute and chronic modulation of glutamatergic postsynaptic density genes expression by the antipsychotics haloperidol and sertindole. Psychopharmacology.

[287] Tomasetti C., Dell’Aversano C., Iasevoli F., de Bartolomeis A. (2007). Homer splice variants modulation within cortico-subcortical regions by dopamine D2 antagonists, a partial agonist, and an indirect agonist: Implication for glutamatergic postsynaptic density in antipsychotics action. Neuroscience.

[288] De Bartolomeis A., Iasevoli F., Marmo F., Buonaguro E.F., Eramo A., Rossi R., Avvisati L., Latte G., Tomasetti C. (2015). Progressive recruitment of cortical and striatal regions by inducible postsynaptic density transcripts after increasing doses of antipsychotics with different receptor profiles: Insights for psychosis treatment. Eur. Neuropsychopharmacol..

[289] Tomasetti C., Dell’Aversano C., Iasevoli F., Marmo F., de Bartolomeis A. (2011). The acute and chronic effects of combined antipsychotic–mood stabilizing treatment on the expression of cortical and striatal postsynaptic density genes. Prog. Neuro-Psychopharmacol. Biol. Psychiatry.

[290] Iasevoli F., Fiore G., Cicale M., Muscettola G., de Bartolomeis A. (2010). Haloperidol induces higher Homer1a expression than risperidone, olanzapine and sulpiride in striatal sub-regions. Psychiatry Res..

[291] Barone A., Signoriello S., Latte G., Vellucci L., Giordano G., Avagliano C., Buonaguro E.F., Marmo F., Tomasetti C., Iasevoli F. (2021). Modulation of glutamatergic functional connectivity by a prototypical antipsychotic: Translational inference from a postsynaptic density immediate-early gene-based network analysis. Behav. Brain Res..

[292] De Bartolomeis A., Iasevoli F., Marmo F., Buonaguro E.F., Avvisati L., Latte G., Tomasetti C. (2018). Nicotine and caffeine modulate haloperidol-induced changes in postsynaptic density transcripts expression: Translational insights in psychosis therapy and treatment resistance. Eur. Neuropsychopharmacol..

[293] Dell’Aversano C., Tomasetti C., Iasevoli F., de Bartolomeis A. (2009). Antipsychotic and antidepressant co-treatment: Effects on transcripts of inducible postsynaptic density genes possibly implicated in behavioural disorders. Brain Res. Bull..

[294] De Bartolomeis A., Aloj L., Ambesi-Impiombato A., Bravi D., Caracò C., Muscettola G., Barone P. (2001). Acute administration of antipsychotics modulates Homer striatal gene expression differentially. Mol. Brain Res..

[295] De Bartolomeis A., Marmo F., Buonaguro E.F., Latte G., Tomasetti C., Iasevoli F. (2016). Switching antipsychotics: Imaging the differential effect on the topography of postsynaptic density transcripts in antipsychotic-naïve vs. antipsychotic-exposed rats. Prog. Neuro-Psychopharmacol. Biol. Psychiatry.

[296] Iasevoli F., Tomasetti C., Ambesi-Impiombato A., Muscettola G., de Bartolomeis A. (2009). Dopamine receptor subtypes contribution to Homer1a induction: Insights into antipsychotic molecular action. Prog. Neuro-Psychopharmacol. Biol. Psychiatry.

[297] Polese D., de Serpis A.A., Ambesi-Impiombato A., Muscettola G., de Bartolomeis A. (2002). Homer 1a Gene Expression Modulation by Antipsychotic Drugs Involvement of the Glutamate Metabotropic System and Effects of D-Cycloserine. Neuropsychopharmacology.

[298] De Bartolomeis A., Marmo F., Buonaguro E.F., Rossi R., Tomasetti C., Iasevoli F. (2013). Imaging brain gene expression profiles by antipsychotics: Region-specific action of amisulpride on postsynaptic density transcripts compared to haloperidol. Eur. Neuropsychopharmacol..

[299] Buonaguro E.F., Tomasetti C., Chiodini P., Marmo F., Latte G., Rossi R., Avvisati L., Iasevoli F., de Bartolomeis A. (2016). Postsynaptic density protein transcripts are differentially modulated by minocycline alone or in add-on to haloperidol: Implications for treatment resistant schizophrenia. J. Psychopharmacol..

[300] Buonaguro E.F., Iasevoli F., Marmo F., Eramo A., Latte G., Avagliano C., Tomasetti C., de Bartolomeis A. (2017). Re-arrangements of gene transcripts at glutamatergic synapses after prolonged treatments with antipsychotics: A putative link with synaptic remodeling. Prog. Neuro-Psychopharmacol. Biol. Psychiatry.

[301] Robinet E.A., Geurts M., Maloteaux J.-M., Pauwels P.J. (2001). Chronic treatment with certain antipsychotic drugs preserves upregulation of regulator of G-protein signalling 2 mRNA in rat striatum as opposed to c-fos mRNA. Neurosci. Lett..

[302] Semba J. (1999). Differential effects of acute and chronic treatment with typical and atypical neuroleptics on c-fos mRNA expression in rat forebrain regions using non-radioactive in situ hybridization. Neurochem. Int..

[303] Frånberg O., Marcus M.M., Svensson T.H. (2012). Involvement of 5-HT2A receptor and α2-adrenoceptor blockade in the asenapine-induced elevation of prefrontal cortical monoamine outflow. Synapse.

[304] Kammermeier P.J. (2008). Endogenous Homer Proteins Regulate Metabotropic Glutamate Receptor Signaling in Neurons. J. Neurosci..

[305] Seeman P., Tallerico T. (1998). Antipsychotic drugs which elicit little or no Parkinsonism bind more loosely than dopamine to brain D2 receptors, yet occupy high levels of these receptors. Mol. Psychiatry.

[306] Tappe A., Kuner R. (2006). Regulation of motor performance and striatal function by synaptic scaffolding proteins of the Homer1 family. Proc. Natl. Acad. Sci. USA.

[307] Voorn P., Vanderschuren L.J., Groenewegen H.J., Robbins T.W., Pennartz C.M. (2004). Putting a spin on the dorsal–ventral divide of the striatum. Trends Neurosci..

[308] Szele F.G., Artymyshyn R., Molinoff P.B., Chesselet M.-F. (1991). Heterogeneous distribution of dopamine D2 receptor mRNA in the rat striatum: A quantitative analysis with in situ hybridization histochemistry. Anat. Rec..

[309] Russell V.A., Allin R., Lamm M.C.L., Taljaard J.J.F. (1992). Regional distribution of monoamines and dopamine D1-and D2-receptors in the striatum of the rat. Neurochem. Res..

[310] Fisher R.S., Levine M.S., Sibley D.R., Ariano M.A. (1994). D2 dopamine receptor protein location: Golgi impregnation-gold toned and ultrastructural analysis of the rat neostriatum. J. Neurosci. Res..

[311] Rosa-Neto P., Doudet D.J., Cumming P. (2004). Gradients of dopamine D1- and D2/3-binding sites in the basal ganglia of pig and monkey measured by PET. Neuroimage.

[312] Sykes D.A., Dowling M.R., Charlton S.J. (2010). Measuring Receptor Target Coverage: A Radioligand Competition Binding Protocol for Assessing the Association and Dissociation Rates of Unlabeled Compounds. Curr. Protoc. Pharmacol..

[313] Sykes D.A., Moore H., Stott L., Holliday N., Javitch J.A., Lane J.R., Charlton S.J. (2017). Extrapyramidal side effects of antipsychotics are linked to their association kinetics at dopamine D2 receptors. Nat. Commun..

[314] De Bartolomeis A., Sarappa C., Buonaguro E.F., Marmo F., Eramo A., Tomasetti C., Iasevoli F. (2013). Different effects of the NMDA receptor antagonists ketamine, MK-801, and memantine on postsynaptic density transcripts and their topography: Role of Homer signaling, and implications for novel antipsychotic and pro-cognitive targets in psychosis. Prog. Neuro-Psychopharmacol. Biol. Psychiatry.

[315] Iasevoli F., Polese D., Ambesi-Impiombato A., Muscettola G., de Bartolomeis A. (2007). Ketamine-related expression of glutamatergic postsynaptic density genes: Possible implications in psychosis. Neurosci. Lett..

[316] Fukata Y., Hirano Y., Miyazaki Y., Yokoi N., Fukata M. (2021). Trans-synaptic LGI1–ADAM22–MAGUK in AMPA and NMDA receptor regulation. Neuropharmacology.

[317] Kim E., Niethammer M., Rothschild A., Jan Y.N., Sheng M. (1995). Clustering of Shaker-type K+ channels by interaction with a family of membrane-associated guanylate kinases. Nature.

[318] Fujita-Jimbo E., Tanabe Y., Yu Z., Kojima K., Mori M., Li H., Iwamoto S., Yamagata T., Momoi M.Y., Momoi T. (2015). The association of GPR85 with PSD-95-neuroligin complex and autism spectrum disorder: A molecular analysis. Mol. Autism.

[319] Shiraishi Y., Mizutani A., Mikoshiba K., Furuichi T. (2003). Coincidence in dendritic clustering and synaptic targeting of homer proteins and NMDA receptor complex proteins NR2B and PSD95 during development of cultured hippocampal neurons. Mol. Cell. Neurosci..

[320] Fukata Y., Chen X., Chiken S., Hirano Y., Yamagata A., Inahashi H., Sanbo M., Sano H., Goto T., Hirabayashi M. (2021). LGI1–ADAM22–MAGUK configures transsynaptic nanoalignment for synaptic transmission and epilepsy prevention. Proc. Natl. Acad. Sci. USA.

[321] Haas K.T., Compans B., Letellier M., Bartol T.M., Grillo-Bosch D., Sejnowski T.J., Sainlos M., Choquet D., Thoumine O., Hosy E. (2018). Pre-post synaptic alignment through neuroligin-1 tunes synaptic transmission efficiency. Elife.

[322] Oliva C., Escobedo P., Astorga C., Molina C., Sierralta J. (2011). Role of the maguk protein family in synapse formation and function. Dev. Neurobiol..

[323] Feng W., Zhang M. (2009). Organization and dynamics of PDZ-domain-related supramodules in the postsynaptic density. Nat. Rev. Neurosci..

[324] Won S., Levy J.M., A Nicoll R., Roche K.W. (2017). MAGUKs: Multifaceted synaptic organizers. Curr. Opin. Neurobiol..

[325] Rauch A., Wieczorek D., Graf E., Wieland T., Endele S., Schwarzmayr T., Albrecht B., Bartholdi D., Beygo J., Di Donato N. (2012). Range of genetic mutations associated with severe non-syndromic sporadic intellectual disability: An exome sequencing study. Lancet.

[326] Lelieveld S.H., Reijnders M.R.F., Pfundt R., Yntema H.G., Kamsteeg E.-J., de Vries P., A de Vries B.B., Willemsen M.H., Kleefstra T., Löhner K. (2016). Meta-analysis of 2,104 trios provides support for 10 new genes for intellectual disability. Nat. Neurosci..

[327] Fitzgerald T.W., Gerety S.S., Jones W.D., Van Kogelenberg M., King D.A., McRae J., Morley K.I., Parthiban V., Al-Turki S., Ambridge K. (2015). The Deciphering Developmental Disorders Study. Nature.

[328] Moutton S., Bruel A.-L., Assoum M., Chevarin M., Sarrazin E., Goizet C., Guerrot A.-M., Charollais A., Charles P., Heron D. (2018). Truncating variants of the *DLG4* gene are responsible for intellectual disability with marfanoid features. Clin. Genet..

[329] Rodríguez-Palmero A., Boerrigter M.M., Gómez-Andrés D., Aldinger K.A., Marcos-Alcalde Í., Popp B., Everman D.B., Lovgren A.K., Arpin S., Bahrambeigi V. (2021). DLG4-related synaptopathy: A new rare brain disorder. Anesthesia Analg..

[330] Clinton S.M., Meador-Woodruff J.H. (2004). Abnormalities of the NMDA Receptor and Associated Intracellular Molecules in the Thalamus in Schizophrenia and Bipolar Disorder. Neuropsychopharmacology.

[331] Ohnuma T., Kato H., Arai H., Faull R.L.M., McKenna P.J., Emson P.C. (2000). Gene expression of PSD95 in prefrontal cortex and hippocampus in schizophrenia. Neuroreport.

[332] Funk A.J., Rumbaugh G., Harotunian V., McCullumsmith R.E., Meador-Woodruff J.H. (2009). Decreased expression of NMDA receptor-associated proteins in frontal cortex of elderly patients with schizophrenia. Neuroreport.

[333] Coley A.A., Gao W.-J. (2019). PSD-95 deficiency disrupts PFC-associated function and behavior during neurodevelopment. Sci. Rep..

[334] McEachern E.P., Coley A.A., Yang S.-S., Gao W.-J. (2020). PSD-95 deficiency alters GABAergic inhibition in the prefrontal cortex. Neuropharmacology.

[335] Tsai S.-J., Hong C.-J., Cheng C.-Y., Liao D.-L., Liou Y.-J. (2006). Association study of polymorphisms in post-synaptic density protein 95 (PSD-95) with schizophrenia. J. Neural Transm..

[336] Konradi C., Heckers S. (2001). Antipsychotic drugs and neuroplasticity: Insights into the treatment and neurobiology of schizophrenia. Biol. Psychiatry.

[337] Zhang J., Vinuela A., Neely M.H., Hallett P., Grant S., Miller G.M., Isacson O., Caron M.G., Yao W.-D. (2007). Inhibition of the Dopamine D1 Receptor Signaling by PSD-95. J. Biol. Chem..

[338] Kruse M.S., Prémont J., Krebs M.-O., Jay T.M. (2009). Interaction of dopamine D1 with NMDA NR1 receptors in rat prefrontal cortex. Eur. Neuropsychopharmacol..

[339] Cepeda C., Levine M.S. (1998). Dopamine and N-Methyl-D- Aspartate Receptor Interactions in the Neostriatum. Dev. Neurosci..

[340] Nicola S.M., Surmeier D.J., Malenka R.C. (2000). Dopaminergic Modulation of Neuronal Excitability in the Striatum and Nucleus Accumbens. Annu. Rev. Neurosci..

[341] Cepeda C., Hurst R.S., Altemus K.L., Flores-Hernández J., Calvert C.R., Jokel E.S., Grandy D.K., Low M.J., Rubinstein M., Ariano M.A. (2001). Facilitated Glutamatergic Transmission in the Striatum of D_2_ Dopamine Receptor-Deficient Mice. J. Neurophysiol..

[342] Laruelle M., Frankle W.G., Narendran R., Kegeles L.S., Abi-Dargham A. (2005). Mechanism of action of antipsychotic drugs: From dopamine D2 receptor antagonism to glutamate NMDA facilitation. Clin. Ther..

[343] Sala C., Vicidomini C., Bigi I., Mossa A., Verpelli C. (2015). Shank synaptic scaffold proteins: Keys to understanding the pathogenesis of autism and other synaptic disorders. J. Neurochem..

[344] Hwang J.-I., Kim H.S., Lee J.R., Kim E., Ryu S.H., Suh P.-G. (2005). The Interaction of Phospholipase C-β3 with Shank2 Regulates mGluR-mediated Calcium Signal. J. Biol. Chem..

[345] Grabrucker S., Proepper C., Mangus K., Eckert M., Chhabra R., Schmeisser M.J., Boeckers T.M., Grabrucker A.M. (2014). The PSD protein ProSAP2/Shank3 displays synapto-nuclear shuttling which is deregulated in a schizophrenia-associated mutation. Exp. Neurol..

[346] Onimus O., Valjent E., Fisone G., Gangarossa G. (2022). Haloperidol-Induced Immediate Early Genes in Striatopallidal Neurons Requires the Converging Action of cAMP/PKA/DARPP-32 and mTOR Pathways. Int. J. Mol. Sci..

[347] De Bartolomeis A., Buonaguro E.F., Latte G., Rossi R., Marmo F., Iasevoli F., Tomasetti C. (2017). Immediate-Early Genes Modulation by Antipsychotics: Translational Implications for a Putative Gateway to Drug-Induced Long-Term Brain Changes. Front. Behav. Neurosci..

[348] Slot L.A.B., Lestienne F., Grevoz-Barret C., Newman-Tancredi A., Cussac D. (2009). F15063, a potential antipsychotic with dopamine D2/D3 receptor antagonist and 5-HT1A receptor agonist properties: Influence on immediate-early gene expression in rat prefrontal cortex and striatum. Eur. J. Pharmacol..

[349] De Bartolomeis A., De Simone G., Ciccarelli M., Castiello A., Mazza B., Vellucci L., Barone A. (2022). Antipsychotics-Induced Changes in Synaptic Architecture and Functional Connectivity: Translational Implications for Treatment Response and Resistance. Biomedicines.

[350] Merchant K.M., Dorsa D.M. (1993). Differential induction of neurotensin and c-fos gene expression by typical versus atypical antipsychotics. Proc. Natl. Acad. Sci. USA.

[351] Lyford G.L., Yamagata K., E Kaufmann W., A Barnes C., Sanders L.K., Copeland N.G., Gilbert D.J., A Jenkins N., A Lanahan A., Worley P.F. (1995). Arc, a growth factor and activity-regulated gene, encodes a novel cytoskeleton-associated protein that is enriched in neuronal dendrites. Neuron.

[352] Fujimoto T., Tanaka H., Kumamaru E., Okamura K., Miki N. (2004). Arc interacts with microtubules/microtubule-associated protein 2 and attenuates microtubule-associated protein 2 immunoreactivity in the dendrites. J. Neurosci. Res..

[353] Peebles C.L., Yoo J., Thwin M.T., Palop J.J., Noebels J.L., Finkbeiner S. (2010). Arc regulates spine morphology and maintains network stability in vivo. Proc. Natl. Acad. Sci. USA.

[354] Balu D., Coyle J.T. (2014). Chronic D-serine reverses arc expression and partially rescues dendritic abnormalities in a mouse model of NMDA receptor hypofunction. Neurochem. Int..

[355] Li Y., Pehrson A.L., Waller J.A., Dale E., Sanchez C., Gulinello M. (2015). A critical evaluation of the activity-regulated cytoskeleton-associated protein (Arc/Arg3.1)’s putative role in regulating dendritic plasticity, cognitive processes, and mood in animal models of depression. Front. Neurosci..

[356] Bymaster F.P., Hemrick-Luecke S.K., Perry K.W., Fuller R.W. (1996). Neurochemical evidence for antagonism by olanzapine of dopamine, serotonin, α1-adrenergic and muscarinic receptors in vivo in rats. Psychopharmacology.

[357] Tarazi F.I., Stahl S.M. (2012). Iloperidone, asenapine and lurasidone: A primer on their current status. Expert Opin. Pharmacother..

[358] Xiberas X., Martinot J.L., Mallet L., Artiges E., Loc’H C., Mazière B., Paillère-Martinot M.L. (2001). Extrastriatal and striatal D_2_ dopamine receptor blockade with haloperidol or new antipsychotic drugs in patients with schizophrenia. Br. J. Psychiatry.

[359] Morgan J.I., Curran T. (1991). Stimulus-Transcription Coupling in the Nervous System: Involvement of the Inducible Proto-Oncogenes *fos* and *jun*. Annu. Rev. Neurosci..

[360] Cai G., Lu Y., Chen J., Yang D., Yan R., Ren M., He S., Wu S., Zhao Y. (2022). Brain-wide mapping of c-Fos expression with fluorescence micro-optical sectioning tomography in a chronic sleep deprivation mouse model. Neurobiol. Stress.

[361] Grandbarbe L., Bouissac J., Rand M., de Angelis M.H., Artavanis-Tsakonas S., Mohier E. (2003). Delta-Notch signaling controls the generation of neurons/glia from neural stem cells in a stepwise process. Development.

[362] Wei J., Hemmings G.P. (2000). The NOTCH4 locus is associated with susceptibility to schizophrenia. Nat. Genet..

[363] Rapoport J.L., Addington A.M., Frangou S., Psych M.R.C. (2005). The neurodevelopmental model of schizophrenia: Update 2005. Mol. Psychiatry.

[364] Camargo L.M., Collura V., Rain J.-C., Mizuguchi K., Hermjakob H., Kerrien S., Bonnert T.P., Whiting P.J., Brandon N.J. (2007). Disrupted in Schizophrenia 1 Interactome: Evidence for the close connectivity of risk genes and a potential synaptic basis for schizophrenia. Mol. Psychiatry.

[365] Jaaro-Peled H., Hayashi-Takagi A., Seshadri S., Kamiya A., Brandon N.J., Sawa A. (2009). Neurodevelopmental mechanisms of schizophrenia: Understanding disturbed postnatal brain maturation through neuregulin-1–ErbB4 and DISC1. Trends Neurosci..

[366] Lipina T.V., Niwa M., Jaaro-Peled H., Fletcher P.J., Seeman P., Sawa A., Roder J.C. (2010). Enhanced dopamine function in DISC1-L100P mutant mice: Implications for schizophrenia. Genes, Brain Behav..

[367] Su P., Li S., Chen S., Lipina T.V., Wang M., Lai T.K., Lee F.H., Zhang H., Zhai D., Ferguson S.S. (2014). A Dopamine D2 Receptor-DISC1 Protein Complex may Contribute to Antipsychotic-Like Effects. Neuron.

[368] Lipina T.V., Beregovoy N.A., Tkachenko A.A., Petrova E.S., Starostina M.V., Zhou Q., Li S. (2018). Uncoupling DISC1 × D2R Protein-Protein Interactions Facilitates Latent Inhibition in Disc1-L100P Animal Model of Schizophrenia and Enhances Synaptic Plasticity via D2 Receptors. Front. Synaptic Neurosci..

[369] Zheng P., Su Q.P., Jin D., Yu Y., Huang X.-F. (2020). Prevention of Neurite Spine Loss Induced by Dopamine D2 Receptor Overactivation in Striatal Neurons. Front. Neurosci..

[370] Lu B. (2003). BDNF and Activity-Dependent Synaptic Modulation: Figure 1. Learn. Mem..

[371] Purcell A.L., Carew T.J. (2003). Tyrosine kinases, synaptic plasticity and memory: Insights from vertebrates and invertebrates. Trends Neurosci..

[372] Korte M., Carroll P., Wolf E., Brem G., Thoenen H., Bonhoeffer T. (1995). Hippocampal long-term potentiation is impaired in mice lacking brain-derived neurotrophic factor. Proc. Natl. Acad. Sci. USA.

[373] Patterson S.L., Abel T., Deuel T.A., Martin K.C., Rose J.C., Kandel E.R. (1996). Recombinant BDNF Rescues Deficits in Basal Synaptic Transmission and Hippocampal LTP in BDNF Knockout Mice. Neuron.

[374] Minichiello L., Korte M., Wolfer D., Kühn R., Unsicker K., Cestari V., Rossi-Arnaud C., Lipp H.-P., Bonhoeffer T., Klein R. (1999). Essential Role for TrkB Receptors in Hippocampus-Mediated Learning. Neuron.

[375] Lohof A.M., Ip N.Y., Poo M.-M. (1993). Potentiation of developing neuromuscular synapses by the neurotrophins NT-3 and BDNF. Nature.

[376] Hsiao K., Harony-Nicolas H., Buxbaum J.D., Bozdagi-Gunal O., Benson D.L. (2016). Cyfip1 Regulates Presynaptic Activity during Development. J. Neurosci..

[377] Li Y.-X., Xu Y., Ju D., Lester H.A., Davidson N., Schuman E.M. (1998). Expression of a dominant negative TrkB receptor, T1, reveals a requirement for presynaptic signaling in BDNF-induced synaptic potentiation in cultured hippocampal neurons. Proc. Natl. Acad. Sci. USA.

[378] Schinder A. (2000). The neurotrophin hypothesis for synaptic plasticity. Trends Neurosci..

[379] Jovanovic J.N., Czernik A.J., Fienberg A.A., Greengard P., Sihra T.S. (2000). Synapsins as mediators of BDNF-enhanced neurotransmitter release. Nat. Neurosci..

[380] Pozzo-Miller L.D., Gottschalk W., Zhang L., McDermott K., Du J., Gopalakrishnan R., Oho C., Sheng Z.-H., Lu B. (1999). Impairments in High-Frequency Transmission, Synaptic Vesicle Docking, and Synaptic Protein Distribution in the Hippocampus of BDNF Knockout Mice. J. Neurosci..

[381] Holmes K.C., Trentham D.R., Simmons R., Roberts R., Lister I., Schmitz S., Walker M., Veigel C., Trinick J., Buss F. (2004). Myosin VI: Cellular functions and motor properties. Philos. Trans. R. Soc. B: Biol. Sci..

[382] Krendel M., Mooseker M.S. (2005). Myosins: Tails (and Heads) of Functional Diversity. Physiology.

[383] Yano H., Ninan I., Zhang H., A Milner T., Arancio O., Chao M.V. (2006). BDNF-mediated neurotransmission relies upon a myosin VI motor complex. Nat. Neurosci..

[384] Tyler W.J., Perrett S.P., Pozzo-Miller L.D. (2002). The Role of Neurotrophins in Neurotransmitter Release. Neurosci..

[385] Jiang H., Ashraf G.M., Liu M., Zhao K., Wang Y., Wang L., Xing J., Alghamdi B.S., Li Z., Liu R. (2021). Tilianin Ameliorates Cognitive Dysfunction and Neuronal Damage in Rats with Vascular Dementia via p-CaMKII/ERK/CREB and ox-CaMKII-Dependent MAPK/NF-κB Pathways. Oxidative Med. Cell. Longev..

[386] Fiorentini C., Mattanza C., Collo G., Savoia P., Spano P., Missale C. (2011). The tyrosine phosphatase Shp-2 interacts with the dopamine D1 receptor and triggers D1-mediated Erk signaling in striatal neurons. J. Neurochem..

[387] Zheng P., Hu M., Xie Y., Yu Y., Jaaro-Peled H., Huang X.-F. (2018). Aripiprazole and haloperidol protect neurite lesions via reducing excessive D2R-DISC1 complex formation. Prog. Neuro-Psychopharmacol. Biol. Psychiatry.

[388] Ray M.T., Weickert C.S., Webster M.J. (2014). Decreased BDNF and TrkB mRNA expression in multiple cortical areas of patients with schizophrenia and mood disorders. Transl. Psychiatry.

[389] Favalli G., Li J., Belmonte-De-Abreu P., Wong A.H., Daskalakis Z.J. (2012). The role of BDNF in the pathophysiology and treatment of schizophrenia. J. Psychiatr. Res..

[390] Beaulieu J.-M., Sotnikova T.D., Yao W.-D., Kockeritz L., Woodgett J.R., Gainetdinov R.R., Caron M.G. (2004). Lithium antagonizes dopamine-dependent behaviors mediated by an AKT/glycogen synthase kinase 3 signaling cascade. Proc. Natl. Acad. Sci. USA.

[391] Chlan-Fourney J., Ashe P., Nylen K., Juorio A.V., Li X.-M. (2002). Differential regulation of hippocampal BDNF mRNA by typical and atypical antipsychotic administration. Brain Res..

[392] Grillo R.W., Ottoni G.L., Leke R., Souza D., Portela L.V., Lara D.R. (2007). Reduced serum BDNF levels in schizophrenic patients on clozapine or typical antipsychotics. J. Psychiatr. Res..

[393] Halim N.D., Weickert C.S., McClintock B.W., Weinberger D.R., Lipska B.K. (2004). Effects of Chronic Haloperidol and Clozapine Treatment on Neurogenesis in the Adult Rat Hippocampus. Neuropsychopharmacology.

[394] Jeon J.H., Oh T.R., Park S., Huh S., Kim J.H., Mai B.K., Lee J.H., Kim S.H., Lee M.J. (2021). The Antipsychotic Drug Clozapine Suppresses the RGS4 Polyubiquitylation and Proteasomal Degradation Mediated by the Arg/N-Degron Pathway. Neurotherapeutics.

[395] Chong V.Z., Costain W., Marriott J., Sindwani S., Knauer D.J., Wang J.-F., Young L.T., MacCrimmon D., Mishra R.K. (2004). Differential display polymerase chain reaction reveals increased expression of striatal rat glia-derived nexin following chronic clozapine treatment. Pharmacogenomics J..

[396] Kim S.H., Park S., Yu H.S., Ko K.H., Park H.G., Kim Y.S. (2018). The antipsychotic agent clozapine induces autophagy via the AMPK-ULK1-Beclin1 signaling pathway in the rat frontal cortex. Prog. Neuro-Psychopharmacol. Biol. Psychiatry.

[397] Abekawa T., Ito K., Nakagawa S., Nakato Y., Koyama T. (2011). Effects of aripiprazole and haloperidol on progression to schizophrenia-like behavioural abnormalities and apoptosis in rodents. Schizophr. Res..

[398] Bai O., Zhang H., Li X.-M. (2004). Antipsychotic drugs clozapine and olanzapine upregulate bcl-2 mRNA and protein in rat frontal cortex and hippocampus. Brain Res..

[399] Lundberg M., Curbo S., Bohman H., Agartz I., Ögren S.-O., Patrone C., Mansouri S. (2020). Clozapine protects adult neural stem cells from ketamine-induced cell death in correlation with decreased apoptosis and autophagy. Biosci. Rep..

[400] Qing H., Xu H., Wei Z., Gibson K., Li X.-M. (2003). The ability of atypical antipsychotic drugs vs. haloperidol to protect PC12 cells against MPP^+^-induced apoptosis. Eur. J. Neurosci..

[401] Bai O., Chlan-Fourney J., Bowen R., Keegan D., Li X.-M. (2002). Expression of brain-derived neurotrophic factor mRNA in rat hippocampus after treatment with antipsychotic drugs. J. Neurosci. Res..

[402] Ghosh A., Carnahan J., Greenberg M.E., Bodmer H., Viville S., Benoist C., Mathis D. (1994). Requirement for BDNF in Activity-Dependent Survival of Cortical Neurons. Science.

[403] Parikh V., Khan M.M., Terry A., Mahadik S.P. (2004). Differential effects of typical and atypical antipsychotics on nerve growth factor and choline acetyltransferase expression in the cortex and nucleus basalis of rats. J. Psychiatr. Res..

[404] Shao Z., Dyck L.E., Wang H., Li X.-M. (2006). Antipsychotic drugs cause glial cell line-derived neurotrophic factor secretion from C6 glioma cells. J. Psychiatry Neurosci..

[405] Turner B., Rembach A., Spark R., Lopes E., Cheema S. (2003). Opposing effects of low and high-dose clozapine on survival of transgenic amyotrophic lateral sclerosis mice. J. Neurosci. Res..

[406] Einoch R., Weinreb O., Mandiuk N., Youdim M.B., Bilker W., Silver H. (2017). The involvement of BDNF-CREB signaling pathways in the pharmacological mechanism of combined SSRI- antipsychotic treatment in schizophrenia. Eur. Neuropsychopharmacol..

[407] Yang C.R., Zhang X.Y., Liu Y., Du J.Y., Liang R., Yu M., Zhang F.Q., Mu X.F., Li F., Zhou L. (2019). Antidepressant Drugs Correct the Imbalance Between proBDNF/p75NTR/Sortilin and Mature BDNF/TrkB in the Brain of Mice with Chronic Stress. Neurotox. Res..

[408] Jeon S., Kim Y., Chung I.-W., Kim Y.S. (2015). Clozapine induces chloride channel-4 expression through PKA activation and modulates CDK5 expression in SH-SY5Y and U87 cells. Prog. Neuro-Psychopharmacol. Biol. Psychiatry.

[409] Zeng Z., Wang X., Bhardwaj S.K., Zhou X., Little P.J., Quirion R., Srivastava L.K., Zheng W. (2016). The Atypical Antipsychotic Agent, Clozapine, Protects Against Corticosterone-Induced Death of PC12 Cells by Regulating the Akt/FoxO3a Signaling Pathway. Mol. Neurobiol..

[410] Samuels I.S., Saitta S.C., Landreth G.E. (2009). MAP’ing CNS Development and Cognition: An ERKsome Process. Neuron.

[411] Olianas M.C., Dedoni S., Ambu R., Onali P. (2009). Agonist activity of N-desmethylclozapine at δ-opioid receptors of human frontal cortex. Eur. J. Pharmacol..

[412] Alimohamad H., Rajakumar N., Seah Y.-H., Rushlow W. (2005). Antipsychotics alter the protein expression levels of β-catenin and GSK-3 in the rat medial prefrontal cortex and striatum. Biol. Psychiatry.

[413] Kozlovsky N., Amar S., Belmaker R.H., Agam G. (2005). Psychotropic drugs affect Ser9-phosphorylated GSK-3β protein levels in rodent frontal cortex. Int. J. Neuropsychopharmacol..

[414] Alimohamad H., Sutton L., Mouyal J., Rajakumar N., Rushlow W.J. (2005). The effects of antipsychotics on β-catenin, glycogen synthase kinase-3 and dishevelled in the ventral midbrain of rats. J. Neurochem..

[415] Xi D., Li Y.-C., Snyder M.A., Gao R.Y., Adelman A.E., Zhang W., Shumsky J.S., Gao W.-J. (2011). Group II Metabotropic Glutamate Receptor Agonist Ameliorates MK801-Induced Dysfunction of NMDA Receptors via the Akt/GSK-3β Pathway in Adult Rat Prefrontal Cortex. Neuropsychopharmacology.

[416] Ahmed M.R., Gurevich V.V., Dalby K.N., Benovic J.L., Gurevich E.V. (2008). Haloperidol and Clozapine Differentially Affect the Expression of Arrestins, Receptor Kinases, and Extracellular Signal-Regulated Kinase Activation. Experiment.

[417] Kenakin T.P. (2012). Biased signalling and allosteric machines: New vistas and challenges for drug discovery. Br. J. Pharmacol..

[418] Kobayashi Y., Iwakura Y., Sotoyama H., Kitayama E., Takei N., Someya T., Nawa H. (2019). Clozapine-dependent inhibition of EGF/neuregulin receptor (ErbB) kinases. Transl. Psychiatry.

[419] Titulaer J., Radhe O., Danielsson K., Dutheil S., Marcus M., Jardemark K., Svensson T., Snyder G., Ericson M., Davis R. (2022). Lumateperone-mediated effects on prefrontal glutamatergic receptor-mediated neurotransmission: A dopamine D1 receptor dependent mechanism. Eur. Neuropsychopharmacol..

[420] Kumar B., Kuhad A. (2018). Lumateperone: A new treatment approach for neuropsychiatric disorders. Drugs Today.

[421] Dutheil S., Watson L.S., Davis R.E., Snyder G.L. (2022). Lumateperone Normalizes Pathological Levels of Acute Inflammation through Important Pathways Known to Be Involved in Mood Regulation. J. Neurosci..

[422] Carvelli L., Morón J.A., Kahlig K.M., Ferrer J.V., Sen N., Lechleiter J.D., Leeb-Lundberg L.M.F., Merrill G., Lafer E., Ballou L.M. (2002). PI 3-kinase regulation of dopamine uptake. J. Neurochem..

[423] Chen P., Gu Z., Liu W., Yan Z. (2007). Glycogen Synthase Kinase 3 Regulates *N*-Methyl-d-aspartate Receptor Channel Trafficking and Function in Cortical Neurons. Mol. Pharmacol..

[424] Karbownik M.S., Szemraj J., Wieteska Ł., Antczak A., Górski P., Kowalczyk E., Pietras T. (2016). Antipsychotic Drugs Differentially Affect mRNA Expression of Genes Encoding the Neuregulin 1-Downstream ErbB4-PI3K Pathway. Pharmacology.

[425] Liu L., Luo Y., Zhang G., Jin C., Zhou Z., Cheng Z., Yuan G. (2016). The mRNA expression of DRD2, PI3KCB, and AKT1 in the blood of acute schizophrenia patients. Psychiatry Res..

[426] Dadalko O.I., A Siuta M., Poe A.M., Erreger K., Matthies H., Niswender K.D., Galli A. (2015). mTORC2/Rictor Signaling Disrupts Dopamine-Dependent Behaviors via Defects in Striatal Dopamine Neurotransmission. J. Neurosci..

[427] Schwarcz R., Whetsell W.O., Mangano R.M. (1983). Quinolinic Acid: An Endogenous Metabolite That Produces Axon-Sparing Lesions in Rat Brain. Science.

[428] Perkins M., Stone T. (1982). An iontophoretic investigation of the actions of convulsant kynurenines and their interaction with the endogenous excitant quinolinic acid. Brain Res..

[429] Nilsson L.K., Linderholm K.R., Erhardt S. (2005). Subchronic treatment with kynurenine and probenecid: Effects on prepulse inhibition and firing of midbrain dopamine neurons. J. Neural Transm..

[430] Erhardt S., Schwieler L., Emanuelsson C., Geyer M. (2004). Endogenous kynurenic acid disrupts prepulse inhibition. Biol. Psychiatry.

[431] Chess A.C., Simoni M.K., Alling T.E., Bucci D.J. (2007). Elevations of Endogenous Kynurenic Acid Produce Spatial Working Memory Deficits. Schizophr. Bull..

[432] Takahashi N., Sakurai T., Davis K.L., Buxbaum J.D. (2011). Linking oligodendrocyte and myelin dysfunction to neurocircuitry abnormalities in schizophrenia. Prog. Neurobiol..

[433] Kroken R.A., Løberg E.-M., Drønen T., Grüner R., Hugdahl K., Kompus K., Skrede S., Johnsen E. (2014). A Critical Review of Pro-Cognitive Drug Targets in Psychosis: Convergence on Myelination and Inflammation. Front. Psychiatry.

[434] Ozawa K., Hashimoto K., Kishimoto T., Shimizu E., Ishikura H., Iyo M. (2006). Immune Activation During Pregnancy in Mice Leads to Dopaminergic Hyperfunction and Cognitive Impairment in the Offspring: A Neurodevelopmental Animal Model of Schizophrenia. Biol. Psychiatry.

[435] De Bartolomeis A., Barone A., Vellucci L., Mazza B., Austin M.C., Iasevoli F., Ciccarelli M. (2022). Linking Inflammation, Aberrant Glutamate-Dopamine Interaction, and Post-synaptic Changes: Translational Relevance for Schizophrenia and Antipsychotic Treatment: A Systematic Review. Mol. Neurobiol..

[436] Becher B., Spath S., Goverman J. (2016). Cytokine networks in neuroinflammation. Nat. Rev. Immunol..

[437] Wang M., Ling K.-H., Tan J., Lu C.-B. (2020). Development and Differentiation of Midbrain Dopaminergic Neuron: From Bench to Bedside. Cells.

[438] Ling Z.D., Potter E.D., Lipton J.W., Carvey P.M. (1998). Differentiation of Mesencephalic Progenitor Cells into Dopaminergic Neurons by Cytokines. Exp. Neurol..

[439] Jarskog L., Xiao H., Wilkie M.B., Lauder J.M., Gilmore J.H. (1997). Cytokine regulation of embryonic rat dopamine and serotonin neuronal survival in vitro. Int. J. Dev. Neurosci..

[440] Aguilar-Valles A., Jung S., Poole S., Flores C., Luheshi G.N. (2012). Leptin and interleukin-6 alter the function of mesolimbic dopamine neurons in a rodent model of prenatal inflammation. Psychoneuroendocrinology.

[441] Sriram K., Matheson J.M., Benkovic S.A., Miller D.B., Luster M.I., O’Callaghan J.P. (2002). Mice deficient in TNF receptors are protected against dopaminergic neurotoxicity: Implications for Parkinson’s disease. FASEB J..

[442] Leboyer M., Godin O., Terro E., Boukouaci W., Lu C.-L., Andre M., Aouizerate B., Berna F., Barau C., Capdevielle D. (2021). Immune Signatures of Treatment-Resistant Schizophrenia: A FondaMental Academic Centers of Expertise for Schizophrenia (FACE-SZ) Study. Schizophr. Bull. Open.

[443] Dawidowski B., Górniak A., Podwalski P., Lebiecka Z., Misiak B., Samochowiec J. (2021). The Role of Cytokines in the Pathogenesis of Schizophrenia. J. Clin. Med..

[444] Kaplanski G. (2017). Interleukin-18: Biological properties and role in disease pathogenesis. Immunol. Rev..

[445] Syed A.A.S., He L., Shi Y., Mahmood S. (2020). Elevated levels of IL -18 associated with schizophrenia and first episode psychosis: A systematic review and meta-analysis. Early Interv. Psychiatry.

[446] Enache D., Nikkheslat N., Fathalla D., Morgan B.P., Lewis S., Drake R., Deakin B., Walters J., Lawrie S.M., Egerton A. (2021). Peripheral immune markers and antipsychotic non-response in psychosis. Schizophr. Res..

[447] Mantovani A., Dinarello C.A., Molgora M., Garlanda C. (2019). Interleukin-1 and Related Cytokines in the Regulation of Inflammation and Immunity. Immunity.

[448] Dunleavy C., Elsworthy R.J., Upthegrove R., Wood S.J., Aldred S. (2022). Inflammation in first-episode psychosis: The contribution of inflammatory biomarkers to the emergence of negative symptoms, a systematic review and meta-analysis. Acta Psychiatr. Scand..

[449] Ta T.-T., Dikmen H.O., Schilling S., Chausse B., Lewen A., Hollnagel J.-O., Kann O. (2019). Priming of microglia with IFN-γ slows neuronal gamma oscillations in situ. Proc. Natl. Acad. Sci. USA.

[450] Fang X., Zhang Y., Fan W., Tang W., Zhang C. (2017). Interleukin-17 Alteration in First-Episode Psychosis: A Meta-Analysis. Complex Psychiatry.

[451] Liu J.-Y., Chen H.-Y., Lin J.-J., Lu M.-K., Tan H.-P., Jang F.-L., Lin S.-H. (2020). Alterations of plasma cytokine biomarkers for identifying age at onset of schizophrenia with neurological soft signs. Int. J. Med Sci..

[452] Lin A., Kenis G., Bignotti S., Tura G.-J., De Jong R., Bosmans E., Pioli R., Altamura C., Scharpé S., Maes M. (1998). The inflammatory response system in treatment-resistant schizophrenia: Increased serum interleukin-6. Schizophr. Res..

[453] Luo Y., He H., Zhang J., Ou Y., Fan N. (2019). Changes in serum TNF-α, IL-18, and IL-6 concentrations in patients with chronic schizophrenia at admission and at discharge. Compr. Psychiatry.

[454] Mongan D., Ramesar M., Föcking M., Cannon M., Cotter D. (2019). Role of inflammation in the pathogenesis of schizophrenia: A review of the evidence, proposed mechanisms and implications for treatment. Early Interv. Psychiatry.

[455] Schwieler L., Larsson M.K., Skogh E., Kegel M.E., Orhan F., Abdelmoaty S., Finn A., Bhat M., Samuelsson M., Lundberg K. (2015). Increased levels of IL-6 in the cerebrospinal fluid of patients with chronic schizophrenia—Significance for activation of the kynurenine pathway. J. Psychiatry Neurosci..

[456] Marcinowicz P., Więdłocha M., Zborowska N., Dębowska W., Podwalski P., Misiak B., Tyburski E., Szulc A. (2021). A Meta-Analysis of the Influence of Antipsychotics on Cytokines Levels in First Episode Psychosis. J. Clin. Med..

[457] Reale M., Costantini E., Greig N.H. (2021). Cytokine Imbalance in Schizophrenia. From Research to Clinic: Potential Implications for Treatment. Front. Psychiatry.

[458] Wilbers R.H.P., van Raaij D.R., Westerhof L.B., Bakker J., Smant G., Schots A. (2017). Re-evaluation of IL-10 signaling reveals novel insights on the contribution of the intracellular domain of the IL-10R2 chain. PLoS ONE.

[459] Saxton R.A., Tsutsumi N., Su L.L., Abhiraman G.C., Mohan K., Henneberg L.T., Aduri N.G., Gati C., Garcia K.C. (2021). Structure-based decoupling of the pro- and anti-inflammatory functions of interleukin-10. Science.

[460] Shnayder N.A., Khasanova A.K., Strelnik A.I., Al-Zamil M., Otmakhov A.P., Neznanov N.G., Shipulin G.A., Petrova M.M., Garganeeva N.P., Nasyrova R.F. (2022). Cytokine Imbalance as a Biomarker of Treatment-Resistant Schizophrenia. Int. J. Mol. Sci..

[461] Nasi G., Ahmed T., Rasini E., Fenoglio D., Marino F., Filaci G., Cosentino M. (2019). Dopamine inhibits human CD8+ Treg function through D1-like dopaminergic receptors. J. Neuroimmunol..

[462] Pape K., Tamouza R., Leboyer M., Zipp F. (2019). Immunoneuropsychiatry—Novel perspectives on brain disorders. Nat. Rev. Neurol..

[463] Levite M. (2015). Dopamine and T cells: Dopamine receptors and potent effects on T cells, dopamine production in T cells, and abnormalities in the dopaminergic system in T cells in autoimmune, neurological and psychiatric diseases. Acta Physiol..

[464] Vidal P.M., Pacheco R. (2019). Targeting the Dopaminergic System in Autoimmunity. J. Neuroimmune Pharmacol..

[465] Felger J.C., Cole S.W., Pace T.W.W., Hu F., Woolwine B.J., Doho G.H., Raison C.L., Miller A.H. (2011). Molecular signatures of peripheral blood mononuclear cells during chronic interferon-α treatment: Relationship with depression and fatigue. Psychol. Med..

[466] Li W., Knowlton D., Woodward W.R., Habecker B.A. (2003). Regulation of noradrenergic function by inflammatory cytokines and depolarization. J. Neurochem..

[467] Shi W., Meininger C.J., Haynes T.E., Hatakeyama K., Wu G. (2004). Regulation of Tetrahydrobiopterin Synthesis and Bioavailability in Endothelial Cells. Cell Biochem. Biophys..

[468] Amato D., Beasley C., Hahn M.K., Vernon A.C. (2017). Neuroadaptations to antipsychotic drugs: Insights from pre-clinical and human post-mortem studies. Neurosci. Biobehav. Rev..

[469] Maes M., Chiavetto L.B., Bignotti S., Tura G.-J.B., Pioli R., Boin F., Kenis G., Bosmans E., de Jongh R., Lin A. (2000). Effects of atypical antipsychotics on the inflammatory response system in schizophrenic patients resistant to treatment with typical neuroleptics. Eur. Neuropsychopharmacol..

[470] Fernandez-Egea E., Vértes P.E., Flint S.M., Turner L., Mustafa S., Hatton A., Smith K.G.C., Lyons P.A., Bullmore E.T. (2016). Peripheral Immune Cell Populations Associated with Cognitive Deficits and Negative Symptoms of Treatment-Resistant Schizophrenia. PLoS ONE.

[471] Maes M., Bosmans E., Calabrese J., Smith R., Meltzer H.Y. (1995). Interleukin-2 and interleukin-6 in schizophrenia and mania: Effects of neuroleptics and mood stabilizers. J. Psychiatr. Res..

[472] Maes M., Bosmans E., Kenis G., De Jong R., Smith R.S., Meltzer H.Y. (1997). In vivo immunomodulatory effects of clozapine in schizophrenia. Schizophr. Res..

[473] Pollmacher T., Hinze-Selch D., Mullington J. (1996). Effects of Clozapine on Plasma Cytokine and Soluble Cytokine Receptor Levels. J. Clin. Psychopharmacol..

[474] Green L.K., Zareie P., Templeton N., A Keyzers R., Connor B., La Flamme A.C. (2017). Enhanced disease reduction using clozapine, an atypical antipsychotic agent, and glatiramer acetate combination therapy in experimental autoimmune encephalomyelitis. Mult. Scler. J. Exp. Transl. Clin..

[475] Jiang L., Wu X., Wang S., Chen S.-H., Zhou H., Wilson B., Jin C.-Y., Lu R.-B., Xie K., Wang Q. (2016). Clozapine metabolites protect dopaminergic neurons through inhibition of microglial NADPH oxidase. J. Neuroinflammation.

[476] Leykin I., Mayer R., Shinitzky M. (1997). Short and long term immunosuppressive effects of clozapine and haloperidol. Immunopharmacology.

[477] Kim H.-W., Cheon Y., Modi H.R., Rapoport S.I., Rao J.S. (2012). Effects of chronic clozapine administration on markers of arachidonic acid cascade and synaptic integrity in rat brain. Psychopharmacology.

[478] Seol I.-W., Kuo N.Y., Kim K.M. (2004). Effects of dopaminergic drugs on the mast cell degranulation and nitric oxide generation in RAW 264.7 cells. Arch. Pharmacal Res..

[479] Szuster-Ciesielska A., Słotwińska M., Stachura A., Stachura A., Marmurowska-Michalowska H H., Kandefer-Szerszen M. (2004). Neuroleptics modulate cytokine and reactive oxygen species production in blood leukocytes of healthy volunteers. Arch. Immunol. Ther. Exp..

[480] Maes M. (2002). Increased serum interleukin-8 and interleukin-10 in schizophrenic patients resistant to treatment with neuroleptics and the stimulatory effects of clozapine on serum leukemia inhibitory factor receptor. Schizophr. Res..

[481] Song C., Lin A.-H., Kenis G., Bosmans E., Maes M. (2000). Immunosuppressive effects of clozapine and haloperidol: Enhanced production of the interleukin-1 receptor antagonist. Schizophr. Res..

[482] Sugino H., Futamura T., Mitsumoto Y., Maeda K., Marunaka Y. (2008). Atypical antipsychotics suppress production of proinflammatory cytokines and up-regulate interleukin-10 in lipopolysaccharide-treated mice. Prog. Neuro-Psychopharmacol. Biol. Psychiatry.

[483] Himmerich H., Schönherr J., Fulda S., Sheldrick A.J., Bauer K., Sack U. (2011). Impact of antipsychotics on cytokine production in-vitro. J. Psychiatr. Res..

[484] Chen M.-L., Tsai T.-C., Wang L.-K., Lin Y.-Y., Tsai Y.-M., Lee M.-C., Tsai F.-M. (2012). Clozapine inhibits Th1 cell differentiation and causes the suppression of IFN-γ production in peripheral blood mononuclear cells. Immunopharmacol. Immunotoxicol..

[485] Yuhas Y., Ashkenazi S., Berent E., Weizman A. (2022). Clozapine Suppresses the Gene Expression and the Production of Cytokines and Up-Regulates Cyclooxygenase 2 mRNA in Human Astroglial Cells. Brain Sci..

[486] Hahn C.-G., Wang H.-Y., Cho D.-S., Talbot K., Gur R.E., Berrettini W.H., Bakshi K., Kamins J., Borgmann-Winter K.E., Siegel S.J. (2006). Altered neuregulin 1–erbB4 signaling contributes to NMDA> receptor hypofunction in schizophrenia. Nat. Med..

[487] Abu-Ata S., Shukha O.N., Awad-Igbaria Y., Ginat K., Palzur E., Golani I., Shamir A. (2023). Blocking the ErbB pathway during adolescence affects the induction of anxiety-like behavior in young adult maternal immune activation offspring. Pharmacol. Biochem. Behav..

[488] Jijón-Lorenzo R., Caballero-Florán I.H., Recillas-Morales S., Cortés H., Avalos-Fuentes J.A., Paz-Bermúdez F.J., Erlij D., Florán B. (2018). Presynaptic Dopamine D2 Receptors Modulate [ 3 H]GABA Release at StriatoPallidal Terminals via Activation of PLC → IP3 → Calcineurin and Inhibition of AC → cAMP → PKA Signaling Cascades. Neuroscience.

[489] Kim S.H., Ryan T.A. (2013). Balance of Calcineurin Aα and CDK5 Activities Sets Release Probability at Nerve Terminals. J. Neurosci..

[490] Strack S., Kini S., Ebner F.F., Wadzinski B.E., Colbran R.J. (1999). Differential cellular and subcellular localization of protein phosphatase 1 isoforms in brain. J. Comp. Neurol..

[491] Ouimet C., Miller P., Hemmings H., Walaas S., Greengard P. (1984). DARPP-32, a dopamine- and adenosine 3’:5’-monophosphate-regulated phosphoprotein enriched in dopamine-innervated brain regions. III. Immunocytochemical localization. J. Neurosci..

[492] Ouimet C.C., Langley-Gullion K.C., Greengard P. (1998). Quantitative immunocytochemistry of DARPP-32-expressing neurons in the rat caudatoputamen. Brain Res..

[493] Wang H., Farhan M., Xu J., Lazarovici P., Zheng W. (2017). The involvement of DARPP-32 in the pathophysiology of schizophrenia. Oncotarget.

[494] Bonito-Oliva A., Dupont C., Madjid N., Ögren S.O., Fisone G. (2015). Involvement of the Striatal Medium Spiny Neurons of the Direct Pathway in the Motor Stimulant Effects of Phencyclidine. Int. J. Neuropsychopharmacol..

[495] Ishikawa M., Mizukami K., Iwakiri M., Asada T. (2007). Immunohistochemical and immunoblot analysis of Dopamine and cyclic AMP-regulated phosphoprotein, relative molecular mass 32,000 (DARPP-32) in the prefrontal cortex of subjects with schizophrenia and bipolar disorder. Prog. Neuro-Psychopharmacol. Biol. Psychiatry.

[496] Kunii Y., Yabe H., Wada A., Yang Q., Nishiura K., Niwa S.-I. (2011). Altered DARPP-32 expression in the superior temporal gyrus in schizophrenia. Prog. Neuro-Psychopharmacol. Biol. Psychiatry.

[497] Pozzi L., Håkansson K., Usiello A., Borgkvist A., Lindskog M., Greengard P., Fisone G. (2004). Opposite regulation by typical and atypical anti-psychotics of ERK1/2, CREB and Elk-1 phosphorylation in mouse dorsal striatum. J. Neurochem..

[498] Valjent E., Bertran-Gonzalez J., Bowling H., Lopez S., Santini E., Matamales M., Bonito-Oliva A., Hervé D., Hoeffer C., Klann E. (2011). Haloperidol Regulates the State of Phosphorylation of Ribosomal Protein S6 via Activation of PKA and Phosphorylation of DARPP-32. Neuropsychopharmacology.

[499] Bonito-Oliva A., Feyder M., Fisone G. (2011). Deciphering the Actions of Antiparkinsonian and Antipsychotic Drugs on cAMP/DARPP-32 Signaling. Front. Neuroanat..

[500] Takeuchi Y., Fukunaga K. (2004). Different activation of NF-κB by stimulation of dopamine D2L and D2S receptors through calcineurin activation. J. Neurochem..

[501] Adlersberg M., Hsiung S.-C., Glickstein S.B., Liu K.-P., Tamir H., Schmauss C. (2004). Regulation of dopamine D1-receptor activation in vivo by protein phosphatase 2B (calcineurin). J. Neurochem..

[502] Rushlow W.J., Seah Y.H., Belliveau D.J., Rajakumar N. (2005). Changes in calcineurin expression induced in the rat brain by the administration of antipsychotics. J. Neurochem..

[503] Britsch S. (2007). The neuregulin-I/ErbB signaling system in development and disease. Adv. Anat. Embryol. Cell Biol..

[504] Pereira A., Fink G., Sundram S. (2009). Clozapine-Induced ERK1 and ERK2 Signaling in Prefrontal Cortex Is Mediated by the EGF Receptor. J. Mol. Neurosci..

[505] Aringhieri S., Kolachalam S., Gerace C., Carli M., Verdesca V., Brunacci M.G., Rossi C., Ippolito C., Solini A., Corsini G.U. (2017). Clozapine as the most efficacious antipsychotic for activating ERK 1/2 kinases: Role of 5-HT 2A receptor agonism. Eur. Neuropsychopharmacol..

[506] Missale C., Nash S.R., Robinson S.W., Jaber M., Caron M.G. (1998). Dopamine Receptors: From Structure to Function. Physiol. Rev..

[507] Neve K.A., Seamans J.K., Trantham-Davidson H. (2004). Dopamine Receptor Signaling. J. Recept. Signal Transduct..

[508] Nishi A., Shuto T. (2017). Potential for targeting dopamine/DARPP-32 signaling in neuropsychiatric and neurodegenerative disorders. Expert Opin. Ther. Targets.

[509] Bonci A., Hopf F.W. (2005). The Dopamine D2 Receptor: New Surprises from an Old Friend. Neuron.

[510] Emamian E.S., Hall D., Birnbaum M.J., Karayiorgou M., A Gogos J. (2004). Convergent evidence for impaired AKT1-GSK3β signaling in schizophrenia. Nat. Genet..

[511] Aringhieri S., Carli M., Kolachalam S., Verdesca V., Cini E., Rossi M., McCormick P.J., Corsini G.U., Maggio R., Scarselli M. (2018). Molecular targets of atypical antipsychotics: From mechanism of action to clinical differences. Pharmacol. Ther..

[512] Pereira A., Zhang B., Malcolm P., Sundram S. (2013). Clozapine regulation of p90RSK and c-Fos signaling via the ErbB1-ERK pathway is distinct from olanzapine and haloperidol in mouse cortex and striatum. Prog. Neuro-Psychopharmacol. Biol. Psychiatry.

[513] Mostaid S., Lee T.T., Chana G., Sundram S., Weickert C.S., Pantelis C., Everall I., Bousman C. (2017). Peripheral Transcription of NRG-ErbB Pathway Genes Are Upregulated in Treatment-Resistant Schizophrenia. Front. Psychiatry.

[514] Erben L., Welday J.P., Cronin M.E., Murphy R., Skirzewski M., Vullhorst D., Carroll S.L., Buonanno A. (2022). Developmental, neurochemical, and behavioral analyses of ErbB4 Cyt-1 knockout mice. J. Neurochem..

[515] Fu L., Jin W., Zhang J., Zhu L., Lu J., Zhen Y., Zhang L., Ouyang L., Liu B., Yu H. (2021). Repurposing non-oncology small-molecule drugs to improve cancer therapy: Current situation and future directions. Acta Pharm. Sin. B.

[516] Ansari Z., Pawar S., Seetharaman R. (2021). Neuroinflammation and oxidative stress in schizophrenia: Are these opportunities for repurposing?. Postgrad. Med..

[517] You F., Zhang C., Liu X., Ji D., Zhang T., Yu R., Gao S. (2021). Drug repositioning: Using psychotropic drugs for the treatment of glioma. Cancer Lett..

[518] Cohen B.M., Lipinski J.F. (1986). In vivo potencies of antipsychotic drugs in blocking alpha 1 noradrenergic and dopamine D2 receptors: Implications for drug mechanisms of action. Life Sci..

[519] Shin S.Y., Lee K.S., Choi Y.-K., Lim H.J., Lee H.G., Lim Y., Lee Y.H. (2013). The antipsychotic agent chlorpromazine induces autophagic cell death by inhibiting the Akt/mTOR pathway in human U-87MG glioma cells. Carcinogenesis.

[520] Shin S.Y., Kim C.G., Kim S.H., Kim Y.S., Lim Y., Lee Y.H. (2010). Chlorpromazine activates p21^Waf1/Cip1^gene transcription via early growth response-1 (Egr-1) in C6 glioma cells. Exp. Mol. Med..

[521] Lee M.S., Johansen L., Zhang Y., Wilson A., Keegan M., Avery W., Elliott P., Borisy A.A., Keith C.T. (2007). The Novel Combination of Chlorpromazine and Pentamidine Exerts Synergistic Antiproliferative Effects through Dual Mitotic Action. Cancer Res..

[522] Choi J.H., Yang Y.R., Lee S.K., Kim S.-H., Cha J.-Y., Oh S.-W., Ha J.-R., Ryu S.H., Suh P.-G. (2008). Potential Inhibition of PDK1/Akt Signaling by Phenothiazines Suppresses Cancer Cell Proliferation and Survival. Ann. N. Y. Acad. Sci..

[523] Matteoni S., Matarrese P., Ascione B., Buccarelli M., Ricci-Vitiani L., Pallini R., Villani V., Pace A., Paggi M.G., Abbruzzese C. (2021). Anticancer Properties of the Antipsychotic Drug Chlorpromazine and Its Synergism With Temozolomide in Restraining Human Glioblastoma Proliferation In Vitro. Front. Oncol..

[524] Fond G., Macgregor A., Attal J., Larue A., Brittner M., Ducasse D., Capdevielle D. (2012). Antipsychotic drugs: Pro-cancer or anti-cancer? A systematic review. Med. Hypotheses.

[525] Sachlos E., Risueño R.M., Laronde S., Shapovalova Z., Lee J.-H., Russell J., Malig M., McNicol J.D., Fiebig-Comyn A., Graham M. (2012). Identification of Drugs Including a Dopamine Receptor Antagonist that Selectively Target Cancer Stem Cells. Cell.

[526] Zhelev Z., Ohba H., Bakalova R., Hadjimitova V., Ishikawa M., Shinohara Y., Baba Y. (2004). Phenothiazines suppress proliferation and induce apoptosis in cultured leukemic cells without any influence on the viability of normal lymphocytes. Cancer Chemother. Pharmacol..

[527] Gil-Ad I., Shtaif B., Levkovitz Y., Dayag M., Zeldich E., Weizman A. (2004). Characterization of Phenothiazine-Induced Apoptosis in Neuroblastoma and Glioma Cell Lines: Clinical Relevance and Possible Application for Brain-Derived Tumors. J. Mol. Neurosci..

[528] Barygin O.I., Nagaeva E.I., Tikhonov D.B., Belinskaya D.A., Vanchakova N.P., Shestakova N.N. (2017). Inhibition of the NMDA and AMPA receptor channels by antidepressants and antipsychotics. Brain Res..

[529] Venkatesh H.S., Morishita W., Geraghty A.C., Silverbush D., Gillespie S.M., Arzt M., Tam L.T., Espenel C., Ponnuswami A., Ni L. (2019). Electrical and synaptic integration of glioma into neural circuits. Nature.

[530] Zeng Q., Michael I.P., Zhang P., Saghafinia S., Knott G., Jiao W., McCabe B.D., Galván J.A., Robinson H.P.C., Zlobec I. (2019). Synaptic proximity enables NMDAR signalling to promote brain metastasis. Nature.

[531] Xu F., Xia Y., Feng Z., Lin W., Xue Q., Jiang J., Yu X., Peng C., Luo M., Yang Y. (2019). Repositioning antipsychotic fluphenazine hydrochloride for treating triple negative breast cancer with brain metastases and lung metastases. Am. J. Cancer Res..

[532] (2009). Immune Control. Study of Fluphenazine in Relapsed or Relapsed-and-Refractory Multiple Myeloma. In ClinicalTrials.gov Identifier: NCT00821301. NCT00821301.

[533] National Cancer Institute (NCI) (2013). Fluphenazine in Treating Patients With Refractory Advanced Multiple Myeloma. In ClinicalTrials.gov Identifier: NCT00335647. NCT00335647.

[534] Kim H., Chong K., Ryu B.-K., Park K.-J., Yu M.O., Lee J., Chung S., Choi S., Kang S.-H. (2019). Repurposing Penfluridol in Combination with Temozolomide for the Treatment of Glioblastoma. Cancers.

[535] Kang S., Lee J.M., Jeon B., Elkamhawy A., Paik S., Hong J., Oh S.-J., Paek S.H., Lee C.J., Hassan A.H. (2018). Repositioning of the antipsychotic trifluoperazine: Synthesis, biological evaluation and in silico study of trifluoperazine analogs as anti-glioblastoma agents. Eur. J. Med. Chem..

[536] Chen X., Luo X., Cheng Y. (2018). Trifluoperazine prevents FOXO1 nuclear excretion and reverses doxorubicin-resistance in the SHG44/DOX drug-resistant glioma cell line. Int. J. Mol. Med..

[537] Krummel T.M., Neifeld J.P., Taub R.N. (1982). Effects of dopamine agonists and antagonists on murine melanoma:Correlation with dopamine binding activity. Cancer.

[538] Shaw V., Srivastava S., Srivastava S.K. (2021). Repurposing antipsychotics of the diphenylbutylpiperidine class for cancer therapy. Semin. Cancer Biol..

[539] Zhou W., Chen M.-K., Yu H.-T., Zhong Z.-H., Cai N., Chen G.-Z., Zhang P., Chen J.-J. (2015). The antipsychotic drug pimozide inhibits cell growth in prostate cancer through suppression of STAT3 activation. Int. J. Oncol..

[540] Hong J.-H., Kang S., Sa J.K., Park G., Oh Y.T., Kim T.H., Yin J., Kim S.S., D’Angelo F., Koo H. (2021). Modulation of Nogo receptor 1 expression orchestrates myelin-associated infiltration of glioblastoma. Brain.

[541] Lee J.-K., Chang N., Yoon Y., Yang H., Cho H., Kim E., Shin Y., Kang W., Oh Y.T., Mun G.I. (2015). USP1 targeting impedes GBM growth by inhibiting stem cell maintenance and radioresistance. Neuro-Oncology.

[542] Zhu Y., Zhao Y., Liu R., Xiong Y., Shen X., Wang Y., Liang Z. (2019). Olanzapine induced autophagy through suppression of NF-κB activation in human glioma cells. CNS Neurosci. Ther..

[543] Wiklund E.D., Catts V.S., Catts S.V., Ng T.F., Whitaker N.J., Brown A.J., Lutze-Mann L.H. (2010). Cytotoxic effects of antipsychotic drugs implicate cholesterol homeostasis as a novel chemotherapeutic target. Int. J. Cancer.

[544] Dilly S.J., Clark A.J., Marsh A., Mitchell D.A., Cain R., Fishwick C.W., Taylor P.C. (2017). A chemical genomics approach to drug reprofiling in oncology: Antipsychotic drug risperidone as a potential adenocarcinoma treatment. Cancer Lett..

[545] Hicks J.K., Sangkuhl K., Swen J.J., Ellingrod V.L., Müller D.J., Shimoda K., Bishop J.R., Kharasch E.D., Skaar T.C., Gaedigk A. (2017). Clinical pharmacogenetics implementation consortium guideline (CPIC) for CYP2D6 and CYP2C19 genotypes and dosing of tricyclic antidepressants: 2016 update. Clin. Pharmacol. Ther..

[546] Lamas D.J.M., Croci M., Carabajal E., Crescenti E.J.V., Sambuco L., A Massari N., Bergoc R.M., Rivera E.S., A Medina V. (2013). Therapeutic potential of histamine H_4_receptor agonists in triple-negative human breast cancer experimental model. Br. J. Pharmacol..

[547] Massari N.A., Medina V.A., Cricco G.P., Lamas D.J.M., Sambuco L., Pagotto R., Ventura C., Ciraolo P.J., Pignataro O., Bergoc R.M. (2013). Antitumor activity of histamine and clozapine in a mouse experimental model of human melanoma. J. Dermatol. Sci..

[548] Massari N.A., Nicoud M.B., Sambuco L., Cricco G.P., Lamas D.J.M., Ducloux M.V.H., Blanco H., Rivera E.S., Medina V.A. (2017). Histamine therapeutic efficacy in metastatic melanoma: Role of histamine H4 receptor agonists and opportunity for combination with radiation. Oncotarget.

[549] Shamir A., Kwon O.-B., Karavanova I., Vullhorst D., Leiva-Salcedo E., Janssen M.J., Buonanno A. (2012). The Importance of the NRG-1/ErbB4 Pathway for Synaptic Plasticity and Behaviors Associated with Psychiatric Disorders. J. Neurosci..

[550] Driver J.A., Logroscino G., Buring J.E., Gaziano J.M., Kurth T. (2007). A Prospective Cohort Study of Cancer Incidence Following the Diagnosis of Parkinson’s Disease. Cancer Epidemiol. Biomark. Prev..

[551] Sarkar C., Chakroborty D., Chowdhury U.R., Dasgupta P.S., Basu S. (2008). Dopamine Increases the Efficacy of Anticancer Drugs in Breast and Colon Cancer Preclinical Models. Clin. Cancer Res..

[552] Gemignani F., Landi S., Moreno V., Gioia-Patricola L., Chabrier A., Guino E., Navarro M., Cambray M., Capellà G., Canzian F. (2005). Polymorphisms of the Dopamine Receptor Gene *DRD2* and Colorectal Cancer Risk. Cancer Epidemiol. Biomark. Prev..

[553] Csatary L. (1972). CHLORPROMAZINES AND CANCER. Lancet.

[554] Barak Y., Achiron A., Mandel M., Mirecki I., Aizenberg D. (2005). Reduced cancer incidence among patients with schizophrenia. Cancer.

[555] Goldacre M.J., Kurina L.M., Wotton C.J., Yeates D., Seagroatt V. (2005). Schizophrenia and cancer: An epidemiological study. Br. J. Psychiatry.

[556] Diamandis P., Sacher A.G., Tyers M., Dirks P.B. (2009). New drugs for brain tumors? Insights from chemical probing of neural stem cells. Med. Hypotheses.

[557] Chou F.H.-C., Tsai K.-Y., Su C.-Y., Lee C.-C. (2011). The incidence and relative risk factors for developing cancer among patients with schizophrenia: A nine-year follow-up study. Schizophr. Res..

[558] Shchors K., Massaras A., Hanahan D. (2015). Dual Targeting of the Autophagic Regulatory Circuitry in Gliomas with Repurposed Drugs Elicits Cell-Lethal Autophagy and Therapeutic Benefit. Cancer Cell.

[559] Huang J., Zhao D., Liu Z., Liu F. (2018). Repurposing psychiatric drugs as anti-cancer agents. Cancer Lett..

[560] Walker A., Card T., Bates T., Muir K. (2010). R. Tricyclic antidepressants and the incidence of certain cancers: A study using the GPRD. Br. J. Cancer.

[561] De Bartolomeis A., Barone A., Begni V., Riva M.A. (2022). Present and future antipsychotic drugs: A systematic review of the putative mechanisms of action for efficacy and a critical appraisal under a translational perspective. Pharmacol. Res..

[562] Jobin M.-L., De Smedt-Peyrusse V., Ducrocq F., Baccouch R., Oummadi A., Pedersen M.H., Medel-Lacruz B., Angelo M.-F., Villette S., Van Delft P. (2023). Impact of membrane lipid polyunsaturation on dopamine D2 receptor ligand binding and signaling. Mol. Psychiatry.

[563] Salmas R.E., Yurtsever M., Durdagi S. (2016). Atomistic molecular dynamics simulations of typical and atypical antipsychotic drugs at the dopamine D2 receptor (D2R) elucidates their inhibition mechanism. J. Biomol. Struct. Dyn..

[564] Zhuang Y., Xu P., Mao C., Wang L., Krumm B., Zhou X.E., Huang S., Liu H., Cheng X., Huang X.-P. (2021). Structural insights into the human D1 and D2 dopamine receptor signaling complexes. Cell.

[565] Howes O., Egerton A., Allan V., McGuire P., Stokes P., Kapur S. (2009). Mechanisms Underlying Psychosis and Antipsychotic Treatment Response in Schizophrenia: Insights from PET and SPECT Imaging. Curr. Pharm. Des..

[566] Wakamori M., Kaneda M., Oyama Y., Akaike N. (1989). Effects of chlordiazepoxide, chlorpromazine, diazepam, diphenylhydantoin, flunitrazepam and haloperidol on the voltage-dependent sodium current of isolated mammalian brain neurons. Brain Res..

[567] Chen W., Zhu F., Guo J., Sheng J., Li W., Zhao X., Wang G., Li K. (2014). Chronic haloperidol increases voltage-gated Na+ currents in mouse cortical neurons. Biochem. Biophys. Res. Commun..

[568] Föhr K.J., Rapp M., Fauler M., Zimmer T., Jungwirth B., Messerer D.A.C. (2022). Block of Voltage-Gated Sodium Channels by Aripiprazole in a State-Dependent Manner. Int. J. Mol. Sci..

[569] Yatham L.N., Liddle P.F., Gonzalez M., Saraf G., Vafai N., Lam R.W., Sossi V. (2022). A Positron Emission Tomography Study of Dopamine Transporter Density in Patients With Bipolar Disorder With Current Mania and Those With Recently Remitted Mania. JAMA Psychiatry.

[570] Mereu M., Contarini G., Buonaguro E., Latte G., Managò F., Iasevoli F., de Bartolomeis A., Papaleo F. (2017). Dopamine transporter (DAT) genetic hypofunction in mice produces alterations consistent with ADHD but not schizophrenia or bipolar disorder. Neuropharmacology.

[571] Amato D., Kruyer A., Samaha A.-N., Heinz A. (2019). Hypofunctional Dopamine Uptake and Antipsychotic Treatment-Resistant Schizophrenia. Front. Psychiatry.

[572] Zahid U., Onwordi E.C., Hedges E.P., Wall M.B., Modinos G., Murray R.M., Egerton A. (2023). Neurofunctional correlates of glutamate and GABA imbalance in psychosis: A systematic review. Neurosci. Biobehav. Rev..

[573] Kruse A.O., Bustillo J.R. (2022). Glutamatergic dysfunction in Schizophrenia. Transl. Psychiatry.

[574] Mayeli A., Sonnenschein S.F., Yushmanov V.E., Wilson J.D., Blazer A., Foran W., Perica M., Calabro F.J., Luna B., Hetherington H.P. (2022). Dorsolateral Prefrontal Cortex Glutamate/Gamma-Aminobutyric Acid (GABA) Alterations in Clinical High Risk and First-Episode Schizophrenia: A Preliminary 7-T Magnetic Resonance Spectroscopy Imaging Study. Int. J. Mol. Sci..

[575] Bonoldi I., Howes O. (2013). The Enduring Centrality of Dopamine in the Pathophysiology of Schizophrenia. Adv. Pharmacol..

[576] Kesby J., Eyles D., McGrath J., Scott J. (2018). Dopamine, psychosis and schizophrenia: The widening gap between basic and clinical neuroscience. Transl. Psychiatry.

[577] Garritsen O., van Battum E.Y., Grossouw L.M., Pasterkamp R.J. (2023). Development, wiring and function of dopamine neuron subtypes. Nat. Rev. Neurosci..

[578] Parr A.C., Calabro F., Tervo-Clemmens B., Larsen B., Foran W., Luna B. (2022). Contributions of dopamine-related basal ganglia neurophysiology to the developmental effects of incentives on inhibitory control. Dev. Cogn. Neurosci..

[579] Stephan K.E., Friston K., Frith C. (2009). Dysconnection in Schizophrenia: From Abnormal Synaptic Plasticity to Failures of Self-monitoring. Schizophr. Bull..

[580] German D.C., Manaye K.F. (1993). Midbrain dopaminergic neurons (nuclei A8, A9, and A10): Three-dimensional reconstruction in the rat. J. Comp. Neurol..

[581] Moya N.A., Yun S., Fleps S.W., Martin M.M., Nadel J.A., Beutler L.R., Zweifel L.S., Parker J.G. (2022). The effect of selective nigrostriatal dopamine excess on behaviors linked to the cognitive and negative symptoms of schizophrenia. Neuropsychopharmacology.

[582] Feemster J.C., Westerland S.M., Gossard T.R., Steele T.A., Timm P.C., Jagielski J.T., Strainis E., McCarter S.J., Hopkins S.C., Koblan K.S. (2023). Treatment with the novel TAAR1 agonist ulotaront is associated with reductions in quantitative polysomnographic REM sleep without atonia in healthy human subjects: Results of a post-hoc analysis. Sleep Med..

[583] Halff E.F., Rutigliano G., Garcia-Hidalgo A., Howes O.D. (2022). Trace amine-associated receptor 1 (TAAR1) agonism as a new treatment strategy for schizophrenia and related disorders. Trends Neurosci..

[584] Krasavin M., Lukin A., Sukhanov I., Gerasimov A.S., Kuvarzin S., Efimova E.V., Dorofeikova M., Nichugovskaya A., Matveev A., Onokhin K. (2022). Discovery of Trace Amine-Associated Receptor 1 (TAAR1) Agonist 2-(5-(4′-Chloro-[1,1′-biphenyl]-4-yl)-4*H*-1,2,4-triazol-3-yl)ethan-1-amine (LK00764) for the Treatment of Psychotic Disorders. Biomolecules.

[585] Kane J.M. (2022). A New Treatment Paradigm. J. Clin. Psychopharmacol..

[586] Sauder C., Allen L.A., Baker E., Miller A.C., Paul S.M., Brannan S.K. (2022). Effectiveness of KarXT (xanomeline-trospium) for cognitive impairment in schizophrenia: Post hoc analyses from a randomised, double-blind, placebo-controlled phase 2 study. Transl. Psychiatry.

[587] Bach A., Clausen B.H., Møller M., Vestergaard B., Chi C.N., Round A., Sørensen P.L., Nissen K.B., Kastrup J.S., Gajhede M. (2012). A high-affinity, dimeric inhibitor of PSD-95 bivalently interacts with PDZ1-2 and protects against ischemic brain damage. Proc. Natl. Acad. Sci. USA.

[588] Kristensen M., Kucharz K., Fernandes E.F.A., Strømgaard K., Nielsen M.S., Helms H.C.C., Bach A., Tofte-Hansen M.U., Garcia B.I.A., Lauritzen M. (2020). Conjugation of Therapeutic PSD-95 Inhibitors to the Cell-Penetrating Peptide Tat Affects Blood–Brain Barrier Adherence, Uptake, and Permeation. Pharmaceutics.

[589] Bach A., Pedersen S.W., Dorr L.A., Vallon G., Ripoche I., Ducki S., Lian L.-Y. (2015). Biochemical investigations of the mechanism of action of small molecules ZL006 and IC87201 as potential inhibitors of the nNOS-PDZ/PSD-95-PDZ interactions. Sci. Rep..

[590] Therapeutics K. (2022). An Extension Study to Assess Long-Term Safety and Tolerability of Adjunctive KarXT in Subjects With Inadequately Controlled Symptoms of Schizophrenia. In ClinicalTrials.gov Identifier: NCT05304767. NCT05304767.

